# Global-scale population genetic analysis of *Plasmodium falciparum* identifies region-specific patterns of malaria parasite adaptation

**DOI:** 10.1038/s41467-026-73006-2

**Published:** 2026-05-11

**Authors:** Nina Billows, Jamille G. Dombrowski, Joseph Thorpe, Leen Vanheer, Sophie Moss, Jesse Gitaka, Colin J. Sutherland, Claudio R. F. Marinho, Nguyen Thi Hong Ngoc, Nguyen Thi Huong Binh, Nguyen Quang Thieu, Susana Campino, Taane G. Clark

**Affiliations:** 1 Faculty of Infectious and Tropical Diseases, London School of Hygiene and Tropical Medicine, London, United Kingdom; 2 Department of Parasitology, Institute of Biomedical Sciences, University of São Paulo, São Paulo, Brazil; 3 Directorate of Research and Innovation, Mount Kenya University, Gen. Kago Rd, Thika, Kenya; 4 UK Health Security Agency Malaria Reference Laboratory, LSHTM, London, WC1E 7HT UK; 5 Molecular Biology Department, Parasitology and Entomology, Vietnam National Institute of Malariology, Hanoi, Vietnam; 6 Faculty of Epidemiology and Population Health, London School of Hygiene and Tropical Medicine, London, United Kingdom

**Keywords:** Genetic variation, Parasite genomics

## Abstract

Malaria, caused by *Plasmodium* parasites, kills 610,000 people annually. The increasing availability of large-scale whole-genome sequencing (WGS) datasets enables investigation of parasite genetic diversity, population structure, and the spread of drug resistance. To investigate *Plasmodium falciparum* evolution, we analyse 17,565 isolates (17,489 previously published; 76 newly sequenced) from 39 countries, using identity-by-descent (IBD) networks,  population structure inference, and selection scans. Analyses reveal distinct patterns of selection, transmission, and drug resistance. Selection analyses identify genomic regions across multiple metrics, encompassing established and emerging drug-resistance loci. These patterns highlight region-specific adaptive processes within Southeast Asia. IBD analyses reveal recent haplotype sharing that shows strong local selection. New WGS from Brazil show increased IBD around *pfcrt*, consistent with local drug-selection pressures. Similarly, isolates from Vietnam exhibit high relatedness and elevated IBD around *pfKIC3*, with all isolates carrying the *pfkelch13* C580Y artemisinin resistance mutation. Elevated IBD in *pfKIC7* and *pfKIC9* in South America and the Horn of Africa suggests alternative pathways for future artemisinin resistance. Together, these findings highlight the genomic basis of *P. falciparum* adaptation and offer improved resolution for genomic surveillance of evolving parasite populations.

## Abstract (web summary)

In this study, the authors analyse 17,565 Plasmodium falciparum genomes from 39 countries to show global patterns of parasite population structure, drug-resistance spread and positive selection, providing improved resolution for genomic surveillance of malaria parasite adaptation worldwide.

## Introduction

Malaria, a vector-borne disease caused by *Plasmodium* parasites and transmitted by *Anopheles* mosquitoes, remains one of the most significant global public health challenges. Among the six *Plasmodium* species that infect humans, *Plasmodium falciparum* causes the most severe disease and accounts for the majority of malaria-related deaths, particularly among young children in sub-Saharan Africa. In 2024 alone, global estimates reached 282 million malaria cases and 610,000 deaths, underscoring the persistent burden of this disease despite decades of control efforts^1^.

While progress has been made through antimalarial treatments and vector control strategies, multiple biological threats have emerged that compromise their effectiveness^2,3^. These include the evolution of drug resistance in the parasite and insecticide resistance in mosquito vectors. In addition, the influence of global warming is increasingly recognised as a major factor shaping malaria transmission patterns, altering seasonality, changing human migration patterns, and potentially expanding the geographic range of the disease^4^. Together, these challenges highlight the need for a coordinated global response to control malaria and progress towards its eradication. A critical component of this response is addressing the biological processes that undermine current interventions. In particular, understanding the genetic diversity and evolutionary dynamics of *P. falciparum* enables tracking of the emergence and spread of drug resistance. While much of this work has focused on national or regional scales, expansion of efforts to map the evolutionary trajectories of *P. falciparum* to a global level can anticipate resistance trends and hasten an effective response to the waning effectiveness of malaria drug regimens.

The evolution of *P. falciparum* reflects a complex interplay of ecological, biological and anthropogenic factors. In addition, malaria control interventions have imposed strong selective pressures, causing population bottlenecks and regional differences in diversity^5–9^. Regional differences in *P. falciparum* population structure are well documented. African populations show high diversity due to intense transmission, while lower diversity is observed in South America and intermediate levels in Southeast Asia. These patterns align with region-specific antimalarial drug use and resistance histories^10–13^. Key resistance markers, such as mutations in *pfcrt* (chloroquine resistance transporter; chloroquine), *pfmdr1* (multi-drug resistance; amodiaquine, chloroquine, mefloquine, piperaquine), *pfdhfr* (dihydrofolate reductase; pyrimethamine), *pfdhps* (dihydropteroate synthase; sulfadoxine) and *pfkelch13* (Kelch propeller protein; artemisinin), exhibit geographic variation, but current panels remain incomplete^14–18^. These resistance markers include both single-nucleotide polymorphisms (SNPs) and structural or copy number variants, all of which exhibit regional variation in prevalence and pattern^19^. Continued identification of novel mutations across a wider panel of drug-resistance candidate genes is critical for surveillance and guiding treatment^20^. Partial artemisinin resistance, first emerging in Southeast Asia, now poses a growing threat in Africa, with concerning signs of reduced artemisinin combination therapy (ACT) partner drug efficacy, such as lumefantrine resistance in Uganda^21,22^.

Population genomics is a powerful tool to dissect the genetic landscape of *P. falciparum* and uncover the mechanisms driving its evolution and adaptation. By combining classical statistical techniques and high-resolution genomic analyses, researchers can quantify nucleotide diversity, identify loci associated with drug resistance and population divergence, characterise population structure and genetic relatedness, and unravel complex infection patterns^23–26^. These approaches have significantly advanced our understanding of the connectivity of parasite populations, provided insights into gene flow and admixture, and shown how quickly *P. falciparum* can adapt in response to selective pressures^27–29^. Importantly, population genetics has provided a framework for inferring the origins and spread of drug-resistant mutations, as well as for analysing diversity in genes encoding vaccine targets, both of which are major contributors to intervention response^30^. The expansion of publicly accessible genomic datasets has further enhanced the scale and resolution of such analyses. Notably, the 2023 release of the Pf7 data resource by MalariaGEN included nearly 20,000 *P. falciparum* whole-genome sequences from 33 countries, which are utilised here alongside publicly available data^26,31^. This resource has since expanded to over 33,000 whole-genome sequences in the 2025 MalariaGEN Pf8 release from additional sampling periods and geographic locations^26,31^. Several research groups have also made substantial contributions to the growing repository of genomic data, including from formerly under-represented malaria-endemic countries, further enabling large-scale comparative analyses and global surveillance efforts^32–38^.

Leveraging the expanding wealth of publicly accessible genomic data, together with newly generated whole-genome sequencing (WGS) data from Brazil and Vietnam, this study uses large-scale SNP-based analyses to investigate *P. falciparum* population dynamics from a global perspective. While many of these samples have previously been examined in regional studies, earlier global analyses have largely focused on dataset curation and broad descriptive patterns. They have typically not incorporated deeper analytical layers, such as admixture inference, selection scans, or identity-by-descent (IBD), which provide complementary insights into the evolutionary processes and transmission structure shaping *P. falciparum* populations. Here, we apply these approaches to evaluate how global genetic variation contributes to regional differences in infection complexity, parasite diversity, and the emergence and spread of antimalarial drug resistance. By comparing evolutionary pressures and transmission dynamics across diverse geographic settings, we can assess how *P. falciparum* responds to heterogeneity in local intervention strategies. This framework also enables exploration of the genomic architecture of resistance, including evidence for polygenic interactions that may underpin adaptive responses. Together, these findings illustrate how regional drug use and transmission intensity drive *P. falciparum* evolution, emphasising the importance of region-specific genomic surveillance and tailored treatment policies to effectively manage resistance and support malaria elimination efforts. This global analysis expands on previous population genetic analyses, that focused on either regional-scale analyses or data releases. These studies are further detailed in Supplementary Data 1^26,31–35,38–53^.

## Results

### Genomic and geographic diversity of *P. falciparum* isolates

After quality control procedures, the final dataset comprised 17,565 *P. falciparum* isolates (Pf7: 14,848; Alternative Source: 2641 and Newly Sequenced: 76) from 39 countries across 9 regions (Africa (Central, East, Horn, West, South Central), Asia (South, Southeast), South America, Oceania) (Table 1). Most isolates originated from West Africa (33.3%) and Southeast Asia (33.0%), with smaller percentages from East Africa (9.1%), South America (8.9%), South Asia (8.8%), Central Africa (2.9%), Oceania (2.0%), South Central Africa (1.5%), and the Horn of Africa (0.5%). Notably, Ghana contributed a significant portion of the isolates (*n* = 2,845, 16.2%), while each of the other countries contributed less than 10% to the total sample set (Supplementary Data 2). A total of 3,600,488 high-quality genome-wide variants were identified, of which 1,717,351 (47.7%) biallelic SNPs with a population-specific minor allele frequency (MAF) > 0.001 met the criteria for downstream analyses. Most of these shortlisted SNPs were non-synonymous (57.5%). SNP diversity was comparable across all regions (median SNP π range: 8.24 × 10^−4^ – 1.66 × 10^−3^), with slightly lower estimates for South American samples (Table 1).

**Table 1:** Regional Summary of P. falciparum Genetic Diversity and Within-Host Complexity (*N* = 17,565)

Region	*N*	No. countries	%	F_WS_ Median (Range)	Median SNP Diversity (π)
West Africa	5854	13	33.33	0.95 (0.23–1.00)	1.62 × 10^−3^
Southeast Asia	5790	5	32.95	0.98 (0.43–1.00)	1.17 × 10^−3^
East Africa	1601	6	9.11	0.95 (0.25–1.00)	1.66 × 10^−3^
South America	1562	7	8.89	0.99 (0.60–1.00)	8.24 × 10^−4^
South Asia	1554	2	8.84	0.97 (0.42–1.00)	1.51 × 10^−3^
Central Africa	514	1	2.93	0.90 (0.31–1.00)	1.61 × 10^−3^
Oceania	343	2	1.95	0.97 (0.48–1.00)	1.25 × 10^−3^
South Central Africa	255	1	1.45	0.77 (0.32–1.00)	1.54 × 10^−3^
Horn of Africa	92	2	0.52	0.99 (0.61–1.00)	1.51 × 10^−3^

### Population structure and Admixture

**Fig. 1:**
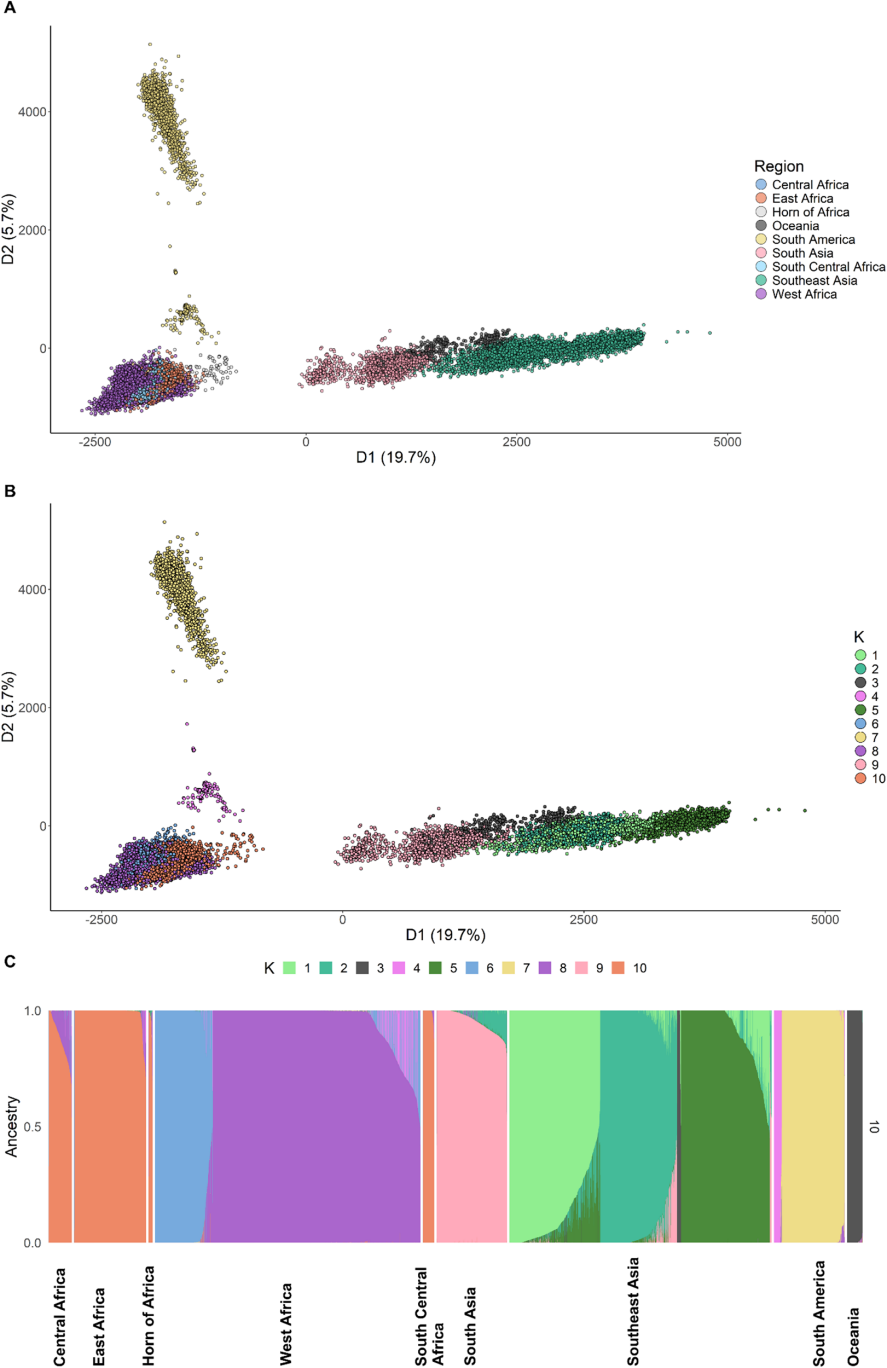
Global population structure and admixture of *P. falciparum* (*n* = 17,565). Multidimensional Scaling (MDS): Dimension 1 (D1) vs. Dimension 2 (D2) (**A**) demonstrates clustering across geographical regions. Points are coloured according to each isolate’s respective region. Admixture: Admixture analysis of samples from West Africa, the Horn of Africa, Central Africa, East Africa, South Central Africa, South America, Southeast Asia, South Asia and Oceania. MDS of *P. falciparum* coloured according to the ancestral population (K value) with the highest proportion for each isolate (**B**). Ancestry coefficients (y-axis) are plotted for each isolate (x-axis) belonging to each region for *K* = 10 ancestral populations (**C**). Source data are provided as a Source Data file.

Multidimensional scaling (MDS) was used to cluster genotypically similar isolates and showed that samples grouped according to their geographical regions and countries of origin (Fig. 1). Substantial overlap was observed among the African regions (Central, West, East, Horn of Africa and South Central Africa), as well as among the Asian-Oceanic regions (South Asia, Southeast Asia and Oceania) (Fig. 1). South American *P. falciparum* isolates display distinct regional structuring, reflecting geographic separation and historical transmission (Supplementary Fig. 1). Colombian and Ecuadorian isolates form a distinct cluster that is relatively close to African samples along the primary axis of genetic variation. Brazilian isolates displayed greater dispersion, overlapping with clusters from Africa, Colombia, and other South American regions. In contrast, isolates from Peru, French Guiana, Guyana and Venezuela group closely together to form a separate South American cluster. All South American and African clusters are clearly distinct from those in Southeast Asia, South Asia and Oceania (Supplementary Fig. 1).

To further investigate the geographical origins of *P. falciparum* isolates, ancestry inference was conducted using maximum likelihood estimation via ADMIXTURE analysis, based on 974,060 SNPs with a minor allele frequency (MAF) greater than 1%. The optimal number of ancestral populations (*K* = 10) was determined using cross-validation. The ADMIXTURE results revealed patterns of shared ancestry consistent with known geographic origins. For instance, four dominant ancestral clusters were observed among African isolates, with West Africa containing a distinct ancestral component compared to other African regions (Fig. 1). Similarly, Southeast Asian isolates exhibited four dominant ancestral populations, with shared components also observed with isolates from Oceania, reflected in three overlapping ancestral clusters. Isolates from South America and Southeast Asia exhibited more distinct and region-specific ancestry profiles relative to other parts of the world. At the country level, ancestral compositions were generally consistent within national borders, with overlapping patterns observed in neighbouring countries (Supplementary Figs. 2, 3). In contrast, isolates from Colombia and Ecuador had a unique ancestral composition, aligning with MDS findings and suggesting multiple introductions of *P. falciparum* into South America during historical periods of colonisation^5^. The Horn of Africa also exhibits limited gene flow from South Asia, alongside predominantly Central and East African ancestry (Supplementary Fig. 4).

### Within-host diversity

The F_WS_ statistic was employed to summarise relative inbreeding and within-host diversity, where higher values (closer to 1) indicate clonality and lower values (closer to 0) reflect a mixture of unrelated clones. An F_WS_ value greater than 0.95 typically signifies a monoclonal population. Regionally, the highest median F_WS_ values were observed in South America (0.99), Horn of Africa (0.99), Southeast Asia (0.98) and Oceania (0.97) (Table 1, Supplementary Fig. 5). These regions also had a higher percentage of isolates with F_WS_ > 0.95, indicating low multiplicity of infection (MOI). In contrast, lower median F_WS_ values and a smaller percentage of isolates with F_WS_ > 0.95were reported for South Central Africa (25.5%), Central Africa (41.2%), West Africa (49.0%), East Africa (48.5%), and South Asia (61.5%) (Supplementary Fig. 5).

At the country level, F_WS_ values fluctuated over time, which could suggest changes in transmission intensity. Statistically significant changes (*P* < 0.05) in within-host diversity across year groups were observed in Bangladesh (ANOVA *P* = 0.025), Cambodia (*P* = 0.025), Colombia (*P* = 0.021), The Gambia (*P* < 2×10^−16^), Ghana (*P* = 6.07 × 10^−6^), Kenya (*P* = 1.98 × 10^−8^), Laos (*P* = 2.31 × 10^−9^), Mali (*P* = 1 × 10^−4^), Myanmar (*P* = 0.001), Papua New Guinea (*P* = 0.003), Thailand (*P* = 9.21 × 10^−5^) and Vietnam (*P* = 2.33 × 10^−9^) (Supplementary Data 3). In several countries, rising F_WS_ scores over time may indicate declining transmission or increased sampling in areas of lower endemicity. Notable examples include Bangladesh, with a median F_WS_ increasing from 0.89 (range: 0.45–1.00) in 2008–2011 to 0.97 (range: 0.45–1.00) in 2016–2019; The Gambia, from 0.74 (range: 0.25–1.00) pre-2000 to 0.97 (range: 0.43–1.00) in 2016–2019; Ghana, from 0.86 (range: 0.29–1.00) in 2008–2011 to 0.89 (range: 0.25–1.00) in 2016–2019; and Laos, from 0.97 in 2008–2011 (range: 0.54–1.00) to 0.99 in 2016–2019 (range: 0.49–1.00). In other countries, F_WS_ scores showed variability and modest declines over time, potentially indicating rising transmission intensity. This pattern was observed in Benin, Cambodia, Guyana, Indonesia, Papua New Guinea and Vietnam (Supplementary Data 3). Notably, some countries experienced more pronounced declines in F_WS_ scores in recent years compared to earlier periods. For example, Mali saw a drop from a median F_WS_ of 0.93 (range: 0.34–1.00) in 2016–2019 to 0.73 (range: 0.48–0.99) in 2020–2021. Similarly, Kenya’s scores decreased from a median of 0.98 (range: 0.26–1.00) in 2012–2015 to 0.87 (range: 0.48–1.00) in 2016–2019. These shifts could reflect substantial increases in transmission or a focus on sampling in higher-transmission areas. However, in practice, F_WS_ values can vary substantially depending on whether samples are collected in clinical settings or through field-based household sampling, making direct comparisons challenging.

### Geographical distribution of drug-resistance markers

We first examined the geographical distribution of known genotypic markers associated with antimalarial drug resistance. Established drug resistance markers were obtained from the Malaria-Profiler database, and the WHO watchlist for *pfkelch13* artemisinin resistance markers^20,54^. High levels of genotypic resistance markers for chloroquine, pyrimethamine, and sulfadoxine were observed in South America (69.5%), followed by South Asia (67.0%), West Africa (62.2%), Central Africa (61.3%), East Africa (57.2%), Oceania (57.1%), South Central Africa (46.3%) and the Horn of Africa (40.3%) (Supplementary Figs. 6–13). In contrast, Southeast Asia showed the highest proportion of genotypes partially resistant to artemisinin in combination with these three drugs (40.1%), reflecting the region’s long-standing issue with multidrug-resistant *P. falciparum*. Given the global dependence on ACTs as the frontline treatment for *P. falciparum* malaria, the emergence and spread of artemisinin resistance poses a major threat to malaria control efforts. While artemisinin resistance remains most prevalent in Southeast Asia, genotypic markers were also detected in Oceania (*n* = 2), South Central Africa (*n* = 1), and South America (*n* = 1). The most detected artemisinin resistance-associated mutation was *pfkelch13* C580Y, present in 2270 isolates from Southeast Asia and in two samples from Oceania. Other notable *pfkelch13* variants observed in Southeast Asia included P441L (2%), R539T (2%), F446I (1%) and Y493H (1%) (Supplementary Fig. 6). Outside Southeast Asia, a single isolate from Zambia (South Central Africa) carried the P441L mutation, while the R561H variant, a validated marker of artemisinin resistance, was detected in an isolate from Guyana (South America). A mutation at the same codon position as C580Y, C580F, was also identified in one isolate from Myanmar, suggesting continued diversification of resistance-associated alleles in the region. Other mutations associated with partial artemisinin resistance have been detected through genotyping and targeted sequencing efforts in countries such as Uganda (R561H, A675V), Rwanda (R561H), Tanzania (C469Y, R561H), Kenya (C469Y, P574L) and Ethiopia (F446I, R662I, P574L); however, these mutations were not observed in the present dataset for these regions due to older samples in the dataset being represented^17,55–58^. We further searched for missense mutations in the propeller domain of Kelch13 (Supplementary Fig. 6). Fourteen mutations were uniquely observed in West Africa (N458D, E509D, V534L, A557S, T573S, E596G, E612D, G665S, E691D, L722V, C532S, V637I, V566I and V589I), while three additional mutations were also detected in East Africa (Y630F, I634L and S522C). Additionally, the *pfkelch13* mutations Q613E and R622T were identified in Central Africa and South Central Africa, respectively. However, all were reported at low frequency (< 5 samples total). The N458D mutation is located at the same amino acid position as N458Y, a validated marker of artemisinin resistance; however, N458D itself is not validated nor widely reported as being associated with resistance^59^. The *pfkelch13* A578S variant was observed in Central Africa (*n* = 2), East Africa (*n* = 5), South Asia (*n* = 5), South Central Africa (*n* = 1), and West Africa (*n* = 21), but is not considered linked to drug resistance and is known to be commonly found across Africa^60^. The *pfkelch13* S522C mutation has been reported in West Africa (*n* = 4) and East Africa (*n* = 1); although it has been associated with delayed parasite clearance, it has not been designated a candidate marker by the WHO due to limited supporting evidence^60,61^.

Using isolates with sufficient coverage across each drug-resistance locus, we examined the geographic distributions of haplotypes, as combinations of mutations capture the functional genetic backgrounds that influence both the presence and severity of resistance. We extended this analysis to additional candidate loci within relevant molecular pathways or showing signatures associated with emerging drug-resistance mechanisms (Supplementary Figs. 6–13). Across *pfcrt* (*PF3D7_0709000*), the CVIET haplotype (amino acid positions 73–76) was the most common, observed in 7125 samples predominantly from Africa, South Asia, and Southeast Asia (Supplementary Fig. 7). The SVMNT haplotype was also frequent (*n* = 1501) but restricted to South Asia, Oceania, and South America. A smaller number of samples carried CVMDT (*n* = 79), which was exclusive to South America. Additional missense mutations were detected that may influence the resistance phenotype through compensatory mechanisms, though functional validation is required. Of particular interest, C350R was detected only in samples from Guyana (*n* = 590). For *pfmdr1* (*PF3D7_0523000*; amino acid positions 86, 184, and 1246), two drug-resistant haplotypes were widespread: NFD (*n* = 7096) and YYD (*n* = 673), both found across multiple regions. A third haplotype, YFD (*n* = 261), was restricted to West, Central, and East Africa, whereas NFY was reported only in South America (*n* = 1158). For *pfdhfr* (*PF3D7_0417200*; positions 51, 59, and 108), 3449 samples from South and Southeast Asia carried the IRN haplotype in combination with I164L, which confers high-level resistance. The ICN haplotype was observed in South America (*n* = 1306) and Oceania (*n* = 223), along with C50R and S306F, respectively, suggesting possible compensatory roles. For *pfdhps* (*PF3D7_0810800*), the validated resistance allele A437G was detected in 14,460 samples across multiple regions. Among these, K450E was present in 6506 samples from South America, South Asia, Southeast Asia, and East Africa, whereas K450N occurred in 1869 samples from Southeast Asia. Additional mutations linked to regions with intensive drug use, including A613S (*n* = 80; South Central Africa and West Africa) and A613T (*n* = 32; Southeast Asia), were also observed.

Candidate loci associated with resistance to new drugs likewise exhibited notable variation. Seventeen mutations were identified in *pfcarl* (*PF3D7_0321900*), a putative resistance gene for ganaplacide, with Q605L and K903E most common across all regions. Missense mutations were also detected in the UDP-galactose transporter *PF3D7_1113300* (I4T, S18A, S169T; all rare) and in the acetyl-CoA transporter *PF3D7_1036800*, where T459I was common. Some variants in *PF3D7_1036800* showed geographic specificity, including N322Y in South America (*n* = 113) and K19T in Southeast Asia (*n* = 45). Although these candidate mutations lack functional validation, the diversity observed across known and putative drug-resistance genes highlights important loci for ongoing genomic surveillance.

### Population differentiation

To further investigate the genetic differentiation of *P. falciparum* across global populations, fixation index (F_ST_) analysis was conducted to identify highly differentiated genomic sites and assess patterns of divergence between regional subpopulations. A strong positive correlation was observed between genetic differentiation and geographic distance (Mantel *r* = 0.89, *P* = 0.001) (Supplementary Fig. 14). The lowest levels of genetic differentiation were observed within Africa (genome-wide F_ST_ range: 0.009–0.071), as well as between South and Southeast Asia (genome-wide F_ST_: 0.083). In contrast, the greatest differentiation was observed between Southeast Asia and South America (genome-wide F_ST_: 0.365), reflecting the large geographic separation between these regions (~18,327 km) and historically low human population movement between them (Supplementary Fig. 14). We also performed F_ST_ comparisons at the country level to observe inter- and intra-regional differences in genomic variation. Within regions, country-level comparisons had significantly lower Hudson’s F_ST_ than across regions (Wilcoxon rank-sum test, *P* < 2.2 × 10^−16^). However, across South America, Hudson’s F_ST_ between countries was higher than other intra-regional comparisons, especially when comparing Brazil vs. Ecuador, Colombia vs. Brazil, Colombia vs. French Guiana, Colombia vs. Guyana, Ecuador vs. Guyana, French Guiana vs. Ecuador, Peru vs. Colombia, and Peru vs. Ecuador (Median F_ST_:0.50; Range:0.36–0.68). This further reflects the distinct ancestral components observed for these countries. Across the African continent, Hudson’s F_ST_ was markedly lower, especially when comparing Central Africa (Democratic Republic of Congo) to countries from East Africa, South Central Africa, and West Africa (Median F_ST_: 0.02; Range: 0.01–0.02). Given the widespread transmission of *P. falciparum* across Africa, gene flow among these populations likely reduces genetic differentiation between countries in these regions.

We focused on assessing genetic differentiation using regional-level F_ST_ rather than country-level comparisons to capture more meaningful population structure and avoid noise arising from small or uneven sample sizes within individual countries. Patterns of high differentiation (F_ST_ > 0.75 and F_ST_ > 0.95) broadly aligned with geographic distance. The greatest number of highly differentiated SNPs (F_ST_ > 0.75) occurred in comparisons involving South America: with Southeast Asia (*n* = 172, including 41 with F_ST_ > 0.95), South Asia (*n* = 159, including 10 with F_ST_ > 0.95) and Oceania (*n* = 82, including 32 with F_ST_ > 0.95). These patterns support the strong influence of geographic distance on genotypic divergence. In contrast, no SNPs with extreme differentiation (F_ST_ > 0.95) were identified between South and Southeast Asia, consistent with their geographical proximity (average sampling distance: 1722 km) and long-standing human migration between the regions. Within Africa, only 14 highly differentiated SNPs (F_ST_ > 0.75) were identified across five genes: *RhopH2*, *PF3D7_1361800*, *PF3D7_0811600*, *Gcalpha*, and *PF3D7_0526600* (Supplementary Data 4), reflecting the lower overall genetic structure across the continent.

A total of 1346 SNPs exhibited high genetic differentiation (F_ST_ > 0.75) in intercontinental comparisons (Supplementary Data 4), highlighting key genomic regions underlying geographical divergence, including loci implicated in drug resistance and parasite transmission. Noteworthy examples include *pfdhfr* (pyrimethamine resistance), *pfmdr1* (chloroquine and mefloquine resistance), and *pfcrt* (chloroquine resistance), all of which harbour mutations known to mediate resistance phenotypes. Although no significant Gene Ontology (GO) terms were identified for biological processes or molecular functions, SNPs with F_ST_ > 0.75 were found within genes localised to cellular components, including the intrinsic component of the external side of the plasma membrane, anchored components of the plasma membrane, the apical complex, cell surface and apical part of the cell (Supplementary Data 5). Gene families also contained highly differentiated SNPs, particularly those involved in lipid scavenging, such as the ACS family (*n* = 38), which have been proposed as drug targets, but are also highly variable^62^. Additional high-F_ST_ SNPs were observed in genes mediating parasite-host and parasite-vector interactions, including *P47* (*n* = 33), *CTRP* (*n* = 20), and *Pfs16* (*n* = 9), or genes implicated in immune evasion, cellular invasion, or gametocyte development, and are considered vaccine candidates^26,63^. Comparable patterns were observed in country level pairwise F_ST_ analyses (Supplementary Data 6). Interestingly, despite their geographical proximity, Brazil and Ecuador showed a relatively high number of highly differentiated SNPs (F_ST_ > 0.75), a pattern that could be attributable to small sample sizes rather than true biological divergence. However, it could also reflect both geographic and sociolinguistic barriers to gene flow, including the lack of a shared border, distinct coastlines (Atlantic vs. Pacific) and differing colonial and linguistic histories that may limit parasite movement via human migration (Supplementary Fig. 1).

Further insights into regional differentiation were obtained using a ‘one-against-all’ F_ST_ approach. At the regional level, only 30 SNPs showed high genetic differentiation (F_ST_ > 0.75), and none exceeded an F_ST_ of 0.95 (Supplementary Data 7). Most of these high F_ST_ SNPs were specific to South American isolates (*n* = 17), with fewer identified in West Africa (*n* = 9), Oceania (*n* = 3) and Southeast Asia (*n* = 1). These loci were located in genes associated with drug resistance (*pfdhfr*, observed in Oceania), regulation of biological processes (*CRK3*, *SET1*), cellular metabolism (*ACS10*, *PF3D7_0709700*), biosynthesis (*PF3D7_0713600*, *PAIP1*), reproduction (*PF3D7_0809600*, *GIG*), nutrient uptake (*RhopH2*), cell localisation (*PF3D7_1440800*, *HSP101*), signalling (*GCbeta*), cell binding (*PF3D7_1410400*), and interspecies interactions (*P47*), as well as other genes with diverse functions (*PF3D7_1116800*, *PF3D7_1135100*, *PF3D7_1442200*). At the country level, the ‘one-against-all’ analysis identified 211 high F_ST_ SNPs, comprising 132 unique SNPs with F_ST_ > 0.75 and two SNPs with F_ST_ > 0.95 (Supplementary Data 8). Most of these SNPs were observed in isolates from Brazil, Ecuador, Colombia, Peru, and Indonesia, highlighting localised genetic structure. Several of these high F_ST_ SNPs overlapped with previously described genes and regions known to be highly variable, such as the *SURFIN* family, *pfcrt*, and *pfdhfr*. Notably, isolates from Brazil, French Guiana, Peru and Indonesia also carried highly differentiated SNPs in *FIKK4.2* and *FIKK10.1*, genes previously implicated in parasite invasion and virulence^64^. Together, these results emphasise region- and country-specific patterns of genetic differentiation in *P. falciparum*, which could be driven by a combination of geographic isolation, local selective pressures, and differing transmission dynamics.

### Genomic relatedness

Identity by descent (IBD) analysis was performed to identify genomic segments shared between *P. falciparum* isolates that arenot considered to have undergone recombination, serving as markers of inheritance from a recent common ancestor and providing a measure of genomic relatedness. The fraction of the genome classified as IBD was calculated using 10 kb sliding windows, and median IBD fractions (with ranges) were initially summarised by regional groupings (Supplementary Fig. 15). The highest IBD fractions were observed in samples from South America (median: 0.150; range: 0.003–0.663), followed by Southeast Asia (median: 0.055; range: 0.001–0.238), the Horn of Africa (median: 0.029; range: 0.001–0.227), Oceania (median: 0.028; range: 0.001–0.212), and South Central Africa (median: 0.012; range: 0.001–0.141). In contrast, the lowest IBD fractions were seen in South Asia (median: 0.002; range: <0.001–0.119), East Africa (median: 0.001; range: <0.001–0.114), Central Africa (median: 0.001; range: <0.001–0.114), and West Africa (median: 0.001; range: <0.001–0.100). These findings are consistent with patterns observed in F_WS_ scores and reflect the impact of higher transmission intensity in these regions. Similar trends were observed for pairwise IBD fractions, with South American samples exhibiting the highest levels of pairwise genomic relatedness (Supplementary Fig. 16).

Regions with higher IBD fractions may indicate reduced outcrossing within parasite populations, typically associated with low transmission intensity or geographic isolation. Conversely, lower IBD fractions suggest increased outcrossing due to higher transmission and greater genetic mixing between isolates. To further explore this, genomic regions with the highest IBD fractions were examined to identify associated genes and gene products (Supplementary Data 9). A total of 377 high-IBD segments (top 5% of IBD values) were identified, encompassing 173 genes. The highest counts of high-IBD segments were observed in the Horn of Africa and Oceania (*n* = 125 each), followed closely by East Africa (*n* = 122), South America (*n* = 121), South Asia and Southeast Asia (*n* = 120 each), West Africa (*n* = 120), Central Africa (*n* = 119), and South Central Africa (*n* = 117). Several segments were shared across multiple regions (Supplementary Fig. 17).

GO term analysis, which identifies functional categories over-represented within gene sets, showed that high-IBD regions across all geographic areas were significantly enriched for genes involved in protein-DNA complex subunit organisation, chromatin and chromosome organisation, and the regulation of transcription, translation, gene expression and biosynthetic processes (Supplementary Data 10). These genes were predominantly associated with chromosomal regions (fold change: 8.25; *n* = 5) and the nucleus (fold change: 1.59; *n* = 49), and their molecular functions were largely related to binding and structural molecule activity. Of particular interest, several high-IBD regions overlapped with loci associated with antimalarial drug resistance. These included *pfcrt* (in Central Africa, East Africa, Horn of Africa, Oceania, South America, South Asia, and West Africa), *pfdhps* (in East Africa, Horn of Africa, Oceania, South America, South Asia, South Central Africa, and Southeast Asia), and *pfmdr1* (in Central Africa, East Africa, Oceania, and South America), consistent with drug-driven selective pressures. Elevated IBD was also observed in regions encoding the Kelch13-interacting candidate genes *KIC7* (Horn of Africa and South America) and *KIC9* (South America), suggesting potential signatures of positive selection. Additionally, genes essential for parasite transmission and interaction with *Anopheles* mosquitoes, including *P47* (involved in immune evasion) and *P48/45* (linked to gamete fertility), showed high IBD in Oceania (Supplementary Data 9).

### Signals of selection vary across countries over time

We also examined signatures of recent positive selection using the iR statistic, which identifies loci with excess IBD sharing. The top five genes with the strongest selection signals (−log₁₀P > 5) for each country and time period are reported (Supplementary Fig. 18, Supplementary Data 11). Several genes consistently exhibited strong selection signals across multiple countries and time frames, including *ABCK1*, *AQP2*, *ARO*, *ATPase2*, *CAF1*, *CARM1*, *CG2*, *CRK3*, *pfcrt*, *CUL1*, *DHHC4*, *DRN1*, *JmjC1*, *MC-2TM*, and members of the *FIKK* family. Among these, *pfcrt* and *pfdhps* (*PPPK-DHPS*), particularly in Papua New Guinea (2012–2015), were notable for their well-established roles in antimalarial drug resistance. In addition to these canonical resistance genes, *ABCK1* (an ATP-binding cassette transporter) and *MC-2TM* (a multidrug and toxin extrusion transporter) also showed strong signals of selection, suggesting possible involvement in drug efflux mechanisms and warranting further functional investigation. Some genes showed evidence of recent positive selection in specific countries only, pointing to localised adaptation. These include *ARF-GAP* and *MCM3* (The Gambia, 2016–2019); *CDPK6*, *CDPK7*, *CK2α*, and *CLK3* (Kenya, 2016–2019); *DHHC9* (Laos, 2016–2019); *HAS1* (Benin, 2016–2019); *IMC1g* (The Gambia, 2016–2019); *LSA1* and *M712* (Zambia, 2016–2019); *NHE* (Guinea, 2016–2019); *P36* (Papua New Guinea, 2016–2019); *PF3D7_0201300* (Mozambique, 2016–2019); and *RAB7* (Guyana, 2020–2021). These genes are involved in key cellular processes, including metabolism, cell division, signal transduction, protein synthesis, membrane trafficking, and host-cell invasion. Several of the genes under country-specific selection, such as *LSA1* (Liver Stage Antigen 1) and *P36*, are potential malaria vaccine candidates. Evidence of selection at these loci may have implications for vaccine efficacy and highlights the need to monitor adaptive evolution in *P. falciparum* populations across geographic regions over time^65,66^.

### IBD patterns and SNP associations with *pfkelch13*-mediated resistance

While earlier IBD-based analyses provided insight into broad patterns of genetic relatedness, we sought to more precisely characterise transmission dynamics and the genomic backgrounds associated with *pfkelch13* mutations, to elucidate the genetic context underlying the emergence of partial artemisinin resistance. Using IBD to reconstruct recent shared ancestry, we mapped transmission networks and examined the genomic context of *pfkelch13* variants across parasite populations from Southeast Asia and Oceania (Supplementary Fig. 19). Overall, *pfkelch13* C580Y-positive samples showed significantly higher pairwise IBD fractions (median: 0.28) compared to samples with other *pfkelch13* missense mutations in the propeller domain (median: 0.06; Wilcoxon *P* < 2 × 10^−16^ (two-sided)) or wild-type alleles (median: 0.03; Wilcoxon *P* < 2 × 10^−16^ (two-sided)) (Supplementary Fig. 19). These findings support the hypothesis of clonal expansion of the C580Y genotype, particularly in the context of the KEL1/PLA1 lineage, a multidrug-resistant strain associated with piperaquine resistance^67^. To gain further insight, IBD networks were constructed using thresholds of 47.5% and 95% IBD (Supplementary Figs. 20–22). These thresholds were chosen to reflect broader regional transmission (47.5%) and more direct, local transmission (95%). While the convenience sampling approach may limit the ability to fully reconstruct historical transmission patterns, data were stratified into three epidemiologically relevant time windows: 2008–2011 (emergence of *pfkelch13* C580Y in western Cambodia), 2012–2015 (expansion of artemisinin resistance and the rise of KEL1/PLA1) and 2016–2019 (regional dominance of artemisinin resistance across Southeast Asia) (Supplementary Figs. 20–22).

At the 95% IBD threshold, samples consistently clustered by *pfkelch13* genotype across all time periods, indicating transmission of closely related parasites carrying the same resistance mutations. Clustering also occurred by country or site, suggesting either local transmission or the onward spread of imported cases. In contrast, at the 47.5% threshold, clusters frequently included a mix of *pfkelch13* genotypes, which could reflect recombination over time among genetically related parasites sharing similar genomic backgrounds. One example is a large cluster from years 2008–2011 containing three genotypes (*pfkelch13* C580Y, *pfkelch13* R539T, and wild-type) across Cambodia, Vietnam and Laos (Supplementary Fig. 20). Over time, both the number and geographic spread of *pfkelch13* C580Y-positive clusters increased, often spanning multiple countries. This trend may reflect the expansion and regional dominance of the KEL1/PLA1 lineage, although definitive conclusions are limited by the convenience sampling design.

The IBD analysis revealed clonal clusters of *pfkelch13* C580Y-positive parasites with extensive shared genomic regions, suggesting that additional loci may be under co-selection. To investigate this further, we performed genome-wide SNP association analyses that integrated co-occurrence patterns, genotypic correlations, and population-adjusted models to identify variants linked to the C580Y background across Southeast Asia (see **“Methods”**). Candidate mutations were prioritised into three confidence tiers: Tier 1 (high), Tier 2 (moderate) and Tier 3 (low, functional relevance-based) (see **“Methods”**). A total of 22 mutations across 20 genes met the Tier 1 criteria, showing strong support across multiple analyses, significant association (*P* < 0.0001), high linkage (*R²* > 0.5), and large effect sizes (an odds ratio (OR) in the 95th percentile) **(**Supplementary Data 12). This set included *ARPS10* V127M, a previously reported interactor with *pfkelch13* C580Y, validating our approach^43^. Another top-ranked variant, *MyoF* S969P (*PF3D7_1226000*, formerly *MyoC*), a component of the Kelch13-associated protein complex, further implicates changes in the parasite’s intracellular endocytosis architecture in facilitating resistance in the Greater Mekong sub-region. An additional 55 mutations across 53 genes were classified as Tier 2 (moderate confidence), including *pfcrt*, which has been linked to the *pfkelch13* C580Y background. This supports the idea that a pre-existing drug resistance background, such as chloroquine resistance, may have predisposed parasites to evolve artemisinin resistance. However, recent studies of Ugandan isolates with Kelch13-mediated reduced artemisinin susceptibility indicate that these have evolved on a background of wild-type *pfcrt* and complete susceptibility to chloroquine^21,22^. Tier 3 consisted of 20 mutations in 14 genes (*MDR2, DHFR-TS, UBP1, AP2-L, MDR1, MCA-2, AP2-G, VPS51, CRT, MyoC/MyoF, PF3D7_1365800, PF3D7_0907200, PF3D7_1329500*, and *PF3D7_1243400*) (Supplementary Data 12). These mutations show weaker statistical associations but were retained due to their potential functional relevance based on prior studies.

To investigate potential shared pathways, we constructed a protein–protein interaction (PPI) network using all prioritised candidates. Six clusters emerged, including one enriched for known drug resistance proteins (MDR1, MDR2, CRT, DHFR-TS) and associated genes (*PF3D7_0214600, PF3D7_0104300, PF3D7_1438500, OXA1, PF3D7_1017000, PF3D7_1331300*). This observation suggests that *PF3D7_0214600* may have contributed to the emergence of *pfkelch13* C580Y. Additional interactions were observed between *pfkelch13* and *PF3D7_0303800*, a Tier 2 candidate found in Laos, as well as among *PF3D7_0405400* (putative pre-mRNA processing-splicing factor 8) and *PF3D7_1119300* (splicing factor U2AF small subunit), which are involved in mRNA splicing, potentially influencing gene regulation and parasite fitness. Together, these associations highlight a complex genomic background linked to *pfkelch13* C580Y. While not all identified mutations may be functionally relevant, some may represent compensatory changes or modifiers that impact cellular pathways, gene expression or survival. Further functional validation is needed to delineate their roles in compensatory evolution and artemisinin resistance.

### Composite signals of positive selection in *P. falciparum*

To identify regions under positive selection in global *P. falciparum* populations, we applied complementary metrics capturing recent and population-specific signals, including elevated IBD, reduced Tajima’s D, extreme iHS (within population) and XP-EHH (across populations), and moderate-high F_ST_ values. A composite selection analysis was conducted in 10-kb windows by integrating IBD, Tajima’s D, iHS and XP-EHH (see **“Methods”**), together with SNPs showing moderate F_ST_, and regions were classified as exhibiting weak, moderate or strong support for selection (Supplementary Fig. 23). Across global populations, the number of genomic regions under selection varied (Supplementary Data 13). East Africa had the largest number of regions with signals of selection (Weak: 104, Moderate: 123, Strong: 11), encompassing 385 genes, followed by West Africa (Weak: 113, Moderate: 78, Strong: 26; 364 genes) and South Asia (Weak: 105, Moderate: 29, Strong: 33; 302 genes). Fewer regions showed evidence of selection in South America (Weak: 82, Moderate: 57, Strong: 0; 135 genes) and Southeast Asia (Weak: 111, Moderate: 24, Strong: 0; 200 genes). In contrast, signals of selection were more limited in South Central Africa (Weak: 97, Moderate: 8, Strong: 0; 172 genes), Central Africa (Weak: 59, Moderate: 43, Strong: 0; 141 genes) and Oceania (Weak: 58, Moderate: 11, Strong: 0; 119 genes). The Horn of Africa exhibited the fewest regions under selection (Weak: 50, Moderate: 0, Strong: 0), encompassing 72 genes, with iHS and XP-EHH excluded owing to limited sample size.

**Fig. 2:**
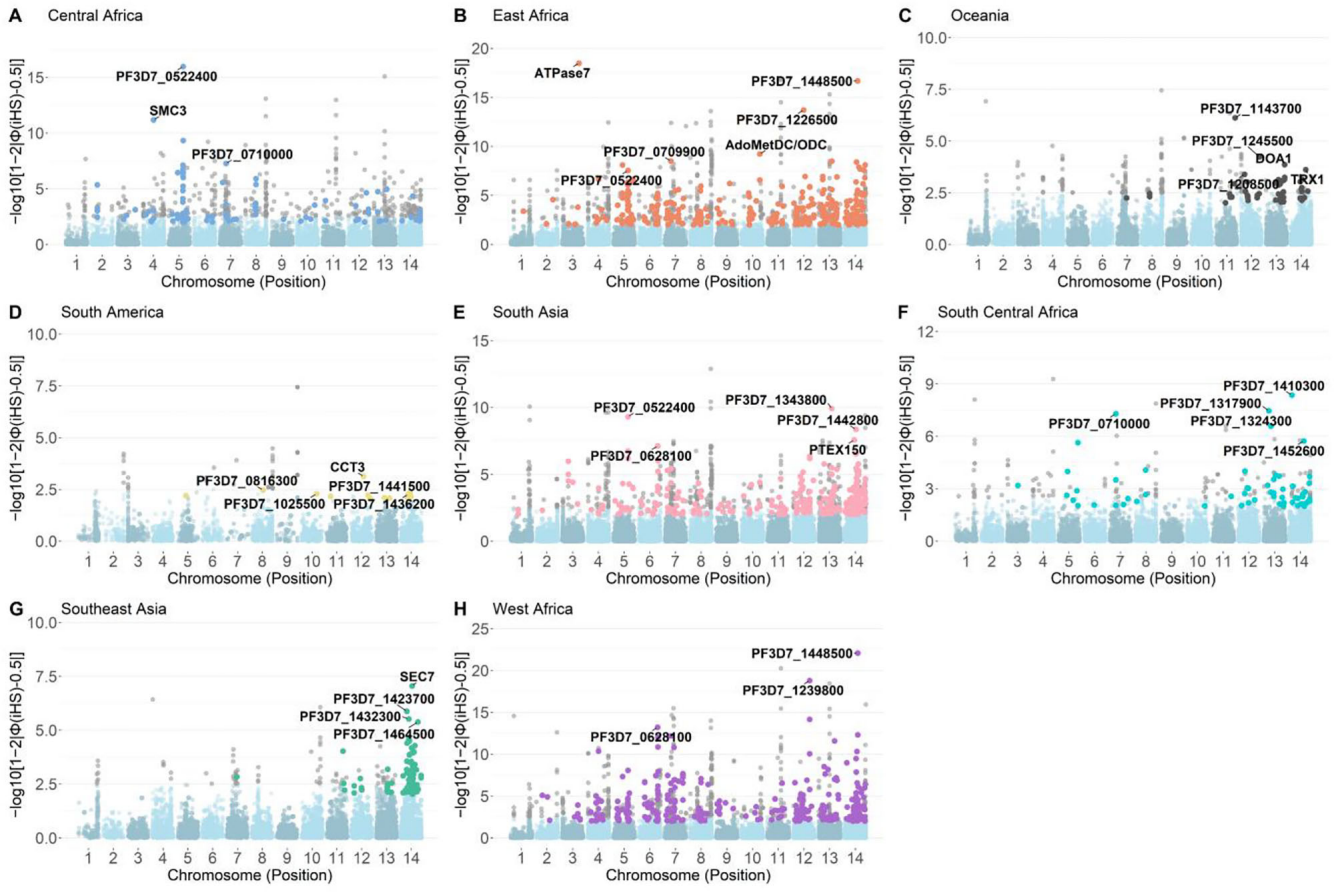
Genomic regions under recent positive selection within single regions (iHS score). Genomic regions under recent positive selection across eight geographical regions, including Central Africa (**A**), East Africa (**B**), Oceania (**C**), South America (D), South Asia (**E**), South Central Africa (**F**), Southeast Asia (**G**) and West Africa (**H**). Regions with |iHS| scores > 2.0 are highlighted and coloured according to each region. Genes are annotated if they contain the top 5 hits or a sustained peak with greater than 5 significant SNPs. The region of the Horn of Africa was excluded from the iHS analysis due to a small sample size. Candidate SNPs in genomic regions that fall within the top 10% with Tajima’s D (balancing selection) are highlighted in a neutral shade and were excluded in the second stage of analysis (see **“Methods”**). Source data are provided as a Source Data file.

Furthermore, global *P. falciparum* populations show selection targeting both parasite survival and host–parasite interactions (Fig. 2, Supplementary Data 14–17). Genes encoding proteins involved in host invasion and parasite-vector interactions featured prominently, including *PSSP17*/*PSOP17* (surface proteins; Oceania, South Asia), *RON2* and *TREP* (erythrocyte invasion; South America, South-Central Africa), and signal peptide-processing enzymes (*SPP*) that facilitate host-cell engagement. Beyond host interactions, selection also targeted core cellular functions, including transcription and chromatin regulation (*TAF10*, *BDP1*, *Myb2*), RNA splicing and processing (*BUD31*, *SF3B2*, *POP1*, *POP4*), DNA replication and cell-cycle control (*ORC2*, *MCM3*, *MCM7*), and essential metabolic and stress-response pathways (*SufA*, *PTPS*, *HAD1*, *HSP60*, *PPPK-DHPS*, *HSP90*). These loci showed moderate to strong signals across chromosomes 5–14 in multiple regions, supported by concordant haplotype-based metrics (Tajima’s D, iHS, IBD and XP-EHH). Collectively, these results indicate that positive selection consistently targets both fundamental cellular processes and genes central to host–parasite interactions, underscoring the evolutionary pressures shaping parasite survival and transmission.

We next identified genomic regions under selection that have previously been linked to antimalarial drug resistance (Supplementary Data 14). *PF3D7_0810800* (*pfdhps*; sulfadoxine resistance) showed moderate evidence of selection in East and West Africa and weaker signals in South Asia, South Central Africa, Central Africa, Oceania and the Horn of Africa. *PF3D7_1343700* (*pfkelch13*; partial artemisinin resistance) exhibited moderate selection signals in South and Southeast Asia. *PF3D7_1218300* (AP-2 complex subunit mu; putative Kelch13 pathway component) showed weak evidence in East and Central Africa. Two additional loci, *PF3D7_1036800* (acetyl-CoA transporter) and *PF3D7_1451100* (elongation factor 2, eEF2), displayed weak signals of selection in South Asia. Notably, *PF3D7_1036800* haplotypes, including N306K and T459I, were more prevalent in South Asia (74%, 20 samples) than in other regions (Supplementary Fig. 13). Collectively, these findings highlight widespread selection on *pfdhps* across Africa, the historically regional confinement of *pfkelch13* selection to Asia, and emerging signals at putative drug-resistance loci such as *PF3D7_1036800* and *PF3D7_1451100*.

F_ST_ analysis further identified differentiated SNPs in key drug–resistance genes across populations (Supplementary Data 15). In *PF3D7_0810800* (*pfdhps*; PPPK–DHPS), SNP Ala581Gly (550117C > G) showed moderate differentiation in West Africa (F_ST_ = 0.39), while SNP chromosome 8:547635T > C found upstream of *pfdhps* was strongly differentiated in Oceania (F_ST_ = 0.73), indicating substantial allele-frequency differences between Oceania and other populations. In *PF3D7_1343700* (*pfkelch13*; K13), SNP K189T (1726432T > G) showed moderate differentiation in Southeast Asia (F_ST_ = 0.39). This SNP is not implicated in partial artemisinin resistance and is observed globally. For *PF3D7_1218300* (AP-2 complex subunit mu), SNPs chromosome 12:718254A > C in East Africa (F_ST_ = 0.31) and K199T (718550A > C) in Central Africa (F_ST_ = 0.31) showed low to moderate differentiation, suggesting smaller allele-frequency differences across African populations.

Furthermore, several genes in this dataset exhibited selection signals potentially associated with *pfkelch13* C580Y genomic backgrounds in Southeast Asia (Supplementary Data 12). A region on chromosome 5 (910,000–940,000), encompassing *PF3D7_0522400*, showed multiple concordant signals of selection, including Tajima’s D, IBD and XP-EHH (South Asia vs Southeast Asia), consistent with both local selection and shared haplotypes across South and Southeast Asia. Nine sites within *PF3D7_0522400* had F_ST_ values > 0.3, further supporting population differentiation at this locus.

**Fig. 3:**
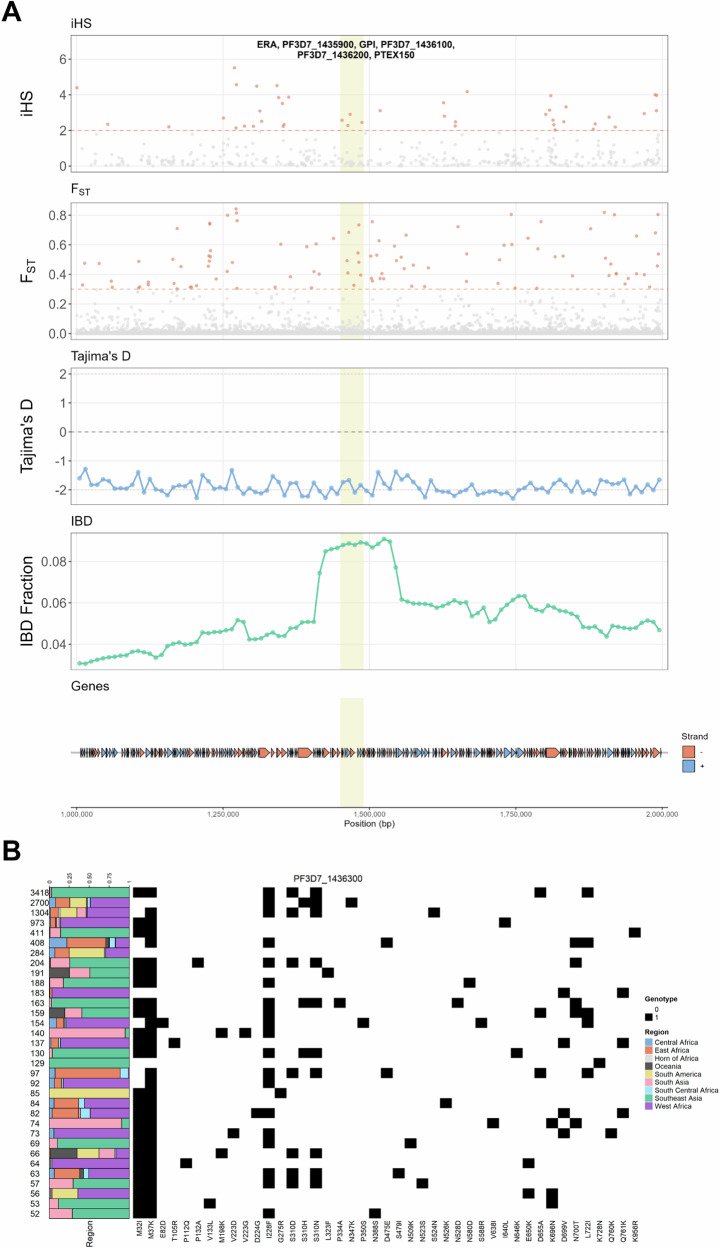
Candidate selective sweep on chromosome 14 encompassing *PTEX150* in Southeast Asia. Composite selection metrics across chromosome 14 (1,450,000–1,490,000 bp) are shown, including Tajima’s D, F_ST_, iHS, and IBD fraction, highlighting a region of elevated support overlapping *PTEX150* (**A**). Haplotype structure within this interval is shown for samples from Southeast Asia (*n* > 50), revealing a dominant shared haplotype (KFIAE) that is observed across the region, consistent with a candidate selective sweep (**B**). Source data are provided as a Source Data file.

On chromosome 12, *PF3D7_1223500* contained four sites with F_ST_ > 0.3 and was supported by both IBD and XP-EHH, indicating extended haplotype homozygosity and haplotype sharing consistent with selection. On chromosome 14, *PF3D7_1436300* (*PTEX150*) harboured a missense mutation (I228F) at position 1,480,458 with high differentiation (F_ST_ = 0.54). This is located within the chromosome 14: 1,450,000–1,490,000 region, which is supported by iHS and IBD, indicating selective pressure (Fig. 3). Although this SNP is observed across multiple geographic regions, several *PTEX150* haplotypes are restricted to Asia and the Pacific, where partial artemisinin resistance emerged. Notably, a common *PTEX150* haplotype, KFIAE (M37K, I228F, D655A, L722I and K827E), was observed across Southeast Asia (*n* = 2485), South Asia (*n* = 66) and Oceania (*n* = 21), both before and after the emergence of partial ACT resistance. Consistent with this, high iHS (5.56) was observed for *PTEX150* in Southeast Asia at position 1,480,458 on chromosome 14. The high IBD region also contains *PF3D7_1438400* (metacaspase-2, *MCA-2*), a member of the Kelch13 compartment. Another region of interest on chromosome 8 (1,120,000-1,129,999) showed low Tajima’s D and elevated IBD. This region contains *PF3D7_0826100*, a putative HECT-like E3 ubiquitin ligase (HEUL). Although not directly associated with the C580Y genotype, this gene is of interest given the established role of the ubiquitin-proteasome system in reduced artemisinin susceptibility. A missense mutation in *PF3D7_0826100* (I5835R; 1,121,641A > C) showed strong population differentiation in Southeast Asia (F_ST_ = 0.82), further supporting selection at this locus.

### Integration of new regional datasets into global population genomics

In this study, we generated 76 additional *P. falciparum* whole-genome sequences from Vietnam (*n* = 18) and Brazil (*n* = 58). The inclusion of Brazilian samples is particularly valuable, as *P. falciparum* from Brazil is currently absent from major curated global reference datasets, such as Pf7 and Pf8^26,31^. The samples from Brazil were collected across two timepoints and clinical groups, including pregnant women in 2013/2014 and the general population in 2020/2021. Due to the limited sample size available for the new data, no signals of selection (iHS or XP-EHH) could be detected with sufficient statistical power. However, F_WS_, F_ST_, IBD and analysis of drug-resistance mutations and haplotypes were performed.

Nearly all Brazilian isolates were monoclonal (F_WS_ > 0.95), with only two exceptions: one from 2013/2014 and one from 2020/2021. Samples collected in 2013/2014 showed a median IBD fraction of 0.28, compared with 0.23 for those collected in 2020/2021; these values were not statistically different. Genomic regions with elevated IBD were broadly consistent with patterns observed in other South American samples. Notably, however, a region on chromosome 7 (400001–410000) encompassing *pfcrt* exhibited high IBD (95th percentile) specifically in the 2020/2021 Brazilian samples (Supplementary Data 18). Genetic differentiation between the two Brazilian timepoints was modest (F_ST_ = 0.11). When combined, the Brazilian isolates were most similar to samples from French Guiana (F_ST_ = 0.31) and Guyana (F_ST_ = 0.32), and showed substantially higher differentiation from Ecuador (F_ST_ = 0.63). Seven SNPs had high differentiation between the 2013/2014 and 2020/2021 samples (F_ST_ > 0.75), including six in *PF3D7_0832000* (stevor) and one in *PF3D7_0113600* (SURFIN). Across comparisons with all other global samples, ten sites exhibited extreme differentiation (F_ST_ > 0.95), located in genes including *PF3D7_0321300*, *PF3D7_1211700* (mRNA–DNA replication licensing factor MCM5, putative), *PF3D7_0908200*, *PF3D7_1252400*, *PF3D7_1450200*, and *PF3D7_0208300* (Supplementary Data 8). Brazilian isolates carried drug-resistance haplotypes characteristic of other South American populations, including *pfcrt* SVMNT (*n* = 49), *pfdhfr* ICN (*n* = 58), and *pfdhps* K450E/A518G (*n* = 56). Despite this similarity, the combination of elevated IBD signals, timepoint-specific differentiation, and highly divergent SNPs points to distinctive genomic signatures unique to Brazil.

We also compared the newly generated Vietnam genomes with publicly available *P. falciparum* samples collected from the same country. The majority of new isolates were considered monoclonal (F_WS_ > 0.95, *n* = 16/18) and had a median pairwise IBD fraction of 0.54 (range: 0–0.93), indicating that many infections were moderately to highly related, with some pairs approaching clonal identity. As with the Brazil dataset, genomic regions with elevated IBD (95th percentile) were broadly consistent with patterns observed in other Southeast Asian populations. One region on chromosome 9 (610001–620000) was of particular interest, as it encompasses KIC3, a Kelch13 compartment protein implicated in artemisinin resistance (Supplementary Data 19). This high IBD signal coincides with the presence of the *pfkelch13* C580Y variant, which was detected in all samples with adequate coverage. Most isolates also carried the CVIET *pfcrt* haplotype (*n* = 10) and the IRN *pfdhfr* haplotype (*n* = 18). When comparing the new Vietnam samples to other Southeast Asian populations using F_ST_, five SNPs showed high differentiation, all located within *PF3D7_1301800* (SURFIN). Together, these findings indicate that the newly sequenced Vietnam samples are consistent with established regional population structure, while also revealing potential signatures of drug selection through elevated IBD and divergence at specific loci. The contribution of these isolates, in the context of previously published analyses, is further detailed in Supplementary Data 1^26,31–35,38–53^.

## Discussion

With the increasing threat of *P. falciparum* across the globe, substantial effort is required to achieve malaria elimination. Data-driven strategies and the development of new tools offer innovative approaches to aid the surveillance of *P. falciparum*. However, generating such tools necessitates a comprehensive understanding of *P. falciparum* on a global scale, which has recently become attainable through access to large-scale, publicly accessible data^26,31–36,38^. By delving further into the *P. falciparum* genome, we can gain greater insight into the biological mechanisms that threaten malaria control and enhance our understanding of population genetics. This large-scale genomic data allows for a deeper exploration of *P. falciparum*’s evolution, patterns of gene flow, and the impact of control efforts, offering critical information to guide malaria control strategies.

The evolution of *P. falciparum* has been dynamic, shaped significantly by human and vector migration, which has facilitated its global spread, and by control efforts, which have led to population reductions and bottlenecks^8^. Both historical migrations and recent interventions have left their marks, visible in the population structure analysis of *P. falciparum* genome-wide SNPs^9^*. P. falciparum* isolates cluster according to continental boundaries, with some clustering according to specific geographical regions such as South Asia, Southeast Asia, and Oceania, while African samples are less distinct. The clustering of some South American samples with African regions aligns with evidence suggesting multiple introductions of *P. falciparum* from Africa, including during the transatlantic slave trade^37,68^. ADMIXTURE analysis further highlights the shared ancestry between intercontinental and intracontinental regions. For example, as mentioned, South American samples share ancestry with West African populations^68^. However, they also exhibit a distinct genetic signature, which could be shaped by a population bottleneck during introduction and approximately 450 years of subsequent isolated evolution, local adaptation, or influence from ancestral lineages not represented in current West African samples. Consistent with multidimensional scaling analysis, African populations exhibited overlapping ancestry, with samples from East and Central Africa showing greater genetic similarity to each other than to those from West Africa. In contrast, the Horn of Africa forms a distinct genetic cluster, shaped by gene flow from African regions as well as some influence from Asia across the Indian Ocean and Arabian Sea. Outside Africa, there was partial overlap in ancestry between Southeast Asia, Oceania and South Asia, reflecting shared evolutionary history or gene flow, although each region retained distinct genetic signatures, indicative of region-specific selection or demographic events.

Further exploration of the relationship between genetic and geographic structure was carried out using F_ST_ analysis. Generally, genetic differentiation correlated with geographic distance, except in regions with smaller sample sizes, for which genetic differentiation was only evident at the intracontinental scale. Despite the high SNP diversity in African samples, they displayed the least genetic differentiation. This aligns with other analyses indicating a high degree of outcrossing within African populations, supported by higher MOI and lower IBD estimates. Meanwhile, comparisons between countries in South America had higher F_ST_ compared with other intraregional analyses. Although limited sample sizes may contribute to this pattern, the pronounced genetic differentiation between Ecuador and Colombia and the countries in the eastern Amazon basin, such as Brazil, French Guiana, and Guyana, is consistent with results from admixture analysis. These patterns align with known historical introduction routes and ecological and vector-related barriers that restrict parasite movement across the continent^5,69^. Genomic regions with high F_ST_ values provide insight into the drivers of genetic differentiation between geographical regions. Several genes harbouring high F_ST_ SNPs are known to play roles in transmission, including the gene encoding P47, a protein implicated in mosquito immune evasion, as well as P48/P45, which is essential for ookinete formation and transmission^70–72^. These genes and their products are proposed targets for transmission-blocking vaccines. Moreover, highly differentiating SNPs were detected in drug resistance genes, reflecting the administration of different drugs across various regions and time points. The widespread prevalence of drug resistance mutations across all regions is concerning and underscores the importance of global surveillance. SNPs with high F_ST_ may be utilised for geographical classification and molecular barcoding to support such surveillance efforts.

Despite the dataset being curated from samples sequenced over several decades, the results from the population genetic analysis align broadly with current trends in malaria transmission and disease burden. Lower F_WS_ values were estimated for isolates from South Asia, West Africa, East Africa and Central Africa. This corresponds to the high transmission intensity and frequent outcrossing known to occur in these regions and recent global health reports^39,73,74^. Country level analysis showeded lower F_WS_ values in Mali and Kenya in recent years (2016 onwards), suggesting cases from these countries should be of key concern and targets for malaria intervention. Conversely, low F_WS_ was reported in the Horn of Africa, South America, Oceania and Southeast Asia, which could indicate less outbreeding and lower transmission because of more effective malaria control measures. However, this may also be influenced by the smaller sample sizes from some of these regions. Specific countries might also exhibit low F_WS_ due to geographic isolation, as previously observed in samples from the Bijagós islands^33^. These findings suggest that *P. falciparum* infections in South Asia and Southeast Asia should be considered independently during global assessments rather than being combined. This contrasts with observations in *P. vivax*, where F_WS_ values are marginally higher in South Asia than in Southeast Asia, yet remain broadly similar overall, likely reflecting the role of relapse in maintaining genetic diversity across regions with comparable transmission intensity^75^.

An important limitation is that F_WS_ estimates depend strongly on the sampling frame. For example, clinical or hospital-based sampling tends to capture symptomatic, higher-parasitaemia infections, whereas active community sampling may capture a broader spectrum of infections, including low-density or asymptomatic cases. As a result, observed changes in F_WS_ over time or between regions should be interpreted cautiously, and primarily as indicators of broad-scale patterns of within-host heterozygosity relative to population diversity, rather than precise measures of local transmission intensity. Despite these limitations, integrating diverse datasets provides valuable context for understanding global patterns of parasite diversity. The F_WS_ results are also consistent with IBD analysis, where higher fractions were observed in the Horn of Africa, South America, Oceania and Southeast Asia, indicative of reduced outcrossing and lower transmission. High IBD fractions were particularly noted in genes involved in drug resistance. This observation may be due to strong positive selection from intensive antimalarial drug administration. Under such selective pressure, IBD sharing at drug-resistance loci may increase, alongside neutral genomic regions linked to these genes^51^. This effect could explain the higher IBD fractions found near *pfcrt*, which contains chloroquine-resistance mutations. High fractions of IBD were observed in *pfkelch13 *interaction candidates *KIC7* and *KIC9* across the Horn of Africa and South America, regions that may be affected by artemisinin resistance^76^. This result could indicate selection in these genes, which should consequently be a target for future investigation into markers for artemisinin-resistance. In addition, it has been suggested that regions under strong positive selection can bias IBD analysis^51^. Further research is needed to mitigate this effect for downstream IBD-based inference, such as estimating effective population size.

Moreover, the impact of positive selection was evident from composite selection analyses, which integrate multiple signals of positive selection to identify robust candidate selective sweeps while reducing false positives. Genome-wide scans using extended haplotype homozygosity within populations (iHS) and between populations (XP-EHH) identified genomic regions under strong selective pressure. These signals were further corroborated by Tajima’s D analyses, which showed an excess of low-frequency variants consistent with recent selective sweeps, as well as by elevated population differentiation detected through F_ST_ analysis. Across all geographical regions, the number and strength of genomic signals undergoing positive selection varied. Beyond drug resistance, selection targets genes critical for host-parasite interactions and fundamental parasite biology. Genes mediating erythrocyte invasion (*RON2, TREP*) and surface proteins (*PSSP17/PSOP17*) showed evidence of selection in multiple regions, reflecting adaptation to host immune pressure and vector ecology. Similarly, genes involved in transcriptional regulation (*TAF10, BDP1, Myb2*), RNA processing (*BUD31, SF3B2, POP1, POP4*), DNA replication (*ORC2, MCM3, MCM7*), and metabolic/stress pathways (*SufA, PTPS, HAD1, HSP60, PPPK-DHPS, HSP90*) also displayed concordant signals across multiple selection metrics. Combined, these results demonstrate that selection in *P. falciparum* may act not only on protein-coding changes but also on regulatory and epigenetic mechanisms, which should be explored further^77,78^.

Our composite selection analyses further identified distinct geographic patterns of selection and population differentiation in drug resistance candidate genes. The *pfdhps* gene (*PF3D7_0810800*), associated with sulfadoxine resistance, exhibited moderate signals of selection across East and West Africa and weaker signals elsewhere, consistent with its long-standing role in African SP treatment programmes^32,49,56,79^. In contrast, *pfkelch13* (*PF3D7_1343700*), the primary candidate for partial artemisinin resistance, displayed moderate selection signals restricted to South and Southeast Asia, reflecting the historical confinement of partial artemisinin resistance to these regions^42,67^. Other loci implicated in multidrug resistance or the Kelch13 pathway, including the AP–2 complex subunit mu (*PF3D7_1218300*; Kelch13 pathway), acetyl–CoA transporter (*PF3D7_1036800*), and elongation factor eEF2 (*PF3D7_1451100*), showed weaker but detectable selection in African and South Asian populations, which could suggest early adaptive responses in emerging resistance pathways. Although antimalarials specifically involving acetyl–CoA transporter (*PF3D7_1036800*, *PfACT*) and translation elongation factor eEF2 (*PF3D7_1451100*) are not in routine clinical use, both genes have been implicated in resistance pathways during experimental drug selection. In vitro evolution studies with imidazolopiperazines have shown that mutations in *PfACT* can confer cross‑resistance, implicating *PfACT* in broad multidrug resistance mechanisms^80^. Similarly, translation elongation factor eEF2 has been validated as the molecular target of the antimalarial cabamiquine/M5717, where selection of resistant parasites in laboratory settings arises through mutations affecting this essential protein synthesis factor^81^. Pre‑existing variation at these loci may therefore be subject to subtle selective pressures from current drug regimens or environmental factors. These genetic backgrounds could facilitate future resistance once drugs targeting these pathways are deployed.

After detecting the influence of anti-malarial treatment across *P. falciparum* genomes, we aimed to explore the patterns of drug-resistance mutations further. The geographic distribution of these mutations aligned with past and current drug administration programmes in each country and region. For instance, *pfkelch13* mutations were predominantly observed in samples from Southeast Asia and Oceania^17^. However, missense mutations were also observed in South America and South Central Africa, demonstrating the greater need for surveillance of *pfkelch13* markers outside of Southeast Asia^18^. Whilst a limited number of WGS from Africa included in the analysis have validated artemisinin-resistant genotypes, selection of these loci is important because they could provide a genomic context that enables the emergence or spread of resistance in the future. Additional markers of interest included *pfcrt* S350R detected in South America, further supporting the need for broad geographic sampling in genomic surveillance, ensuring that region-specific resistance markers are identified and monitored. Profiling drug-resistant strains could also be improved by examining structural variants and copy number variants that were not covered in this study^26^. Using a convenience sample approach is beneficial for examining large-scale drug resistance data, but caution is necessary when interpreting results due to temporal changes that may occur. Equally, identifying variations in candidate drug resistance among new drug candidates is important for anticipating potential resistance mechanisms that may emerge in the future.

Previous studies have also indicated that there may be compensatory mechanisms driving the evolution of artemisinin resistance across Southeast Asia^43,82^. We highlight the clonal expansion of *pfkelch13* C580Y mutants using IBD analysis, while also showing that other mutations have independently arisen from similar genomic backgrounds. In the absence of phenotypic data, potential interactors with the most common *pfkelch13* mutation (C580Y) were investigated by leveraging the power of the large-scale genome-wide sequencing data to search for SNP linkage, co-occurrence and association. Some mutations may be ‘background mutations’ associated with the genetic lineage of samples from Southeast Asia, which could be clarified through genome-wide association tests with the artemisinin drug susceptibility phenotype and the inclusion of population structure covariates^83^. Despite this, integrating our findings with evidence from published protein–protein interaction studies enabled the shortlisting of several strong candidate genes, including *pfcrt* and *ARPS10*, both of which have been previously reported, as well as *pfubp1*, which in this study exhibited signatures of positive selection in South Asia, West Africa, and East Africa^43^. Additional candidate genes include *MyoF* and *RAD5*. *MyoF* is associated with the Kelch13 compartment, while *RAD5* has also been reported to be under selection in the Greater Mekong Subregion, where the KEL1/PLA1 lineage is known to predominate^50,84^. This study also presents novel candidates associated with the *pfkelch13* C580Y genotype, including *PTEX150*, *PF3D7_0522400* (unknown function) and *PF3D7_1223500*, as well as *PF3D7_0826100*, which was detected via composite selection analysis only. *PTEX150* has been shown to facilitate trafficking of haemoglobin proteases, such as falcipain 2a and plasmepsin II, from the parasite into the parasitophorous vacuole and cytostome^85^. Kelch13-mediated resistance is thought to involve reduced haemoglobin uptake, limiting activation of artemisinin via haem^86^. Here, *PTEX150* may act as a compensatory mechanism, maintaining nutrient acquisition and parasite fitness, which could explain the observed selection signals. Existing haplotype combinations at *PTEX150* may have also facilitated the emergence of resistance, although functional validation is needed to confirm this role. The selection signal detected at *PTEX150* may also be influenced by linked selection, given its proximity to MCA-2 within the Kelch13 compartment, where elevated IBD could reflect selective processes related to artemisinin resistance^84^. *PF3D7_1223500* has an unknown function, but is situated close to the *GCH1* locus (GTP-cyclohydrolase 1), a region frequently involved in copy number variations associated with SP resistance. This further suggests that a drug-resistant genetic background is associated with the evolution of partial artemisinin resistance^87^. Although not associated with the C580Y genotype, *PF3D7_0826100* (HEUL) is of interest due to its role in the ubiquitin-proteasome system, with mutations in this gene previously reported in India^88^. While further validation is needed, the identification of emerging candidate mutations provides valuable insights into the potential drivers of artemisinin resistance that may emerge in other regions globally. The majority of isolates in the curated dataset were collected before the significant emergence of African *pfkelch13* propeller domain variants, which were first reported around 2020^17,55^. As such, further analysis of these variants is limited in this dataset and represents an important area for future research.

Although this global dataset and analysis encompass a wide geographic range, several regions of high epidemiological importance remain under-sampled and should be prioritised in future genomic studies. India is a key example, as *P. falciparum* populations from this region have proven highly informative for reconstructing parasite evolutionary history. Previous studies have shown that Indian parasites harbour exceptionally high mitochondrial genetic diversity, often exceeding that observed in many African populations, supporting the notion that this region may retain signatures of deep ancestral histories^89^. In addition, a unique lineage circulating in India has been proposed as a potential intermediate in the historical host transition of *P. falciparum* from non-human primates to humans^90^. The detection of *P. falciparum* in rhesus and bonnet macaques has also raised the possibility that a primate reservoir may contribute to local parasite ecology^91^. Likewise, increasing representation from the Horn of Africa, including countries such as Ethiopia, where artemisinin-resistant *pfkelch13* variants and *hrp2/hrp3* deletions are increasing in frequency^92,93^. Such variants affect ACT efficacy and accuracy of rapid diagnostic tests, respectively and are important to uncover. Whilst our work has included new samples from Brazil and historically under-represented regions in South America, broadening sampling in these areas will help capture the full extent of global *P. falciparum* diversity and strengthen population-genetic and surveillance inferences in the future. We also included 18 new WGS from Vietnam, all carrying the C580Y *pfkelch13* mutation. IBD analysis revealed elevated IBD fractions in the genomic region containing *KIC3*, a member of the Kelch13 compartment^84^. While this is consistent with a potential role for *KIC3* in facilitating artemisinin resistance, functional validation is needed to confirm its contribution, and the inclusion of recent samples provides an opportunity to further investigate this candidate locus.

Overall, this study identified distinct global patterns of genetic variation in *P. falciparum* that vary over time and across countries and regions. When integrated with further validation and complementary investigations, these insights can help to direct genomic surveillance toward timely interventions, with particular emphasis on monitoring drug resistance mutations, adaptation to *Anopheles* vectors, and geographic classification^20^.

## Methods

### Sequence data and pre-processing raw reads

A combined dataset of 23,462 *P. falciparum* isolates with WGS data was assembled for analysis. This included 16,203 high-quality samples that were obtained from the MalariaGen Pf7 data resource^26^, 7183 previously published samples^32–36,38,47,51,94,95^, and 76 isolates that were newly sequenced as part of this study (Brazil Amazon region (*n* = 58); Krông Pa, Khánh Hòa, and ChưR’Căm provinces, Vietnam (*n* = 18)), which were processed for this study using standard methods^32^. All participants provided informed consent. Ethical approval for the newly sequenced Vietnamese samples was obtained from the Research Ethics Committee of the National Institute of Malariology, Parasitology, and Entomology, Ministry of Health, Vietnam (Approval No. 1096/QĐ-VSR). This study was also approved by the Ethics Committee of the University of São Paulo (CAAE: 32707720.0.0000.5467). After pre-processing the raw genomic data, duplicate samples, mixed species and low-quality samples were removed from the dataset, leaving 17,565 high-quality samples for further analysis (2717 not included in Pf7) (Supplementary Data 20). Raw paired-end sequence data were processed using an established bioinformatic pipeline (https://github.com/LSHTMPathogenSeqLab/fastq2matrix). FastQ files were obtained from the European Nucleotide Archive and were trimmed to remove poor quality sequences using trimmomatic (v0.39) and the following parameters: LEADING:3 TRAILING:3 SLIDINGWINDOW:4:20 MINLEN:36^96^. Trimmed sequences were subsequently aligned to the *P. falciparum* 3D7 reference genome (v3) (GCA_000002765.3) using bwa-mem (v0.7.17-r1188) to produce BAM files^97^. Samtools (v1.18) (fixmate and markdup) was used to correct mate information after mapping with bwa-mem^98^. Base quality score recalibration (BQSR) and correction were then performed using GATK (v4.1.4.1) (BaseRecalibrator and ApplyBQSR) to reduce systematic errors in the quality score of base calls derived from the sequencing process^99^. This was carried out using the *P. falciparum* genetic crosses 1.0 dataset (https://www.malariagen.net/data_package/pf-crosses-1-0/).

Variant calling (SNPs and small indels) was performed using GATK (v4.1.4.1) software (HaplotypeCaller) to create per-sample gVCF files (parameters: -ERC GVCF)^100^. The GenomicsDB datastore was used to store validated VCFs via the GATK’s GenomicsDBImport function. A multi-sample VCF file was then created using the GenotypeGVCFs function, and further quality score recalibration was carried out using the GATK Variant Quality Score Recalibration (VQSR) function (parameters: -an QD -an FS -an SOR -an DP -maxGaussians 8 and -mq-cap-for-logit-jitter-transform 70). Variant Quality Score Log-Odds (VQSLOD) were obtained using the ApplyVQSR function (parameter: -truth-sensitivity-filter-level 99.0, *P. falciparum* genetic crosses 1.0 dataset) and variants with VQSLOD score < 0 were filtered out to retain high-quality variant calls. Only SNPs found within the core genome were retained in the dataset. Further quality metrics were used to ensure the remaining dataset was of high quality. Isolates with >40% missing data were removed, leaving high-quality isolates for population genetic analysis that had consistent coverage across the core genome.

Alternative alleles were called only when they had ≥80% read support; sites below this threshold were classified as mixed. Variants were annotated using snpEff (v5.1) and bcftools csq tools^101,102^. After all filtering steps, 17,565 high-quality samples remained for further analysis. The high-quality multi-sample VCF was filtered for bi-allelic SNPs. Two VCF files were generated: one containing only bi-allelic SNPs, and a second containing normalised multi-allelic sites, where only the alternative allele with the highest minor allele frequency (MAF) was retained. The normalised VCF underwent additional filtering to remove additional hypervariable genes, which were defined as being in the 95% percentile for SNP density (SNPs per bp), and included multi-gene families known to be vulnerable to hypervariable sites (Supplementary Data 21).

### Population genetic analysis

Multidimensional scaling (MDS) was carried out using all samples and SNPs. A distance matrix was first calculated using PLINK (v1.90) software using a filtered, bi-allelic VCF file^103^. The MDS was carried out over the distance matrix using R (v4.2.2). F_ST_ analysis and estimation of SNP diversity (π) were carried out using the Python package scikit-allel (v1.37) (https://github.com/cggh/scikit-allel). The VCF file was converted to a ZARR format, and F_ST_ analysis was performed between regions, as well as a ‘one-against-all’ approach whereby samples from each region were compared to the rest of the dataset in the analysis. F_ST_ analysis was only carried out using segregating sites. Thresholds of F_ST_ > 0.75 and F_ST_ > 0.95 were used to identify sites with high F_ST_. Correlation between genetic and geographic distances (F_ST_) were estimated using the Mantel test using the vegan package in R (https://github.com/vegandevs/vegan). Mean pairwise difference was used to estimate SNP diversity in 10 kb windows, excluding sites with >80% missingness and SNPs in non-coding regions. However, estimates derived from SNP diversity analyses are strongly influenced by missingness thresholds and the exclusion of invariant sites, and therefore should be interpreted with caution. All code for these analyses is available on GitHub in a dedicated repository: https://github.com/LSHTMPathogenSeqLab/Pop_Gen. F_WS_ was first estimated using only coding regions and bi-allelic SNPs. A population-specific MAF threshold of 1% was used to filter SNPs (threshold for all analyses unless stated otherwise). The multi-sample VCFs were split and filtered according to country and region using bcftools (v1.20), and the F_WS_ metric was calculated using the moimix R package (v0.0.2.9001) (https://github.com/bahlolab/moimix)^101^. An F_WS_ threshold of >0.95 was applied to exclude multiclonal isolates from selection, IBD, and drug-resistance haplotype analyses. F_WS_ scores were compared across countries and sampling years using ANOVA to identify temporal and geographic variation in F_WS_ values. Year groups were defined on a distribution basis and spanned 3-year periods. P-values were adjusted using the false discovery rate, and *P* < 0.05 was used to indicate a significant difference between groups. Downstream analyses were performed using biallelic SNPs only, which were encoded in a binary genotype matrix (reference allele = 0, alternative allele = 1, mixed allele = 0.5, missing allele = Ns). ADMIXTURE software (v1.3) (parameters: -cv = 10 -j8 --haploid = “*”) was used to estimate individual ancestries across SNP genotypes^104^. This was performed using a bed file, which was converted from the multi-sample biallelic VCF file using PLINK (v1.90). Ten-fold cross-validation was used to estimate the optimal K value by inspecting the inflection point across models run with K values ranging from 1 to 12. The optimal value was *K* = 10 (Supplementary Fig. 24).

Furthermore, IBD sharing across genomes was estimated using hmmIBD software, which utilised a hidden Markov model^105^. The pairwise fraction collected for all specified regions and countries was used to estimate genomic relatedness. Genome-wide IBD fractions across 10 kb sliding windows were also calculated. Regions were annotated with gene annotations and gene products for enhanced interpretation. Chromosome painting was performed using JCVI^106^. Analysis of IBD at the country and year group level was also performed using isoRelate, and the iR statistic was used to identify sites undergoing positive selection^107^. The biallelic genotype matrix was also used to identify regions under positive selection using within-population iHS (threshold: −log10[1−2|Φ(iHS)-0.5| ] > 2.0) and between-population XP-EHH (threshold: 2) metrics, as implemented in the rehh R package^108^. Highly variable regions, such as *var* genes, were removed from the analysis to prevent false-positive results. In the second stage of analysis, genomic regions in the top 10% of Tajima’s D values were removed to reduce the inflation of selection metrics due to balancing selection. iHS is a within-population statistic in which positive values indicate selection favouring the ancestral allele, whereas negative values indicate selection favouring the derived allele. For XP-EHH, the sign of the statistic indicates the population in which selection is occurring. Genomic regions showing evidence of positive selection were further annotated using gene annotations and predicted gene products. Countries represented by fewer than five samples were excluded from population genetic analyses where applicable. Tajima’s D was calculated using VCFtools (v0.1.16) in 10-kb sliding windows. For each geographic region, the 10th and 90th percentiles of Tajima’s D were used to identify genomic regions potentially under balancing selection (upper 90th percentile) or enriched for rare alleles (lower 10th percentile). Genes of interest identified across regional level F_ST_ analyses (single and paired), IBD analysis, iHS and XPEHH, were annotated using GO terms (biological process, molecular function and cellular components) and KEGG pathways using PANTHER^109^. All selection analyses were also run using the normalised VCF file and binary matrix outputs. This was to prevent the exclusion of any important SNPs, including *pfkelch13* C580Y, in selection analyses. However, this did not have a large overall impact on the overall results, except for the inclusion of additional variation using the normalised multi-allelic VCF. The final reported results used the bi-allelic VCF, which had hypervariable regions removed (Supplementary Data 21).

To robustly identify genomic regions under recent positive selection, we combined four complementary statistics - Tajima’s D, iHS, XP-EHH, and IBD fractions - into a composite selection framework. Genomic windows were classified as showing significant evidence of selection if they fell within the lowest 10% of Tajima’s D, the highest 15% of IBD fractions, and exhibited significant iHS or XP-EHH signals. To characterise population differentiation, we identified SNPs with F_ST_ values > 0.2 and annotated moderately differentiated variants located within selected windows, linking them to known or putative functional genes. This integrative approach prioritised loci with concordant signals across multiple metrics, thereby reducing false positives and highlighting genes likely subject to positive selection. Genomic regions were categorised according to the number of supporting metrics: weak (at least two metrics), moderate (three metrics), and strong (four metrics) evidence of selection. MAF thresholds for regional iHS and XP-EHH analyses were determined by sample size. Regions with more than 1000 samples used a MAF threshold of 0.01 (West Africa, Southeast Asia, South Asia, South America, and East Africa), whereas a threshold of 0.05 was applied to all other regions. Samples from the Horn of Africa were excluded from iHS and XP-EHH analyses due to insufficient sample size.

### Drug resistance mutations and *pfkelch13* C580Y associations

Drug resistance mutations were assessed using the multi-allelic VCF file. Known drug-resistance mutations were retrieved from the Malaria-Profiler database (https://github.com/jodyphelan/malaria-db) and WHO, which were used to filter the VCF files^20^. Additional nonsynonymous mutations were selected by filtering gene boundaries using a bed file found within the database. This was used to generate a matrix for identifying additional mutations and nonsynonymous mutation combinations. Indels were also included in the matrix (e.g., for *pfcrt* N75E). For haplotype analysis, only samples with sufficient coverage (depth>5) were used in the analysis. Haplotype combinations of missense SNPs (and Indels for *pfcrt* haplotypes) across drug resistance candidate genes were identified per region and visualised using heatmaps. Samples from Southeast Asia were subject to further IBD analysis. Outputs from hmmIBD were used to construct IBD networks across Southeast Asia, across three-year time periods spanning from 2008 to 2019. Networks were visualised using ipysigma (https://github.com/medialab/ipysigma). 47.5% and 95% IBD thresholds were used to reveal broader, ancestral connectivity and recent, direct clonal expansion. Pairwise IBD fractions were compared across three genotypic groups: *pfkelch13* C580Y, other missense mutations in the propeller domain, and wild-type. Statistical significance was assessed using the Kruskal-Wallis test for overall group differences, followed by the two-sided Wilcoxon Rank Sum Test for pairwise comparisons.

A multi-layered association analysis was performed to identify SNPs associated with the *pfkelch13* C580Y mutation using both a drug-resistance binary matrix and a genome-wide binary matrix restricted to common variants (MAF > 0.01). Analyses were conducted under two complementary statistical frameworks: (i) a logistic regression model adjusted for the top five principal components across all samples (“All”), and (ii) latent factor mixed models implemented within each country (Cambodia, Laos, Vietnam, Myanmar, and Thailand) using the LEA package to account for population structure and admixture, with the optimal number of latent factors (K) inferred^110^ using snmf. In both frameworks, P-values were corrected for multiple testing using the Benjamini-Hochberg procedure. To complement the association models, we calculated an interaction coefficient expressed as an odds ratio (OR), defined as OR = (A × D)/(B × C) based on the co-occurrence of SNP alleles with C580Y (zero counts were imputed as 0.5), along with genotypic correlations (*r²*) estimated using VCFtools (v0.1.16). Four metrics - adjusted P-values from the logistic regression, adjusted P-values from the latent factor models, OR, and r² - were integrated to prioritise SNPs into three confidence tiers. Tier 1 included SNPs supported by at least three of the four criteria (adjusted *P* < 0.001, *r²* > 0.5, OR  in top percentile). Tier 2 comprised SNPs meeting two criteria with moderate support (*r²* > 0.2 and OR > 5). Tier 3 included SNPs meeting a single criterion and located within a curated set of genes with known biological relevance or prior evidence of involvement (Supplementary Data 22)^84^. STRING protein-protein interaction analysis, clustered using the k-means algorithm, was used to identify functional links among candidate genes^111^.

### Reporting summary

Further information on research design is available in the Nature Portfolio Reporting Summary linked to this article.

## Supplementary information

Supplementary Information

Description of Additional Supplementary Files

Supplementary Dataset 1–22

Reporting Summary

Transparent Peer Review file

## Data Availability

Raw sequencing data are available in the European Nucleotide Archive (ENA). A complete list of accession numbers is provided in PRJEB94034. This publication also uses data from the MalariaGEN *Plasmodium falciparum* Community Project as described in ‘An open dataset of *Plasmodium falciparum* genome variation in 7000 worldwide samples. MalariaGEN et al. Wellcome Open Research 2021642 DOI: 10.12688/wellcomeopenres.16168.1. Source data are provided with this paper. Accessions for all samples used in this study can be found in Supplementary Data 20.

## Code availability

Code used for this analysis can be found in a dedicated GitHub repository: https://github.com/LSHTMPathogenSeqLab/Pop_Gen.
