# Supplementary material for: Global-scale population genetic analysis of Plasmodium falciparum identifies region-specific patterns of malaria parasite adaptation
Source: Nat Commun. 2026 May 11;17:6318. doi: 10.1038/s41467-026-73006-2 (PMC13377190; doi:10.1038/s41467-026-73006-2)
Supplement: Supplementary file 1 — Supplementary Information [file 41467_2026_73006_MOESM1_ESM.pdf]

Global-scale population genetic analysis of *Plasmodium falciparum* identifies region-specific patterns of malaria parasite adaptation

Nina Billows[1], Jamille G. Dombrowski[2], Joseph Thorpe[1], Leen Vanheer[1], Sophie Moss[1], Jesse Gitaka[4], Colin J. Sutherland[1,5], Claudio R. F. Marinho[2], Nguyen Thi Hong Ngoc [6], Nguyen Thi Huong Binh[6], Nguyen Quang Thieu[6], Susana Campino [1,*], Taane G. Clark [1,7,*]

[1] Faculty of Infectious and Tropical Diseases, London School of Hygiene and Tropical Medicine, London, United Kingdom

[2] Department of Parasitology, Institute of Biomedical Sciences, University of São Paulo, São Paulo, Brazil.

[3] School of Medicine, Instituto de Medicina Tropical, University of São Paulo, São Paulo, Brazil.

[4] Directorate of Research and Innovation, Mount Kenya University, Gen. Kago Rd, Thika, Kenya.

[5] UK Health Security Agency Malaria Reference Laboratory, LSHTM, London, WC1E 7HT, UK.

[6] Molecular Biology Department, Parasitology and Entomology, Vietnam National Institute of Malariology, Hanoi, Vietnam.

[7] Faculty of Epidemiology and Population Health, London School of Hygiene and Tropical Medicine, London, United Kingdom

SUPPLEMENTARY FIGURES

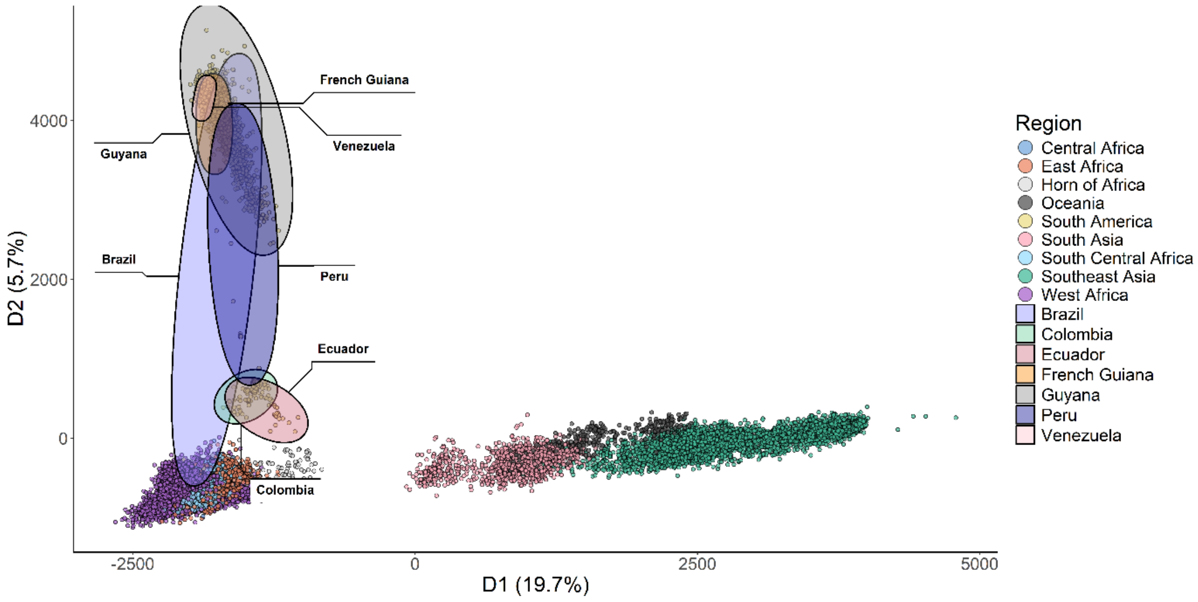

Supplementary Figure 1. Population structure of global *P. falciparum* samples showing separation amongst South American samples. Multidimensional scaling: D1 (19.7%) vs D2 (5.7%) are shown and overlap between samples from South American countries are highlighted and labelled. Source data are provided as a Source Data file.

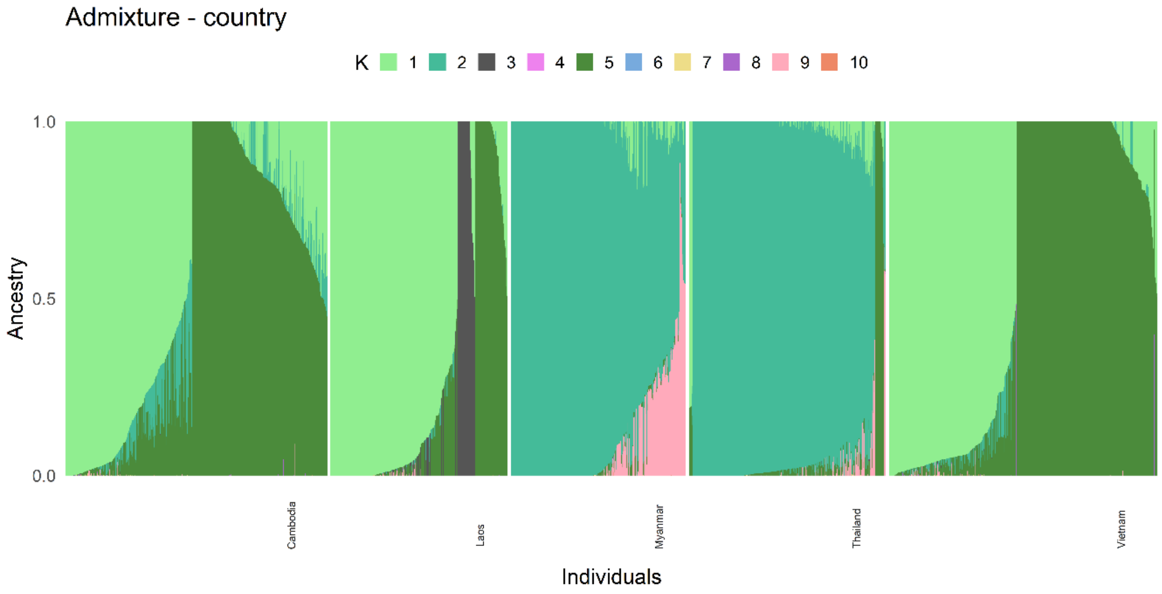

Supplementary Figure 2. Admixture within Southeast Asia. Samples underwent admixture analysis (K=10). Dominant clusters are shown for Southeast Asian samples, representing diverse ancestral populations. Source data are provided as a Source Data file.

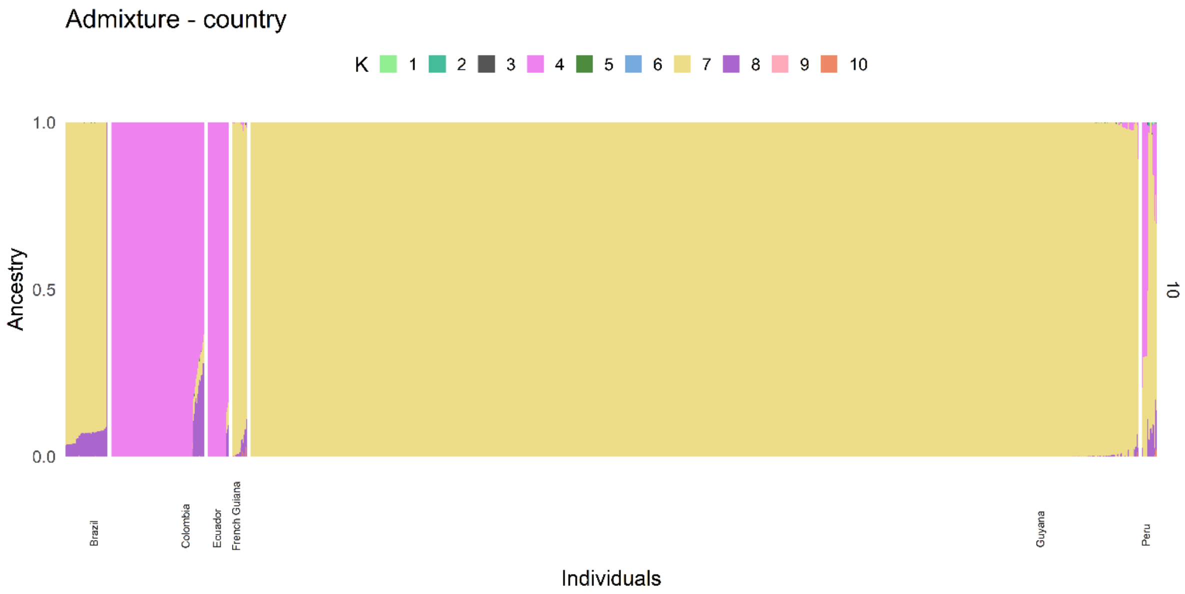

Supplementary Figure 3. Admixture within South America. Samples underwent admixture analysis (K=10). Dominant clusters are shown for South American samples, representing diverse ancestral populations with distinct clusters. Source data are provided as a Source Data file.

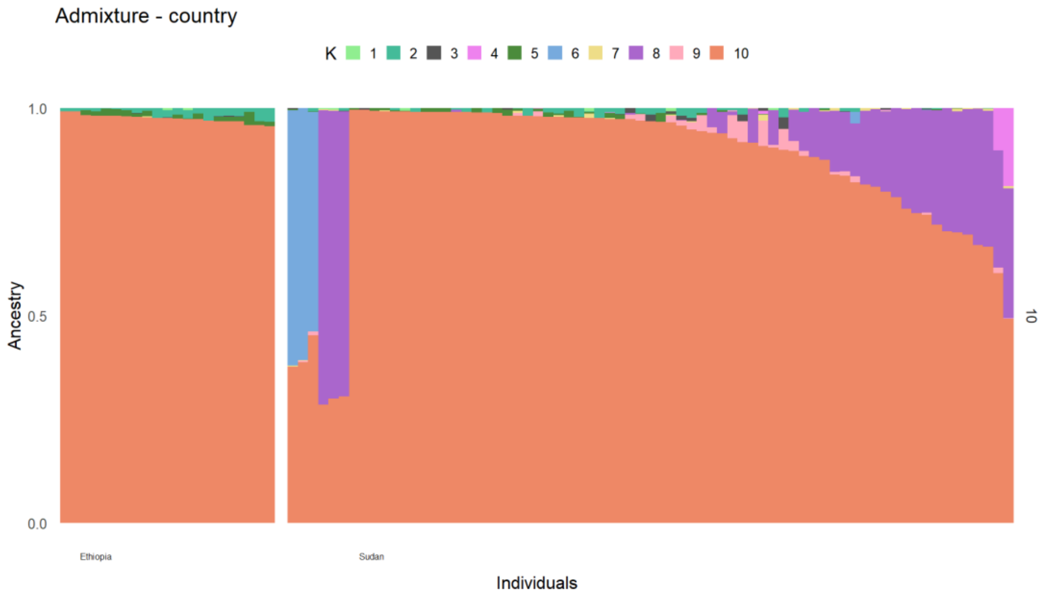

Supplementary Figure 4. Admixture within Horn of Africa. Samples underwent admixture analysis (K=10). Dominant clusters are shown for Horn of Africa samples, representing diverse ancestral populations with distinct clusters. Source data are provided as a Source Data file.

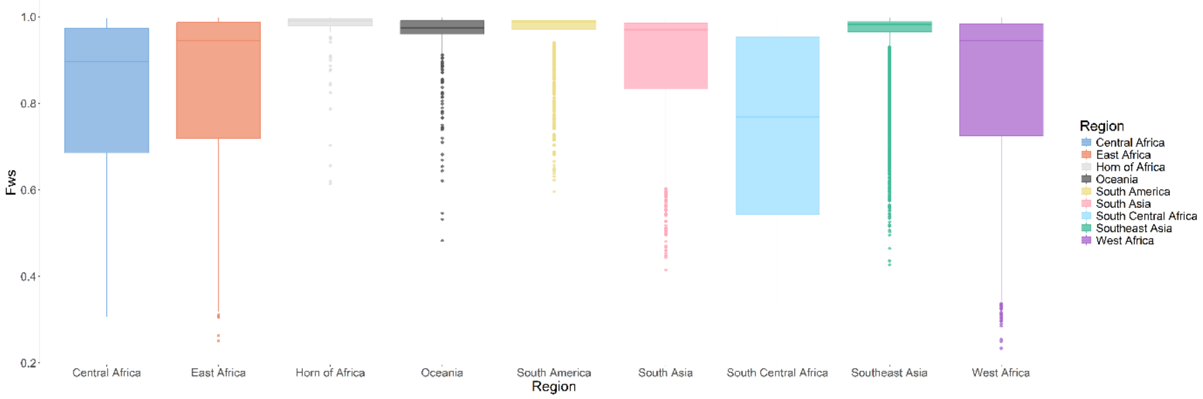

Supplementary Figure 5. Distribution of F_{WS} scores for *P. falciparum* per region. Boxplots showing the median, interquartile range and range of F_{WS} scores are highlighted per region. Source data are provided as a Source Data file.

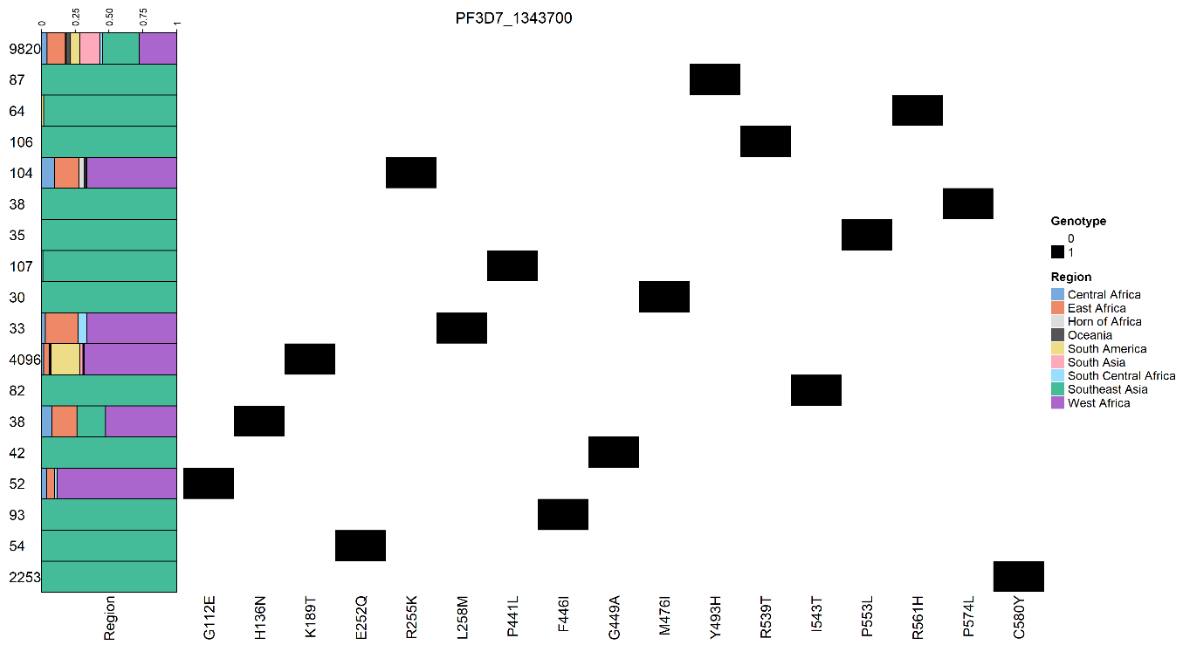

Supplementary Figure 6. Proportion and distribution of drug-resistant haplotypes for *pfkelch13* across the global *P. falciparum* dataset. Haplotypes in drug-resistant candidate genes were defined according to the Malaria-Profiler tool and WHO databases, and constructed using missense SNPs and indels, as well as samples with sufficient coverage (depth >5-fold), across each gene. The heatmap highlights the combination of genotypes reported for *pfkelch13*, the number of overall samples with each haplotype and the proportion from each geographic region. Each haplotype was observed in >10 samples to aid visualisation. Source data are provided as a Source Data file.

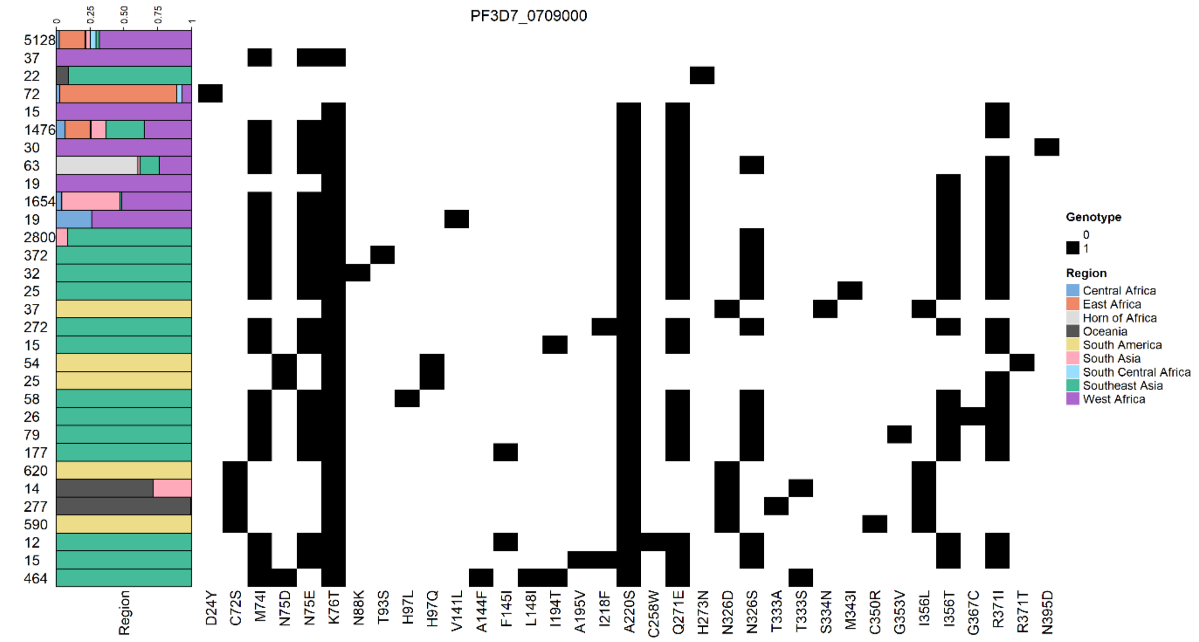

Supplementary Figure 7. Proportion and distribution of drug-resistant haplotypes for *pfcrt* across the global *P. falciparum* dataset. Haplotypes in drug-resistant candidate genes were defined according to the Malaria-Profiler tool and WHO databases, and constructed using missense SNPs and indels, as well as samples with sufficient coverage (depth >5-fold), across each gene. The heatmap highlights the combination of genotypes reported for *pfcrt*, the number of overall samples with each haplotype and the proportion from each geographic region. Each haplotype was observed in >10 samples to aid visualisation. Source data are provided as a Source Data file.

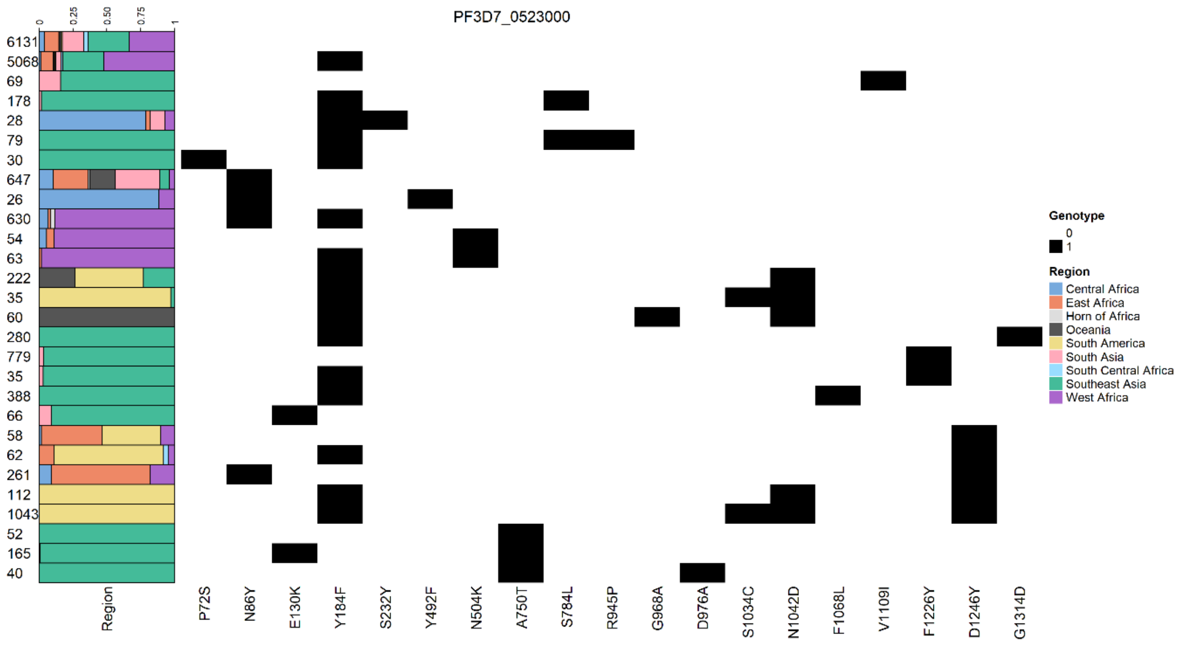

Supplementary Figure 8. Proportion and distribution of drug-resistant haplotypes for *pfmdr1* across the global *P. falciparum* dataset. Haplotypes in drug-resistant candidate genes were defined according to the Malaria-Profiler tool and WHO databases, and constructed using missense SNPs and indels, as well as samples with sufficient coverage (depth >5-fold), across each gene. The heatmap highlights the combination of genotypes reported for *pfmdr1*, the number of overall samples with each haplotype and the proportion from each geographic region. Each haplotype was observed in >10 samples to aid visualisation. Source data are provided as a Source Data file.

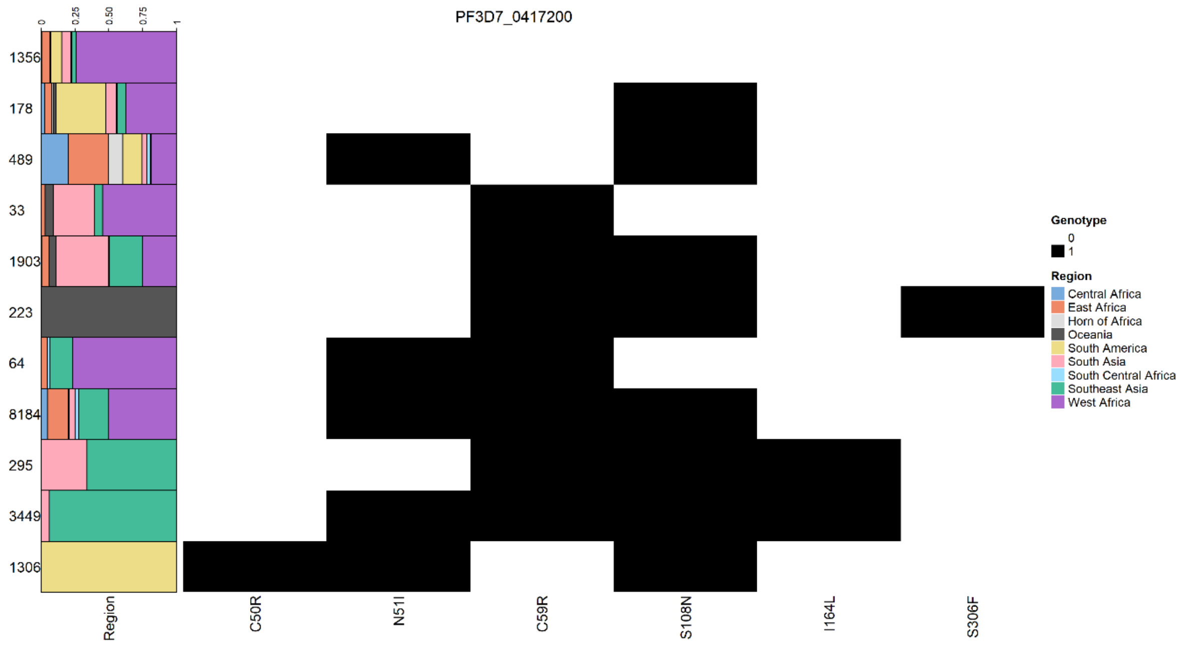

Supplementary Figure 9. Proportion and distribution of drug-resistant haplotypes for *pfdhfr* across the global *P. falciparum* dataset. Haplotypes in drug-resistant candidate genes were defined according to the Malaria-Profiler tool and WHO databases, and constructed using missense SNPs and indels, as well as samples with sufficient coverage (depth >5-fold), across each gene. The heatmap highlights the combination of genotypes reported for *pfdhfr*, the number of overall samples with each haplotype and the proportion from each geographic region. Each haplotype was observed in >10 samples to aid visualisation. Source data are provided as a Source Data file.

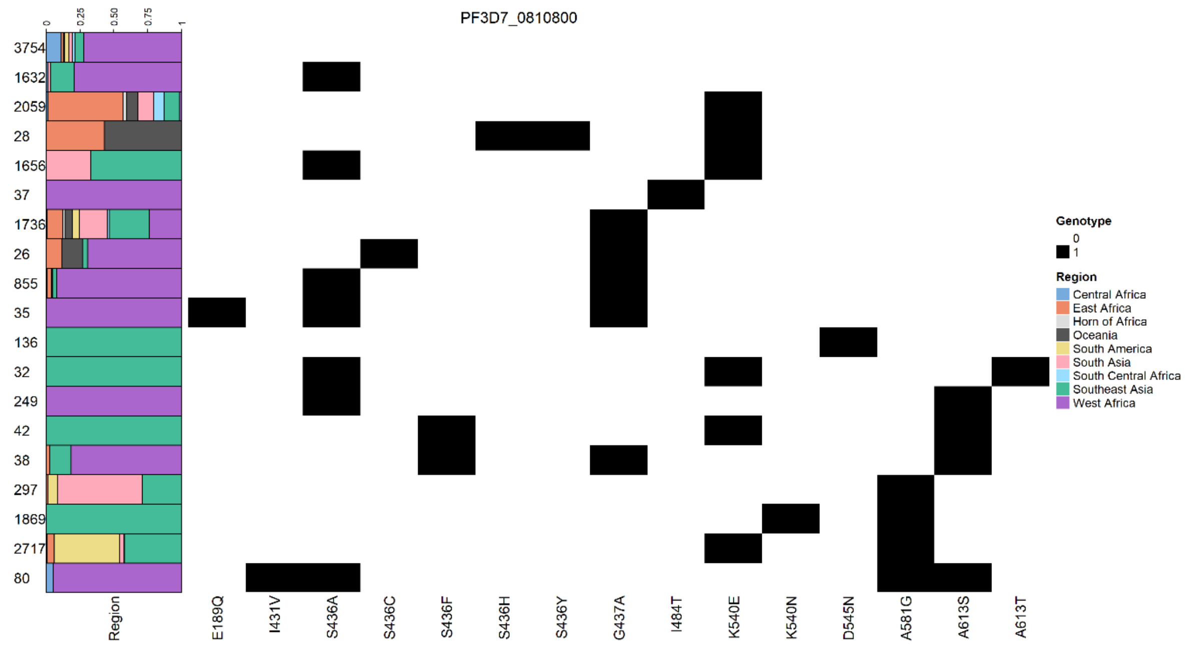

Supplementary Figure 10. Proportion and distribution of drug-resistant haplotypes for *pfdhps* across the global *P. falciparum* dataset. Haplotypes in drug-resistant candidate genes were defined according to the Malaria-Profiler tool and WHO databases, and constructed using missense SNPs and indels, as well as samples with sufficient coverage (depth >5), across each gene. The heatmap highlights the combination of genotypes reported for *pfdhps*, the number of overall samples with each haplotype and the proportion from each geographic region. Each haplotype was observed in >10 samples to aid visualisation. Source data are provided as a Source Data file.

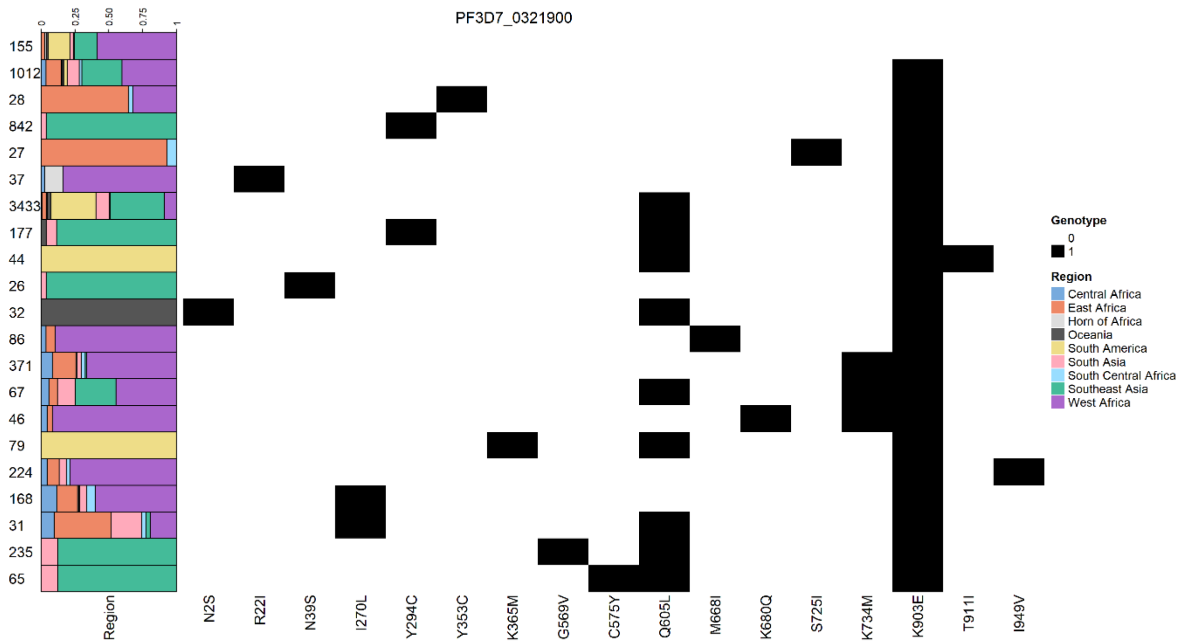

Supplementary Figure 11. Proportion and distribution of drug-resistant haplotypes for *pfCARL* across the global *P. falciparum* dataset. Haplotypes in drug-resistant candidate genes were defined according to the Malaria-Profiler tool and WHO databases, and constructed using missense SNPs and indels, as well as samples with sufficient coverage (depth >5-fold), across each gene. The heatmap highlights the combination of genotypes reported for *pfCARL*, the number of overall samples with each haplotype and the proportion from each geographic region. Each haplotype was observed in >10 samples to aid visualisation. Source data are provided as a Source Data file.

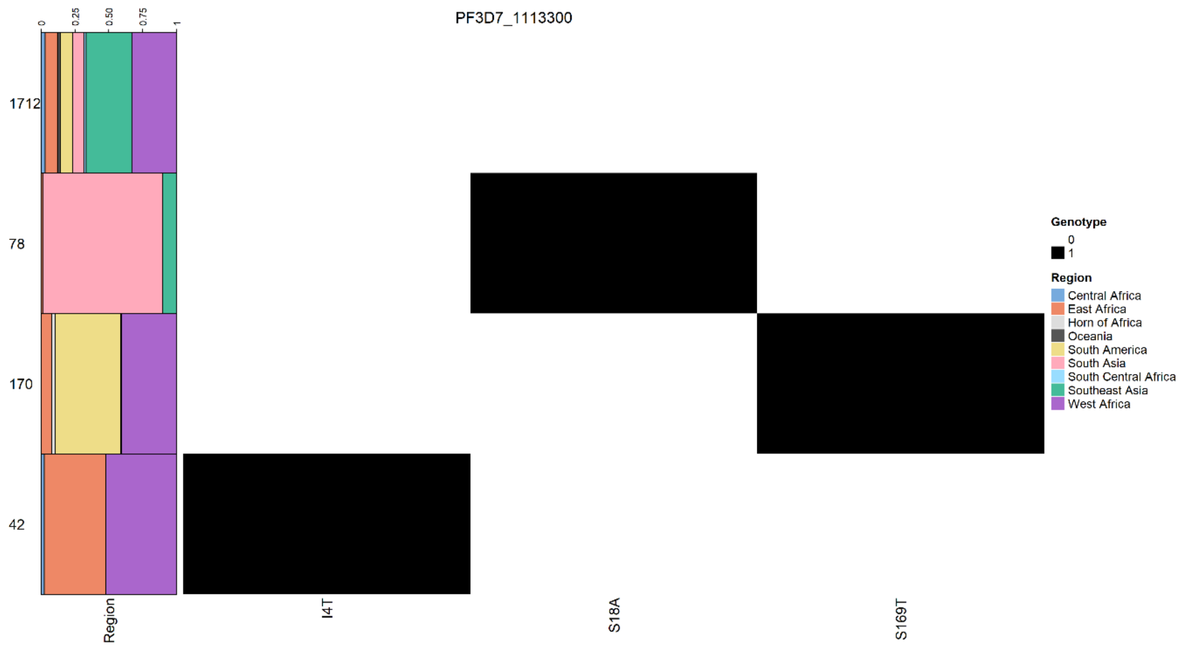

Supplementary Figure 12. Proportion and distribution of drug-resistant haplotypes for *PF3D7_1113300* (UDP-galactose transporter) across the global *P. falciparum* dataset. Haplotypes in drug-resistant candidate genes were defined according to the Malaria-Profiler tool and WHO databases, and constructed using missense SNPs and indels, as well as samples with sufficient coverage (depth >5-fold), across each gene. The heatmap highlights the combination of genotypes reported for *PF3D7_1113300*, the number of overall samples with each haplotype and the proportion from each geographic region. Each haplotype was observed in >10 samples to aid visualisation. Source data are provided as a Source Data file.

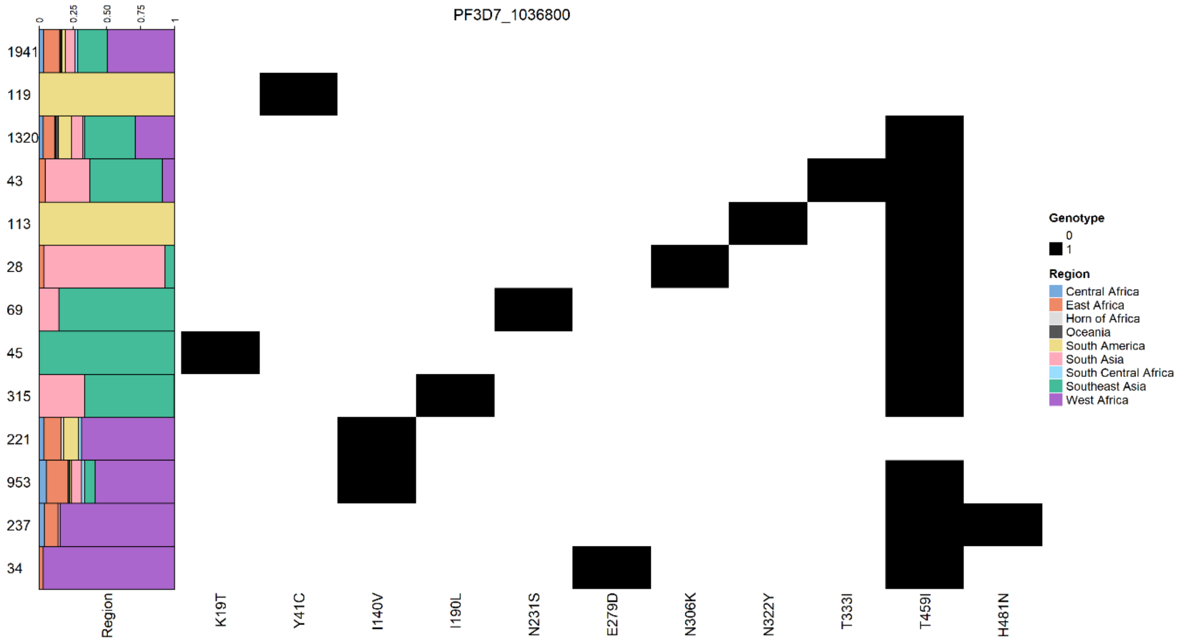

Supplementary Figure 13. Proportion and distribution of drug-resistant haplotypes for *PF3D7_1036800* (Acetyl-CoA-transporter) across the global *P. falciparum* dataset. Haplotypes in drug-resistant candidate genes were defined according to the Malaria-Profiler tool and WHO databases, and constructed using missense SNPs and indels, as well as samples with sufficient coverage (depth >5-fold), across each gene. The heatmap highlights the combination of genotypes reported for *PF3D7_1036800*, the number of overall samples with each haplotype and the proportion from each geographic region. Each haplotype was observed in >10 samples to aid visualisation. Source data are provided as a Source Data file.

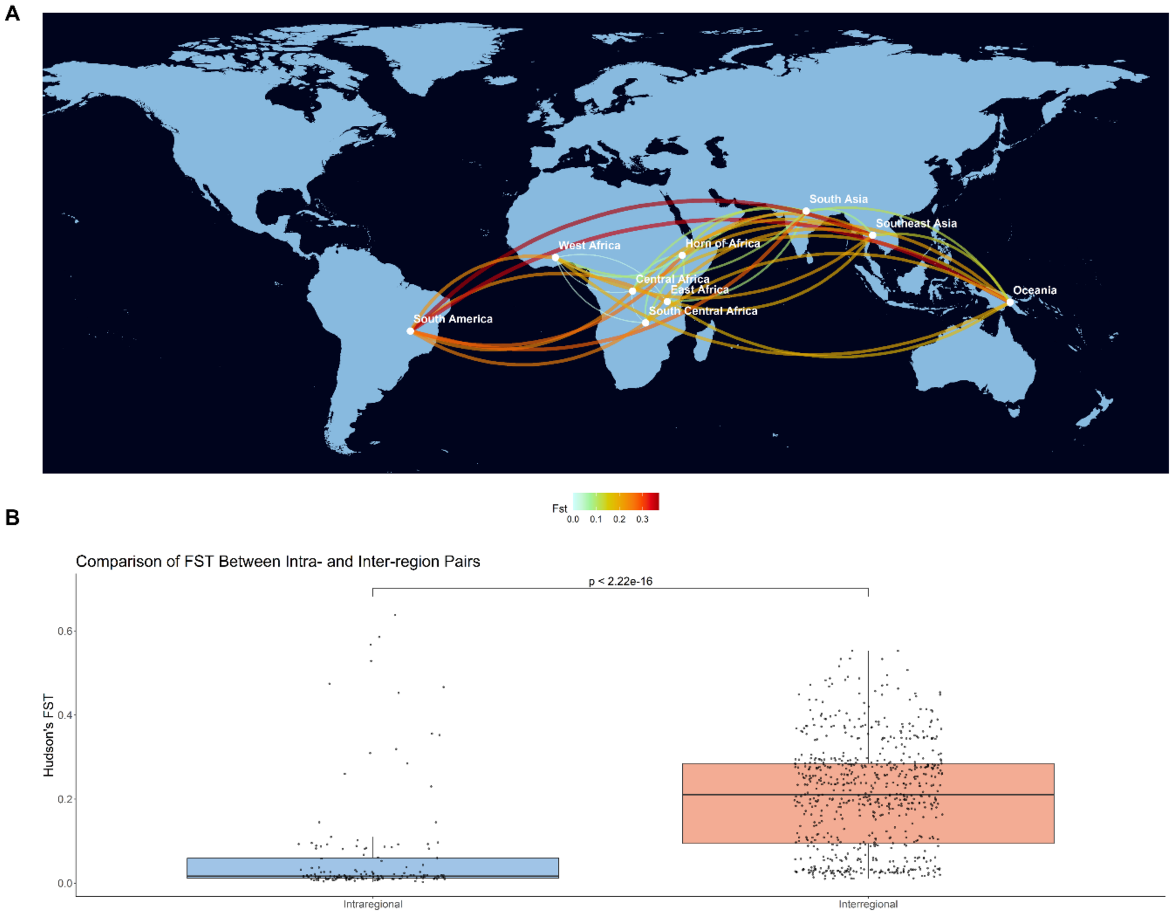

Supplementary Figure 14. Genetic differentiation (F_{ST}) is greater between intercontinental regions. A network map showing edges connecting regions weighted by the average difference in F_{ST} scores between regions **(A).** A box and whisker plot shows the distribution of average Hudson's F_{ST} values from intra-regional and inter-regional country comparisons **(B)**. A Wilcoxon rank-sum test (two-sided) confirms the difference in distributions between the two sets of comparisons. Source data are provided as a Source Data file. Map data has been produced using the 'maps' R package.

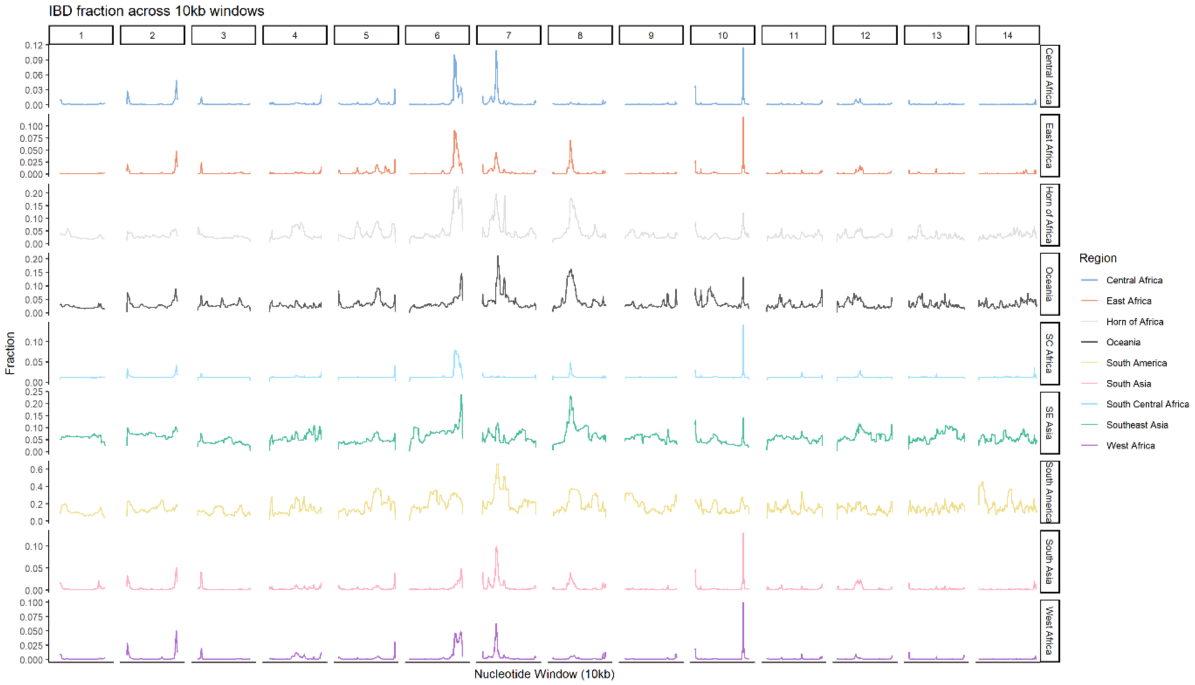

Supplementary Figure 15. Fraction of Identity by Descent (IBD) across 10kb windows. IBD fractions are shown across 14 nuclear chromosomes for 9 geographical regions. SC= South Central; SE= Southeast. Source data are provided as a Source Data file.

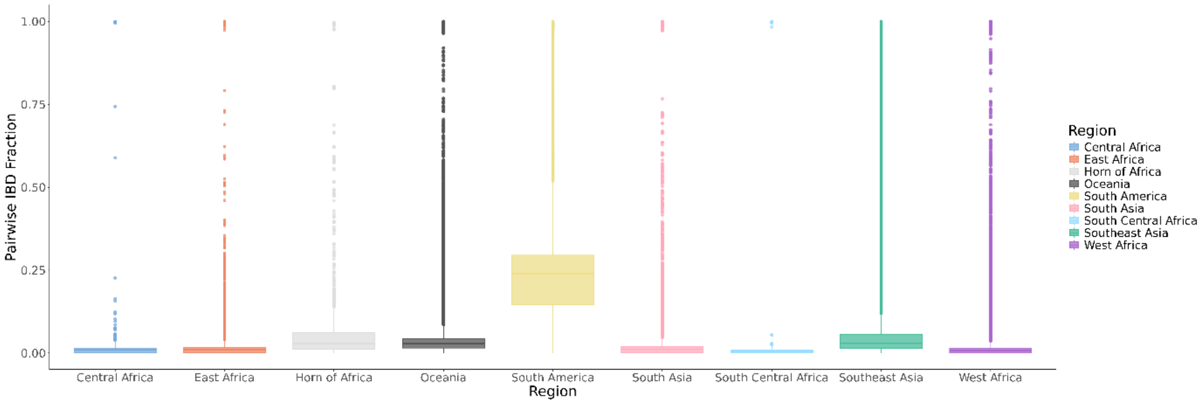

Supplementary Figure 16. Pairwise Fractions of Identity by Descent (IBD) across regions. Boxplots showing, the median and interquartile range of pairwise IBD fractions (sample-sample comparisons) are shown across 14 nuclear chromosomes for 9 geographical regions.

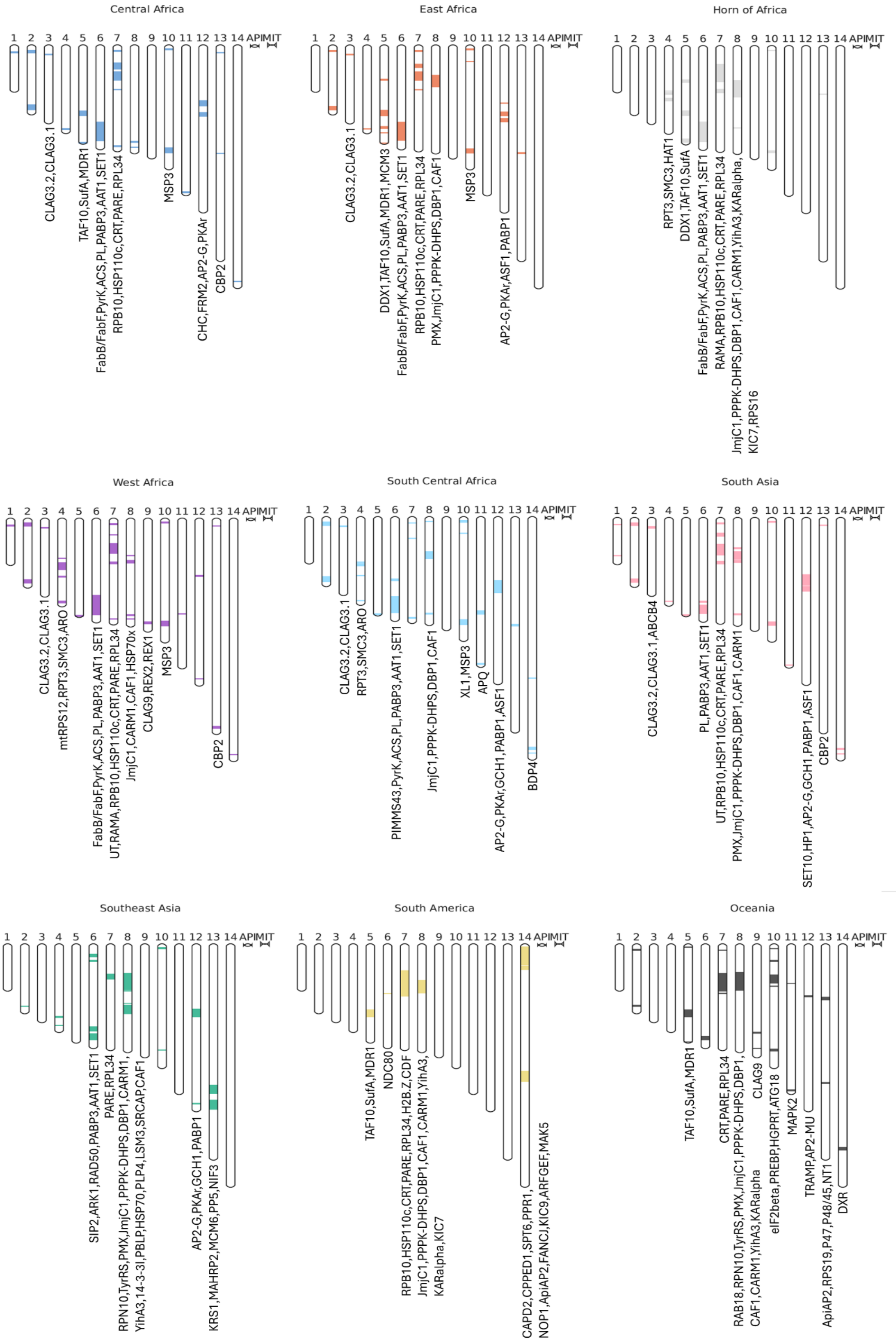

Supplementary Figure 17. Chromosomal regions with high fractions of Identity by Descent (IBD) for

each region. Chromosome painting of regions across 14 nuclear chromosomes with high IBD fractions (0.95 quantile) are shown for 9 regions. Genes within regions of high IBD are shown next to their respective chromosomes. Source data are provided as a Source Data file.

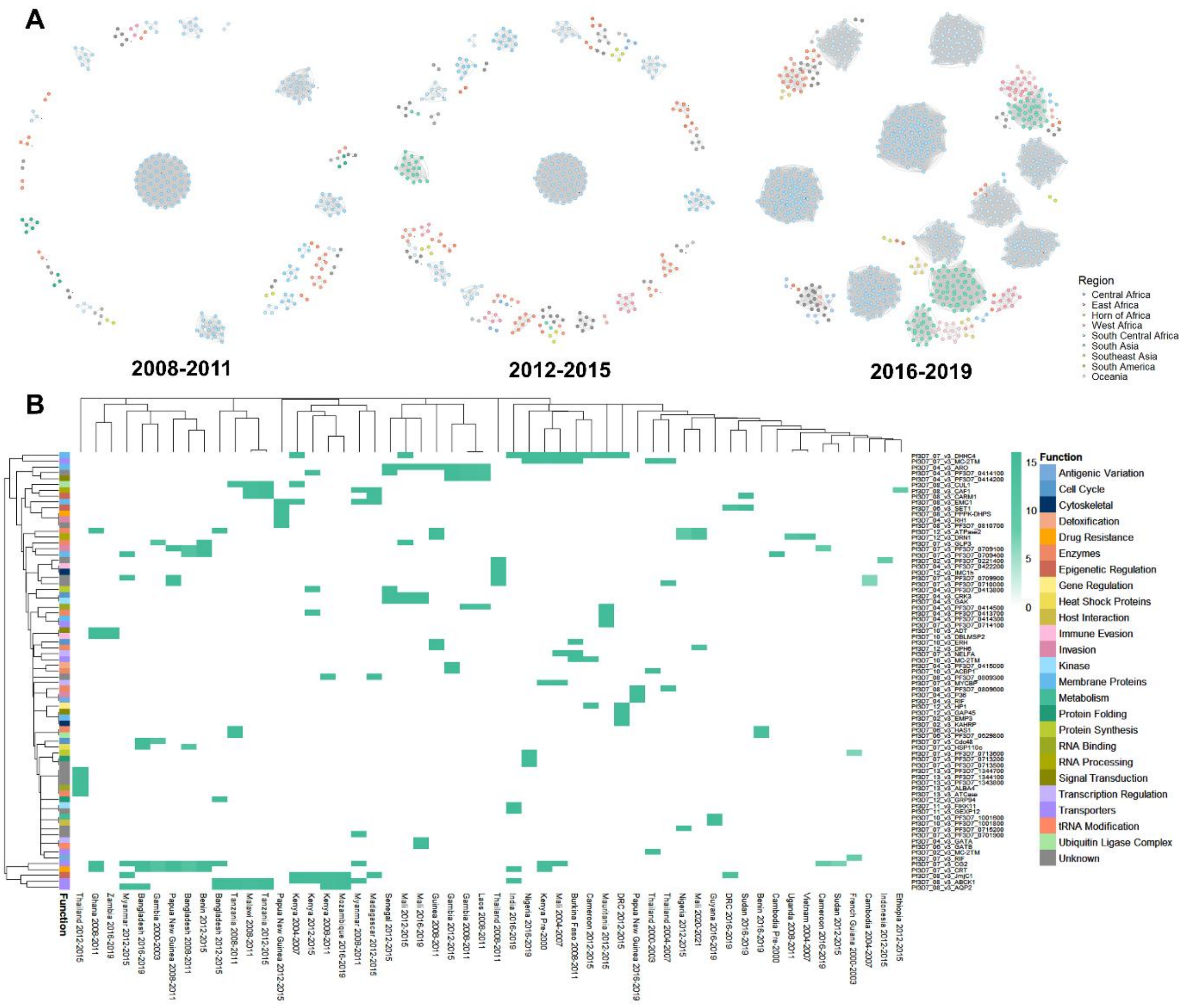

Supplementary Figure 18. Sample-level clustering based on IBD and loci under selection (iR) across countries and three-year time intervals. (A) Sample-level clustering based on identity-by-descent (IBD, proportion ≥ 0.95) across the three most common time intervals (2008–2011, 2012–2015, and 2016–2019), coloured by region. (B) Presence of loci under selection ('top hits') across countries and time intervals, with shade intensity reflecting $-\log_{10}$ P-values; 0 indicates no evidence of selection. Loci are annotated by their known or predicted function. Source data are provided as a Source Data file.

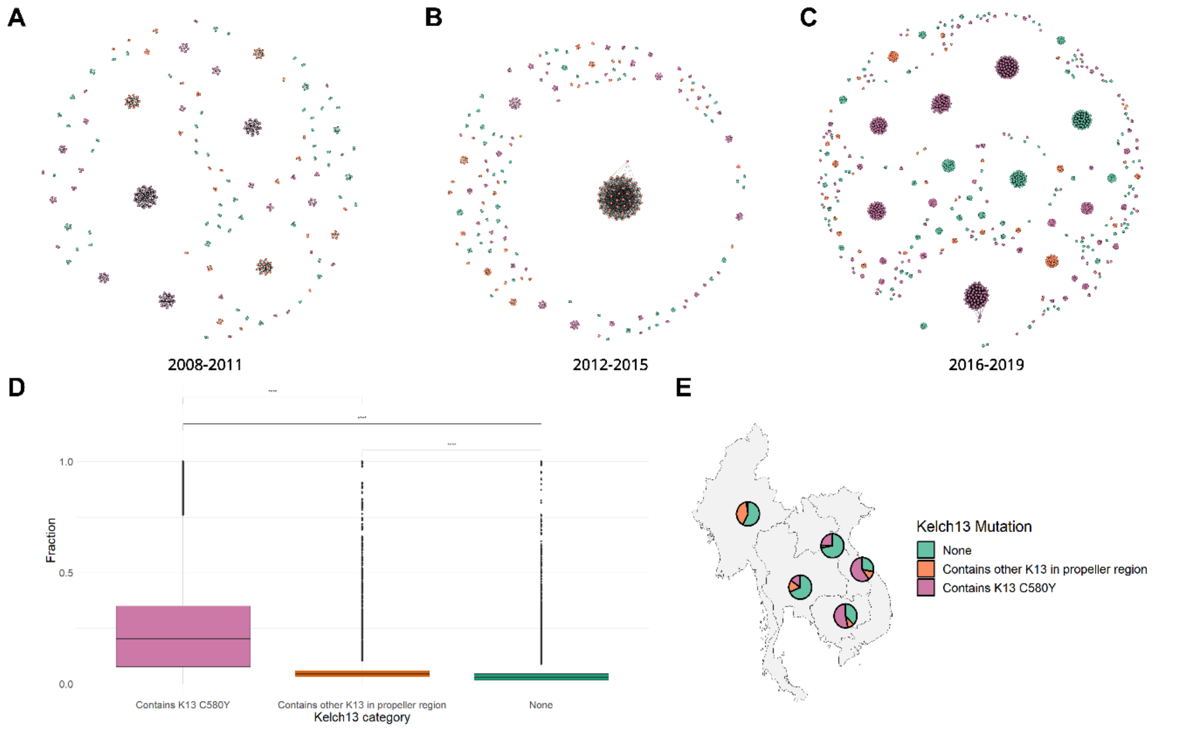

Supplementary Figure 19. IBD networks illustrating clonal expansion of *pfkelch13* mutant lineages from 2008 to 2019 across Southeast Asia. IBD networks were constructed using a 95% pairwise IBD threshold for three time periods: 2008–2011 **(A)**, 2012–2015 **(B)**, and 2016–2019 **(C)**. Nodes represent isolates and are coloured by their *pfkelch13* genotype: C580Y, other missense mutations in the propeller domain, or wild-type. Association between other K13 mutations in the propeller region (2012-2015) could be explained by strong intra-Vietnam relatedness (**Supplementary Figure 20**). Isolates with missing *pfkelch13* genotypes were excluded. A boxplot showing the distribution of pairwise IBD fractions among isolates grouped by their *pfkelch13* genotype: C580Y (N=1,858), other propeller domain mutations (N=756), and wild-type (N=2,001) **(D)**. A Wilcoxon rank-sum test confirms the difference in distributions between the IBD comparisons (*** indicates $P < 2 \times 10^{-16}$). A pie chart showing the proportion of *pfkelch13* genotypes (C580Y, other mutations, wild-type) by country (Laos: 671, Cambodia: 939, Vietnam: 1,016, Myanmar: 762, Thailand:694). The colour key from this panel is used consistently across the figure **(E)**. Source data are provided as a Source Data file (source_data_2).

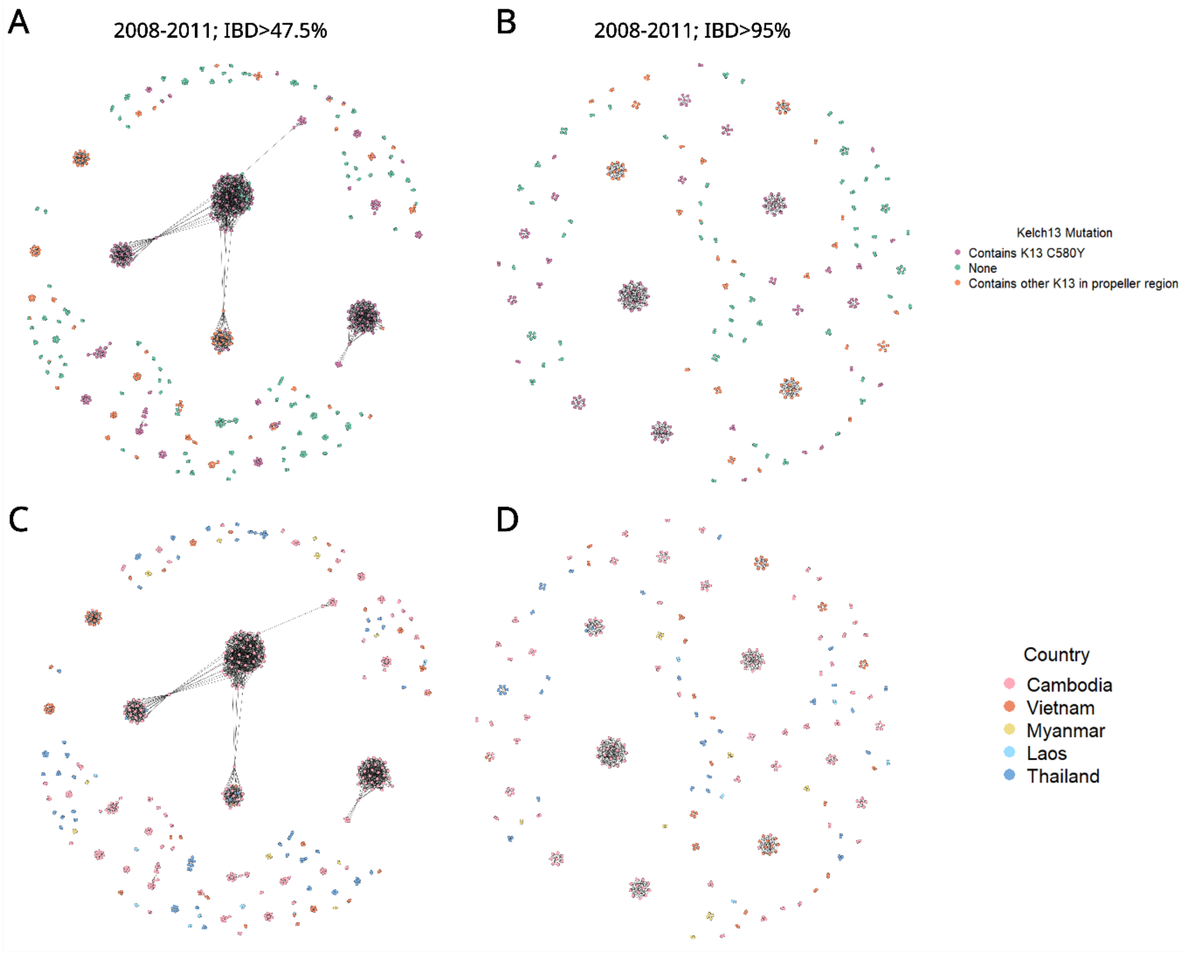

Supplementary Figure 20. Identity-by-descent (IBD) networks in Southeast Asia showing clusters at the 47.5% and 95% pairwise IBD thresholds (2008-2011). IBD networks were constructed at the 47.5% and 95% thresholds (2008–2011 samples), with nodes coloured by *pfkelch13* mutation status (A, B) and country of origin (C, D). Source data are provided as a Source Data file (source_data_2).

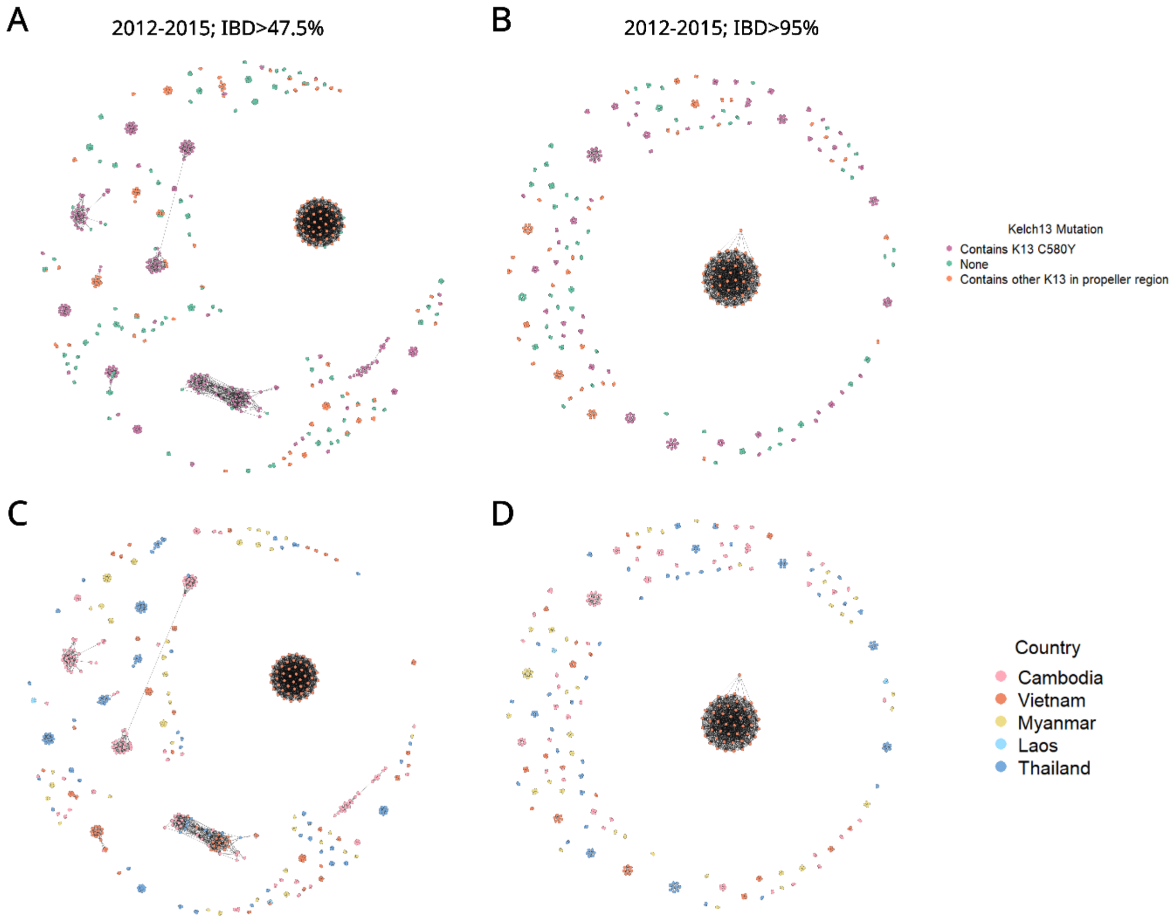

Supplementary Figure 21. Identity-by-descent (IBD) networks in Southeast Asia showing clusters at the 47.5% and 95% pairwise IBD thresholds (2012-2015). IBD networks were constructed at the 47.5% and 95% thresholds (2012–2015 samples), with nodes coloured by *pfkelch13* mutation status (A, B) and country of origin (C, D). Source data are provided as a Source Data file (source_data_2).

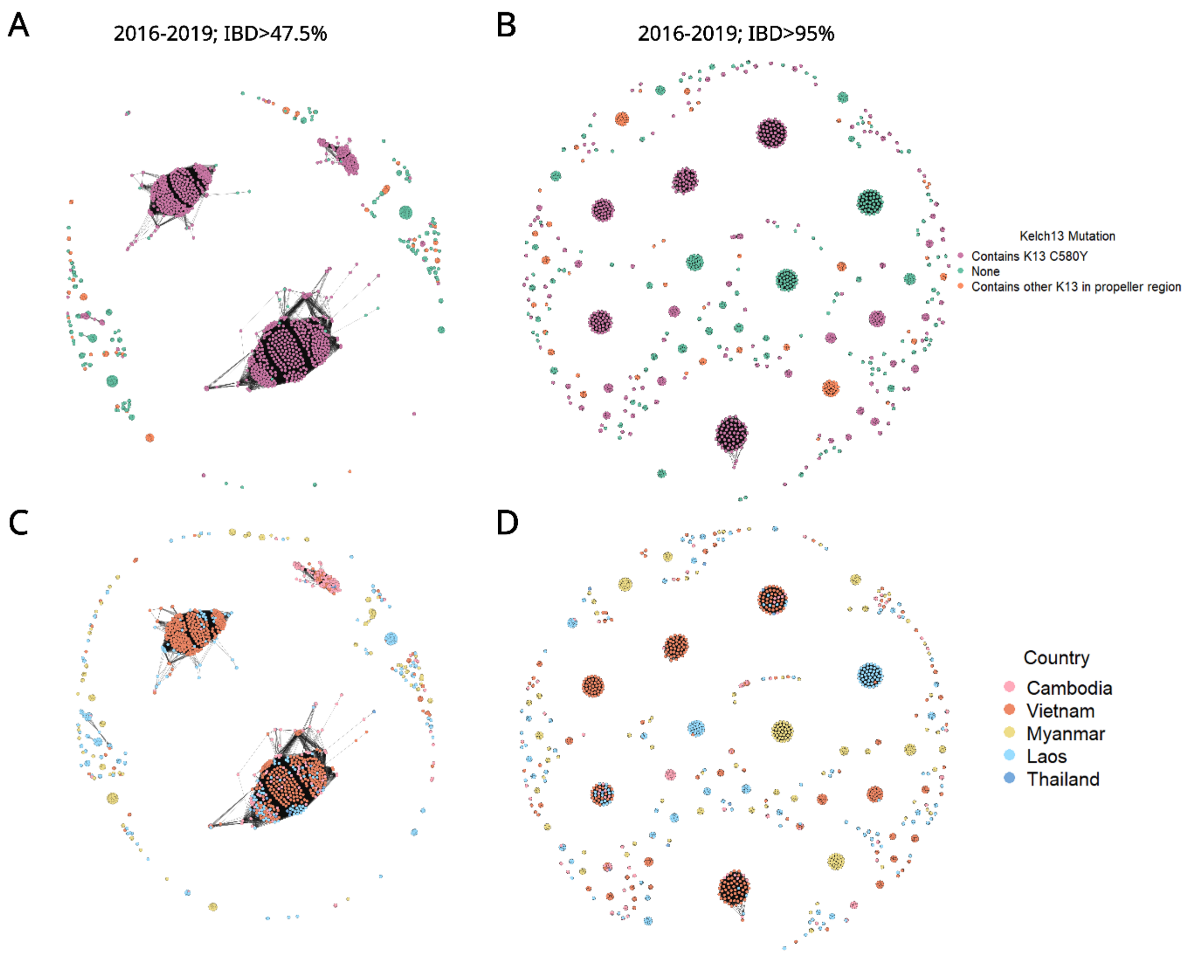

Supplementary Figure 22. Identity-by-descent (IBD) networks in Southeast Asia showing clusters at the 47.5% and 95% pairwise IBD thresholds (2016-2019). IBD networks were constructed at the 47.5% and 95% thresholds (2016–2019 samples), with nodes coloured by *pfkelch13* mutation status (A, B) and country of origin (C, D). Source data are provided as a Source Data file (source_data_2).

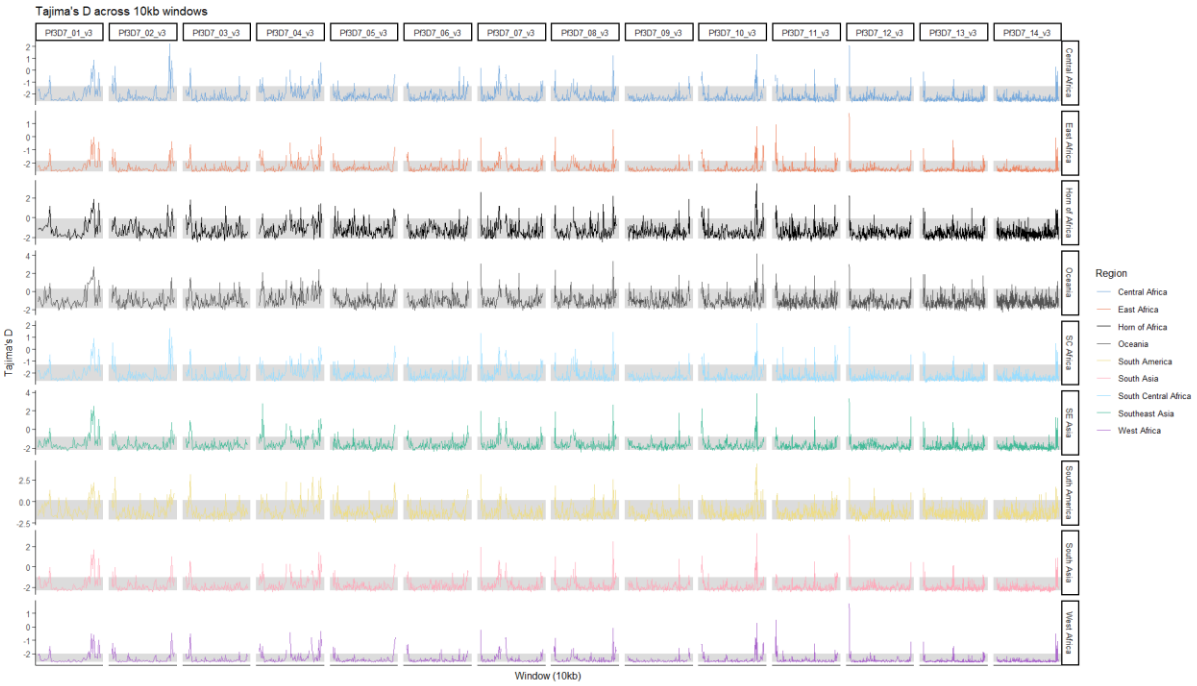

Supplementary Figure 23. Tajima's D calculations for 14 chromosomes and 9 geographic regions. Tajima's D calculations estimated using 10kb windows are shown for each chromosome and region. SC= South Central; SE= Southeast. Areas above and below shading indicate 5% and 95% Tajima D cutoffs, respectively, calculated individually for each geographic region. Source data are provided as a Source Data file.

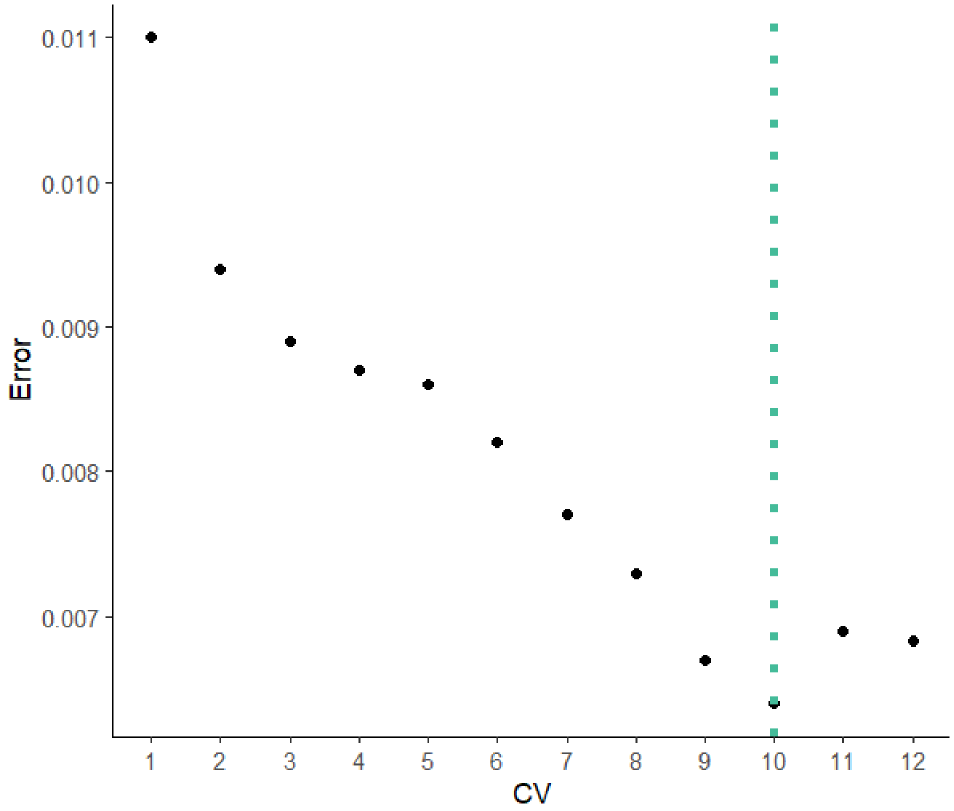

Supplementary Figure 24. Cross-validation results for optimal K selection for ADMIXTURE analysis. Cross-validation (CV) Errors are shown for K=1:12 and optimal K=10 is highlighted at the inflection point. Source data are provided as a Source Data file.
