# Supplementary material for: Global-scale population genetic analysis of Plasmodium falciparum identifies region-specific patterns of malaria parasite adaptation
Source: Nat Commun. 2026 May 11;17:6318. doi: 10.1038/s41467-026-73006-2 (PMC13377190; doi:10.1038/s41467-026-73006-2)
Supplement: Supplementary file 2 — Description of Additional Supplementary Files [file 41467_2026_73006_MOESM2_ESM.pdf]

Global-scale population genetic analysis of *Plasmodium falciparum* identifies country- and region-specific patterns of malaria parasite adaptation

Title: Supplementary Data 1
Description: Summary of previous global-scale and regional level population genetics analyses of *P. falciparum* whole genome sequences and the unique contributions of this study.

Title: Supplementary Data 2
Description: Number of samples (N = 17,565) by geographic location.

Title: Supplementary Data 3
Description: Fws scores per country and year group (category).

Title: Supplementary Data 4
Description: Single nucleotide polymorphisms with high FST (>0.75) for pairwise regional comparisons (MAF > 0.1).

Title: Supplementary Data 5
Description: Gene Ontology analysis for biological processes, cellular components and molecular functions for genes with FST scores (pairwise comparisons).

Title: Supplementary Data 6
Description: Single nucleotide polymorphisms with high FST (>0.75) for pairwise country-level comparisons.

Title: Supplementary Data 7
Description: Single nucleotide polymorphisms with high FST (>0.75) for each region ("one-against-all").

Title: Supplementary Data 8
Description: Single nucleotide polymorphisms with high FST (>0.75) for each country ("one-against-all").

Title: Supplementary Data 9
Description: Chromosomal regions with high IBD fractions (95% quartile) and their corresponding genes and gene products.

Title: Supplementary Data 10
Description: Gene Ontology enrichment for biological processes, cellular components and molecular functions for genes with high IBD fractions.

Title: Supplementary Data 11
Description: Genes with high iR (-log10 P-value > 5) across countries and time.

Title: Supplementary Data 12
Description: pfkelch13 C580Y interaction candidates (co-occurrence and association).

Title: Supplementary Data 13
Description: Composite selection results by geographic region.

Title: Supplementary Data 14
Description: Genomic windows showing evidence of positive selection across regions.

Title: Supplementary Data 15
Description: Moderate FST signals (FST > 0.2) in selection candidate genes.

Title: Supplementary Data 16
Description: Chromosomal regions with high iHS scores.

Title: Supplementary Data 17
Description: Chromosomal regions with high XP-EHH scores and their corresponding genes and gene products.

Title: Supplementary Data 18
Description: Regions of the genome in the top 95% percentile of IBD fractions for samples from Brazil.

Title: Supplementary Data 19
Description: Regions of the genome in the top 95% percentile of IBD fractions for samples from Vietnam (newly sequenced).

Title: Supplementary Data 20
Description: Metadata for global *P. falciparum* dataset.

Title: Supplementary Data 21
Description: Highly variable regions excluded in selection analysis.

Title: Supplementary Data 22
Description: Prioritised Kelch13 interaction candidate gene IDs.
