# Supplementary material for: Global-scale population genetic analysis of Plasmodium falciparum identifies region-specific patterns of malaria parasite adaptation
Source: Nat Commun. 2026 May 11;17:6318. doi: 10.1038/s41467-026-73006-2 (PMC13377190; doi:10.1038/s41467-026-73006-2)
Supplement: Supplementary file 4 — Reporting Summary [file 41467_2026_73006_MOESM4_ESM.pdf]

nature portfolio

Corresponding author(s): Taane G. Clark (Taane.Clark@LSHTM.ac.uk)

Last updated by author(s): Mar 12, 2026

Reporting Summary

Nature Portfolio wishes to improve the reproducibility of the work that we publish. This form provides structure for consistency and transparency in reporting. For further information on Nature Portfolio policies, see our Editorial Policies and the Editorial Policy Checklist.

Statistics

For all statistical analyses, confirm that the following items are present in the figure legend, table legend, main text, or Methods section.

n/a	Confirmed	
☐	☒	The exact sample size (n) for each experimental group/condition, given as a discrete number and unit of measurement
☐	☒	A statement on whether measurements were taken from distinct samples or whether the same sample was measured repeatedly
☐	☒	The statistical test(s) used AND whether they are one- or two-sided<br>*Only common tests should be described solely by name; describe more complex techniques in the Methods section.*
☐	☒	A description of all covariates tested
☐	☒	A description of any assumptions or corrections, such as tests of normality and adjustment for multiple comparisons
☐	☒	A full description of the statistical parameters including central tendency (e.g. means) or other basic estimates (e.g. regression coefficient) AND variation (e.g. standard deviation) or associated estimates of uncertainty (e.g. confidence intervals)
☐	☒	For null hypothesis testing, the test statistic (e.g. F, t, r) with confidence intervals, effect sizes, degrees of freedom and P value noted<br>*Give P values as exact values whenever suitable.*
☒	☐	For Bayesian analysis, information on the choice of priors and Markov chain Monte Carlo settings
☒	☐	For hierarchical and complex designs, identification of the appropriate level for tests and full reporting of outcomes
☐	☒	Estimates of effect sizes (e.g. Cohen's d, Pearson's r), indicating how they were calculated

Our web collection on statistics for biologists contains articles on many of the points above.

Software and code

Policy information about availability of computer code

Data collection	No software was used for data collection.
Data analysis	All raw sequencing reads were processed using the fastq2matrix pipeline (https://github.com/pathogenseq/fastq2matrix/), which is composed of existing software tools. Data analysis was performed using custom in-house scripts available at , which make use of standard population genetics analysis tools https://github.com/LSHTMPathogenSeqLab/Pop_Gen. No novel software was developed for this study; only analysis scripts were created. Software include: Trimmomatic v0.39, bwa-mem v0.7.17-r1188, samtools v1.18, GATK v4.1.4.1, snpEff v5.1, PLINK v1.90, R v4.2.2, scikit-allel v1.3.7, vegan – R, bcftools v1.20, moimix v0.0.2.9001 – R, ADMIXTURE v1.3, hmmIBD – R, isoRelate – R, rehh – R, VCFtools v0.1.16, PANTHER – R, ipysigma – R, LEA – R and STRING.

For manuscripts utilizing custom algorithms or software that are central to the research but not yet described in published literature, software must be made available to editors and reviewers. We strongly encourage code deposition in a community repository (e.g. GitHub). See the Nature Portfolio guidelines for submitting code & software for further information.

Data

Policy information about availability of data

All manuscripts must include a data availability statement. This statement should provide the following information, where applicable:

- Accession codes, unique identifiers, or web links for publicly available datasets
- A description of any restrictions on data availability
- For clinical datasets or third party data, please ensure that the statement adheres to our policy

Raw sequencing data are available in the European Nucleotide Archive (ENA). A complete list of accession numbers is provided in PRJEB94034. This publication also uses data from the MalariaGEN Plasmodium falciparum Community Project as described in ‘An open dataset of Plasmodium falciparum genome variation in 7,000 worldwide samples. MalariaGEN et al, Wellcome Open Research 2021642 DOI: 10.12688/wellcomeopenres.16168.1. Source data are provided with this paper. Accessions for all samples used in this study can be found in Supplementary Table 20.

Research involving human participants, their data, or biological material

Policy information about studies with human participants or human data. See also policy information about sex, gender (identity/presentation), and sexual orientation and race, ethnicity and racism.

Reporting on sex and gender	No human participants or human data were used or involved in this study.
Reporting on race, ethnicity, or other socially relevant groupings	No human participants or human data were used or involved in this study.
Population characteristics	No human participants or human data were used or involved in this study.
Recruitment	No human participants or human data were used or involved in this study.
Ethics oversight	All parasite DNA was extracted from human blood samples obtained from malaria patients who provided informed consent. The newly sequenced collections were approved by the research ethics committees of the National Institute of Malariology, Parasitology, and Entomology, Ministry of Health, Vietnam (1096/QĐ-VSR) and the University of São Paulo, Brazil and Plataforma Brasil under two protocols: CAAE 32707720.0.0000.5467 (5.855.979; 2013–2014) and CAAE 32947520.4.0000.5467 (6.161.402; 2020–2021)

Note that full information on the approval of the study protocol must also be provided in the manuscript.

Field-specific reporting

Please select the one below that is the best fit for your research. If you are not sure, read the appropriate sections before making your selection.

☒ Life sciences ☐ Behavioural & social sciences ☐ Ecological, evolutionary & environmental sciences

For a reference copy of the document with all sections, see nature.com/documents/nr-reporting-summary-flat.pdf

Life sciences study design

All studies must disclose on these points even when the disclosure is negative.

Sample size	No formal sample size calculation was performed. Sample sizes were determined by the availability of high-quality P. falciparum whole-genome sequence data. We maximised sample numbers by adopting a convenience sampling framework, considering all publicly available sequences from the MalariaGEN Pf7 dataset and previously published studies, supplemented by 76 newly sequenced isolates from Brazil and Vietnam. After quality filtering (removal of duplicates, mixed-species infections, and samples with ≥60% of the genome at coverage <5×), 17,565 high-quality isolates from 39 countries were retained for analysis.This sample size is sufficient for the analyses performed. Population structure inference, identity-by-descent analyses, and genome-wide selection scans are not hypothesis-driven experiments with a pre-specified effect size; rather, their power scales with the number and geographic breadth of samples available. A dataset of 17,565 isolates spanning 39 countries represents one of the largest P. falciparum genomic datasets analysed to date, providing substantial power to detect population structure, recent haplotype sharing, and signatures of positive selection at both global and regional scales. The geographic diversity of the dataset further ensures that region-specific patterns of adaptation and drug-resistance spread can be resolved with confidence.
Data exclusions	Samples were excluded if they were duplicates, mixed-species infections, or had low coverage (≥60% of the genome with coverage <5×).
Replication	Samples were used for primary analysis. To ensure reproducibility, all analyses were performed using documented pipelines and custom scripts that are publicly available. Standard population genetics tools were used with fixed parameters, and key analyses (e.g., PCA, FST, allele frequency calculations) were repeated on independent subsets to confirm robustness.
Randomization	Not applicable as this is a cohort analysis. Samples were allocated into groups based on geographic origin, covering 39 countries, and for temporal analyses, into three-year intervals. No randomization was applied, as group assignments were determined by sample collection location and date rather than experimental manipulation.
Blinding	Not applicable as this is a cohort analysis.

Reporting for specific materials, systems and methods

We require information from authors about some types of materials, experimental systems and methods used in many studies. Here, indicate whether each material, system or method listed is relevant to your study. If you are not sure if a list item applies to your research, read the appropriate section before selecting a response.

Materials & experimental systems

n/a	Involved in the study
☒	☐ Antibodies
☒	☐ Eukaryotic cell lines
☒	☐ Palaeontology and archaeology
☒	☐ Animals and other organisms
☒	☐ Clinical data
☒	☐ Dual use research of concern
☒	☐ Plants

Methods

n/a	Involved in the study
☒	☐ ChIP-seq
☒	☐ Flow cytometry
☒	☐ MRI-based neuroimaging

Plants

Seed stocks	*Report on the source of all seed stocks or other plant material used. If applicable, state the seed stock centre and catalogue number. If plant specimens were collected from the field, describe the collection location, date and sampling procedures.*
Novel plant genotypes	*Describe the methods by which all novel plant genotypes were produced. This includes those generated by transgenic approaches, gene editing, chemical/radiation-based mutagenesis and hybridization. For transgenic lines, describe the transformation method, the number of independent lines analyzed and the generation upon which experiments were performed. For gene-edited lines, describe the editor used, the endogenous sequence targeted for editing, the targeting guide RNA sequence (if applicable) and how the editor was applied.*
Authentication	*Describe any authentication procedures for each seed stock used or novel genotype generated. Describe any experiments used to assess the effect of a mutation and, where applicable, how potential secondary effects (e.g. second site T-DNA insertions, mosiacism, off-target gene editing) were examined.*
