# Supplementary material for: Global-scale population genetic analysis of Plasmodium falciparum identifies region-specific patterns of malaria parasite adaptation
Source: Nat Commun. 2026 May 11;17:6318. doi: 10.1038/s41467-026-73006-2 (PMC13377190; doi:10.1038/s41467-026-73006-2)
Supplement: Supplementary file 5 — Transparent Peer Review file [file 41467_2026_73006_MOESM5_ESM.pdf]

nature portfolio

Peer Review File

Global-scale population genetic analysis of Plasmodium falciparum identifies region-specific patterns of malaria parasite adaptation

Corresponding Author: Dr Nina Billows

This file contains all reviewer reports in order by version, followed by all author rebuttals in order by version.

Version 0:

Reviewer comments:

Reviewer #1

(Remarks to the Author)
Billows et al. describe an analysis of previously-release whole genome sequence data representing a global collection of 17,565 Plasmodium falciparum malaria parasites, supplemented with 76 new sequenced genomes from Brazil and Vietnam. The authors reinvestigate the global diversity and divergence of parasite populations, and re-examine selection signals across the genome using conventional approaches.

Many of the conclusions presented in this manuscript have been published in previous analyses of the data re-analyzed here. Other analysis outcomes, for example changes in Fws over time potentially signifying changes in transmission level within countries/regions, are very difficult to support without much more detailed information about the exact methodology by which samples were collected. For example, Fws could look very different for samples collected passively at a clinic in a large city vs. actively within a few households of a small village. This challenge keeps most of the reported conclusions at a superficial level.

The 76 newly added genome sequences do not appear to contribute meaningfully to the conclusions presented. The analyses recapitulate many previous analyses on the pre-existing genome sequences, and do not contribute meaningful new insights. Though this manuscript represents a lot of work, and most of the analyses appear to have been performed competently, it is therefore difficult to see the rationale for publishing this manuscript.

Minor comments

Line 197: It is necessary to define the term ‘Multiplicity of Infection’, which has a different meaning in the malaria literature than the literature for any other infectious disease (where it signifies the numerical ratio of infectious agents to susceptible cells). MOI, or complexity of infection, are typically represented by integer estimates rather than continuous Fws values, so this section would be more appropriately labeled ‘polyclonality’ or similar, unless the authors include estimates of MOI/COI.

Figure 1: It is difficult to distinguish some of the colors used in the MDS analysis, for example South American vs. SouthEast Asia. Font sizes on the x axis labels of panel C are too small and illegible.

Figure 2: It is difficult to take valuable information from this figure. If the point is that inter vs. intra-continental population comparisons show a difference in Fst, those divergence estimates could be represented by box and whisker plots, for example, with statistically significant differences noted. It is not clear why some regions (South America, South Asia, SE Asia, etc.) with multiple sampling localities are collapsed to single regions rather than used for intra-region comparisons. However, the overall motivation for this analysis is not clear; are there one or more population comparisons that yielded unexpected results in accord with a hypothesis about population connectivity or shared ancestry, for example?

Figure 3: Font is too small. Pie charts are too small. This map is unhelpful. For an academic audience, it would be much more useful to summarize the regional prevalence of resistance mutations/haplotypes directly, rather than reporting the coarser inferred phenotypes based on an unclear mapping algorithm from the genotypes.

Figure 4: font is too small everywhere. The legend is missing a callout to one of the panels and is confusing to read. The protein-protein interaction network in panel F is difficult to interpret; what should readers take from this?

(Remarks on code availability)

Reviewer #2

(Remarks to the Author)
Review of Global-scale population genetic analyses of Plasmodium falciparum identifies country- and region-specific patterns of malaria parasite adaptation

In this manuscript, P. falciparum whole genome sequence data are analysed generating a comprehensive profile of P. falciparum population genetics on a global scale. These profiles are important given that P. falciparum's ever-evolving nature impacts the design of effective disease control measures.

The profile is based on a series of genetic analyses including:

• Computation of diversity and Fws statistics (correlates of transmission)
• Multidimensional scaling and population assignment to compare how genetic clustering compares with geography
• Intra-region genome-averaged pairwise IBD as a measure of diversity and to interrogate soft selective sweeps around pfkelch13
• Population-averaged genome-wide IBD scans to identify genomic regions with signals of selection
• FST as a measure of divergence
• GWAS to identify associations with genetic markers in pfkelch13 in SE Asia

The data include data on samples in MalariaGen's Pf7 data release, data on ~7000 previously published samples, and data on 76 newly sequenced samples from Brazil and Vietnam. The results are consistent with our current understanding of transmission. Markers of artemisinin resistance are not among the many SNPs / regions with population-averaged signals of selection. But localised networks of pairwise IBD are consistent with selection. In addition, genome-wide associations support a polygenic model of artemisinin resistance.

The manuscript is well written. In particular, the authors take care to interpret their results speculatively. This is important given these types of studies (studies that feature so many inter-related analyses) are likely to generate many false positives.

Major suggestions

1. This study would be more impactful if it were more explicitly compared with existing global-scale P. falciparum population-genetic profiles and other C580Y genome wide association studies. The authors could tabulate the overlap and differences, both in terms of data and results, across studies over time. For example, have the ~7000 previously published samples (line 724) been analysed already alongside Pf7 in a global-scale study? On a different but related note, in one of the supplementary tables there appear to be only 46 "not Pf7" samples in the analysis-ready data set. How can this be given the inclusion of ~7000 previously published samples? How many of these 46 "not Pf7" samples were newly sequenced?

2. The emergence of partial resistance to artemisinin in Africa is a major current concern. To line 471, I would add a sentence referring to how and why the results in the current study don't likely capture the current situation in Africa, referring to sample dates. And then again refer to sample dates in the sentence spanning lines 479 to 483. On a different but related note, the authors refer to full artemisinin resistance (line 479). What is meant by full resistance? I was not aware of reports of full artemisinin resistance.

Minor suggestions

4. Figure 1: I don't understand Figure 1B or the reference to it within the caption. What insight is Fig1B adding above and beyond Fig1A and Fig1C? Where is Fig1B referred to in the main text? Why are the axes in Fig1B PC1 and PC2 and not D1 and D2 as in Fig1A? What is the maximum k value of an individual isolate? Why does the caption mention Admixture in between (A) and (B)?
5. Figure 3: the number of categories is not amenable to visualisation. I would reduce the number of categories to those with visible proportions plus "other" which can be defined in the caption.
6. Figure 4: IBD networks suggest intra-mutation associations progressively replace intra-country associations. However, these associations are likely confounded by imbalanced sample counts in different time points. Sample counts should be added to the per-mutation and per-country legends. In addition, reference to Sup Fig 15 in the caption of Fig 4 would aid interpretation of Fig 4B (it is not obvious from Fig 4 alone that the strong association between other K13 mutations in 2012-2015 is possibly explained by strong intra-Vietnam relatedness).
7. Sup Fig1: label-to-cluster links are unclear, e.g., which cluster does 'Venezuela' refer to? An opaque version of the cluster colour could be used to outline the cluster and print the label.
8. Supplementary Figure 8: I like this figure. Does it depict the top 5% (line 250) of all high-IBD regions or the top 5% per

region? It would be nice to annotate the positions of the named loci discussed in the paragraph beginning line 256.
9. The addition of a very brief intuitive description of each analysis each time a new one is introduced (already done for Tajima's D) would help readers interpret results; e.g., adding a few words to the parenthesis on line 154 "(MDS — a clustering analysis…)", adding a short parenthesis after "Gene Ontology (GO) term overrepresentation analysis" on line 256; coping the phrase "whereby … in the analysis" on lines 769-770 to line 32. Likewise, reference to H12 (line 452) is meaningless without extra info: at the very least, add a reference.
10. I don't understand the threshold on line 345 (e.g., why write 1 – 2 and not -1? What does the conditioning bar "|" represent?). Without additional information its meaningless. I suggest the reviewers either unpack it or move it to the methods.
11. I really appreciate the cautious language. Nevertheless, there are still some places that could be tempered: line 240 ("likely reflect"), line 346 ("were considered to be under recent positive selection"); line 462 ("prevalence of genetic markers of resistance"); line 610 ("likely shaped"); line 612 ("likely reflecting").
12. Line 391, 395: does sites refer to SNPs? If so, I would stick with SNPs.
13. I was surprised by the results on lines 444 and 445: I expect evidence of balancing to be greatest in high-transmission. Can the authors expand on this?
14. Supplementary Fig 11: it might be nice to delimit -2 and 2 with e.g., a central shaded band. West Africa is almost entirely below the -2 limit. Do these limits make sense for genome-wide scans? It looks like there might be some statistical artefact that requires a per-region adjustment.
15. Lines 637 to 641: Do these statements agree with incidence data where available?
16. I don't understand lines 753-754: are heteroallelic calls with MAF < 20% reduced to homoallelic calls? Please re-phrase.
17. Were multiclonal isolates removed from all but Fws analyses? Might this bias any of the conclusions?
18. Line 796: "Hidden Markov model" (delete chain)

(Remarks on code availability)
I have only glanced at the github repository. I have not tried to run the code. The documentation is excellent.

Reviewer #3

(Remarks to the Author)
The authors have presented data on 17,565 Plasmodium falciparum isolates collected from 39 different malaria endemic countries of the globe and performed several population genetic analyses under different models to infer P. falciparum's evolution, patterns of gene flow and genetic patterns of drug resistance in these populations. While they have reconstructed history of P. falciparum populations, they also have unravel signatures of natural selection in drug-resistant loci. With these findings, they interpreted that the knowledge will impact malaria control efforts and also guide malaria control strategies. These analyses are extensive and the dataset is substantial. However, several concerns remain to be addressed, especially regarding novelty and completeness of this study.

Across the P. falciparum populations, based on published literate at local level, many of the factual history of population structure, gene flow, isolation-by-distance, etc. that the authors have presented are already known. These are principally driven consequences of P. falciparum's transmission dynamics, which is fundamentally different from those of, Plasmodium vivax. For example, the finding on the location of the genetic loci/ nucleotide sites under selection are located in highly variable sites are hallmark of the evolutionary principles. Furthermore, it is well known that the surface antigenic genes in malaria parasites in general, and P. falciparum in particular are known to be highly variable and under strong diversifying selection. Similarly, loci involved in host-parasite interactions have been repeatedly shown to exhibit strong selective pressure in both host and parasitic genomes (e.g. the DARC in humans, and PvDBP P. vivax). Therefore, the observation on selection hot-spots at the chromosomal level does not represent a novel contribution in itself, while accurately described.

One of the most critical gaps in the manuscript is the absence of genomic data from P. falciparum isolates originating from India. India remains one of the most malaria-endemic countries globally, and prior studies have highlighted the unique genetic and evolutionary characteristics of Indian P. falciparum populations. For example; i) Indian P. falciparum show several ancestral properties, such as highest genetic diversity than the African P. falciparum, suggesting an ancestral origin (Tyagi and Das, 2025, Mitochondrion). Furthermore, (ii) a distinct ancestral genetic lineage, found in India, has been proposed as the missing-link in host switch of P. falciparum from non-human primates (rhesus macaque) to humans (Tyagi et al., 2014, Molecular Ecology, reviewed in Das, 2015; Trends in Parasitology). Moreover, (iii) zoonotic P. falciparum infections have been detected in Indian rhesus and bonnet macaque for the first time (Dixit et al. PLOS Neglected Tropical Diseases, 2018), pointing to a possible primate reservoir. These define unique features of P. falciparum in India, and to my opinion leaving behind this important corridor of human (and P. falciparum) ancestral migration will not justify reconstruction of evolutionary history of P. falciparum at the global level.

(Remarks on code availability)
Not applicable

Version 1:

Reviewer comments:

Reviewer #1

(Remarks to the Author)
The authors have responded thoroughly to my original comments and have clarified many aspects of the manuscript. However, the novel aspects of this manuscript remain a minor component of the overall document, and their value is unclear because they are under-explored. For example, the abstract now references the detection of previously unreported selection signals at DHODH, Eps150-like protein, and MCA2, loci previously associated with drug resistance in various contexts. However, these selection analyses are described in supplementary figures rather than main figures, while the main figures retain their focus on findings that are not original or surprising, such as the observation of greater Fst between continents than within continents (Figure 2), or the association of clonality with the emergence of K13 mutations in SE Asia (Figure 4).

The novel results are only explored superficially, and therefore it remains unclear if readers should ascribe value to them. For example, the selection signal at DHODH appears to take the form of a significant iHS p value for Central Africa. Does the iHS selection at DHODH signal associate with a concordant enrichment in IBD in that population, or a particularly low Tajima's D value? Are there nonsynonymous polymorphisms with unusually high minor allele frequency in DHODH in Central Africa, or what could be driving the iHS result? Why do the authors speculate there would be selection on DHODH, when the small molecules that have been observed to drive resistance mutations at DHODH have not been approved for clinical use?

The focus of the paper should be changed to more deeply exploring novel observations to warrant publication.

(Remarks on code availability)

Reviewer #2

(Remarks to the Author)
The authors have addressed my points, but they should make the relative contribution of this study relative to other global analyses clearer upfront:

1. Add citations for the major “earlier global analyses” in the last paragraph of the Background and refer the reader to Supplementary Table 19.
2. In the Background state something along the lines of “Although this study goes into more analytic detail compared with other global analyses, the global analysis of MalariaGEN 2025 represents a larger and more up-to-date global data set”
3. In the first section of the results: in addition to showing how the 17,565 samples break down geographically, describe how they break down into previously analysed datasets (XX in Pf7, XX in Pf8 but not Pf7, plus 76 analysed here first).

In addition, some minor suggestions:

4. Abstract “Polygenic backgrounds associated with artemisinin resistance” on line 46 implies the manuscript has in vitro / clinical data on resistance. Do you mean “Polygenic backgrounds associated with genetic markers of artemisinin resistance” ?

5. Line 95: add artemisinin: “signs of reduced artemisinin combination therapy (ACT)”

6. Fig 1: Why do some points coloured with the grey of Oceania in panel A disappear in panel B?

7. Fig 1 caption: Points are coloured, not “Dimensions”

8. Line 155 and Supp. Fig 1: I don’t see any samples with a colour assigned to Ecuador, only Colombia.

9. Supp. Fig 1: caption mentions non-existent panel B

10. Line 172: I don’t see any SE Asian isolates that exhibit Wast African Ancestry in Fig 1.

11. Section entitled “Integration of New Regional Datasets into Global Population Genomics”: please site the studies detailing sample collection for the 76 new sequences.

(Remarks on code availability)
I scanned it last review and it looked solid.

Reviewer #3

(Remarks to the Author)
The concerns raised are appropriately and adequately addressed in the revised manuscript.

(Remarks on code availability)

Version 2:

Reviewer comments:

Reviewer #3

(Remarks to the Author)
I find the revised manuscript substantially improved in comparison to the previous version.

(Remarks on code availability)
Not applicable

Open Access This Peer Review File is licensed under a Creative Commons Attribution 4.0 International License, which permits use, sharing, adaptation, distribution and reproduction in any medium or format, as long as you give appropriate credit to the original author(s) and the source, provide a link to the Creative Commons license, and indicate if changes were made.
In cases where reviewers are anonymous, credit should be given to 'Anonymous Referee' and the source.
The images or other third party material in this Peer Review File are included in the article's Creative Commons license, unless indicated otherwise in a credit line to the material. If material is not included in the article's Creative Commons license and your intended use is not permitted by statutory regulation or exceeds the permitted use, you will need to obtain permission directly from the copyright holder.
To view a copy of this license, visit https://creativecommons.org/licenses/by/4.0/

Reviewers' comments:

Reviewer 1

Major comments:

1. Billows et al. describe an analysis of previously-release whole genome sequence data representing a global collection of 17,565 *Plasmodium* falciparum malaria parasites, supplemented with 76 new sequenced genomes from Brazil and Vietnam. The authors reinvestigate the global diversity and divergence of parasite populations, and re-examine selection signals across the genome using conventional approaches. Many of the conclusions presented in this manuscript have been published in previous analyses of the data re-analyzed here. Other analysis outcomes, for example changes in Fws over time potentially signifying changes in transmission level within countries/regions, are very difficult to support without much more detailed information about the exact methodology by which samples were collected. For example, Fws could look very different for samples collected passively at a clinic in a large city vs. actively within a few households of a small village. This challenge keeps most of the reported conclusions at a superficial level.

We thank the Reviewer for the time they have taken to carefully review our manuscript. We have taken these comments on board and re-ran the selection analyses and rewritten parts of the manuscript, including the Abstract, to bring the novel outcomes of our analyses to light. This included removing highly variable genes from the selection analyses to identify other, novel, genomic loci under selective pressure. This led to several interesting findings, including in Central Africa, where we uncovered selection at *DHODH (PF3D7_0603300), PF3D7_1025000* (Eps15-like protein) and *MCA2*, highlighting (recent) selective pressure in drug-resistance–associated loci distinct from those observed in Southeast Asia (line 602-717). This is an important finding as countries such as the Democratic Republic of the Congo have reported widespread ACT failure and a lack of association with K13 mutations (Yobi *et al.,* 2020). Across African regions, we observe coordinated selection in *UBP1*, a gene previously suggested to be involved with artemisinin resistance. This is another important finding, as even if there is a low frequency of ACT resistance in these regions, it is important to understand the genomic context where resistance may arise. As per the suggestion from Reviewer 2, we have included a new table (Table S19) that showcases the contribution of our global analysis compared to previously published and recent global analysis papers. Most global studies have not carried out detailed admixture, identity by descent and selection analysis. Therefore, we believe we do make a unique contribution through our in-depth analyses. Furthermore, we have taken the feedback regarding F_{WS} on board. We have included additional discussion on this analysis in the Results and Discussion sections to highlight the limitations and the importance of sampling approaches when estimating the F_{WS} statistic. We note that similar statistics are reported in published Pf3, Pf7 and Pf8 manuscripts, and can provide valuable insights into the heterozygosity present within parasite populations, but we agree they should be carefully interpreted.

2. The 76 newly added genome sequences do not appear to contribute meaningfully to the conclusions presented. The analyses recapitulate many previous analyses on the pre-existing genome sequences, and do not contribute meaningful new insights. Though this manuscript represents a lot of work, and most of the analyses appear to have been performed competently, it is therefore difficult to see the rationale for publishing this manuscript.

We thank you for this comment. We have now included a section that dives more deeply into the 76 newly sequenced samples from Brazil and Vietnam, and believe these samples contribute significantly to the novelty of our study (lines 1038-1066). Throughout the manuscript, we demonstrated that the inclusion of the samples from Brazil improves the overall global picture of *P. falciparum* populations, especially considering population structure, admixture and genetic differentiation (F_{ST}) analyses. Existing global datasets (Pf7, Pf8) do not have any samples from Brazil or other South American countries, including Guyana and French Guiana, which have shown to have distinct population structure and drug-resistance profiles (e.g., *pfcrt* C350R and *pfkelch13* R561H in Guyana). Therefore, the inclusion of new samples from Brazil is of significant interest. Additionally, analysis of new samples from Vietnam (N=18) revealed all samples had the *pfkelch13* C580Y with high IBD fractions in the region of the genome containing *KIC13,* which is found within the same cellular compartment as KELCH13. This suggests an additional mechanism which could compensate for fitness loss associated with or exacerbate artemisinin resistance, meriting further investigation.

Minor comments

1. Line 197: It is necessary to define the term ‘Multiplicity of Infection’, which has a different meaning in the malaria literature than the literature for any other infectious disease (where it signifies the numerical ratio of infectious agents to susceptible cells). MOI, or complexity of infection, are typically represented by integer estimates rather than continuous Fws values, so this section would be more appropriately labeled ‘polyclonality’ or similar, unless the authors include estimates of MOI/COI.

Thank you for this suggestion. We have renamed this part of the manuscript.

2. Figure 1: It is difficult to distinguish some of the colors used in the MDS analysis, for example South American vs. SouthEast Asia. Font sizes on the x axis labels of panel C are too small and illegible.

Thanks. We have recoloured all plots and changed the size of the x-axis for panel C. We have also made changes to Figure 1, as per comments from Reviewers 2 and 3.

3. Figure 2: It is difficult to take valuable information from this figure. If the point is that inter vs. intra-continental population comparisons show a difference in Fst, those divergence estimates could be represented by box and whisker plots, for example, with statistically significant differences noted. It is not clear why some regions (South America, South Asia, SE Asia, etc.) with multiple sampling localities are collapsed to single regions rather than used for intra-region comparisons. However, the overall

motivation for this analysis is not clear; are there one or more population comparisons that yielded unexpected results in accord with a hypothesis about population connectivity or shared ancestry, for example?

Thank you. The purpose of this figure was to highlight inter- and intra-continental F_{ST} comparisons. We performed these analyses both at 'regional' and 'country' levels. We have produced the boxplot suggested and highlighted the unexpected results within South America.

4. Figure 3: Font is too small. Pie charts are too small. This map is unhelpful. For an academic audience, it would be much more useful to summarize the regional prevalence of resistance mutations/haplotypes directly, rather than reporting the coarser inferred phenotypes based on an unclear mapping algorithm from the genotypes.

We thank the Reviewer for the suggestion. We have now integrated drug-resistant haplotypes into our analysis and produced heatmaps and associated bar charts to indicate regional differences in drug-resistant haplotypes (Figure 3). We have removed the map and coarser phenotypes entirely.

5. Figure 4: font is too small everywhere. The legend is missing a callout to one of the panels and is confusing to read. The protein-protein interaction network in panel F is difficult to interpret; what should readers take from this?

Thank you. We have increased the font size of the plots and refined the figure legends. We have removed the PPI network as per the Reviewer's suggestion, as it is confusing to interpret. The overall plot size has also been increased.

<u>Reviewer 2</u>

The manuscript is well written. In particular, the authors take care to interpret their results speculatively. This is important given these types of studies (studies that feature so many inter-related analyses) are likely to generate many false positives.

Major suggestions

1. This study would be more impactful if it were more explicitly compared with existing global-scale P. falciparum population-genetic profiles and other C580Y genome wide association studies. The authors could tabulate the overlap and differences, both in terms of data and results, across studies over time. For example, have the ~7000 previously published samples (line 724) been analysed already alongside Pf7 in a global-scale study? On a different but related note, in one of the supplementary tables there appear to be only 46 "not Pf7" samples in the analysis-ready data set. How can this be given the inclusion

of ~7000 previously published samples? How many of these 46 "not Pf7" samples were newly sequenced?

We thank the Reviewer for the constructive feedback and for taking the time to review our manuscript and make helpful suggestions. We have considered that the study would be more impactful if directly compared with previous studies. Therefore, we include a table of comparison in Table S19. This table shows findings across regional and global-level studies, including those that have been curated alongside the Pf7 dataset in our study. Notably, past global-level studies of a similar size do not perform selection, identity by descent (IBD) or admixture analysis, highlighting the value of our study. We also apologise if the number of sequences used is unclear in the supplementary table. Samples in Pf7 are indicated by the 'Pf7' column, if filtered for 'FALSE', 2,717 samples, including 76 samples, were not in Pf7. We have clarified this on line 1373.

2. The emergence of partial resistance to artemisinin in Africa is a major current concern. To line 471, I would add a sentence referring to how and why the results in the current study don't likely capture the current situation in Africa, referring to sample dates. And then again refer to sample dates in the sentence spanning lines 479 to 483. On a different but related note, the authors refer to full artemisinin resistance (line 479). What is meant by full resistance? I was not aware of reports of full artemisinin resistance.

Thank you, we have added this to line 882. Regarding full and partial artemisinin resistance, we have fixed this sentence. We initially referred to fully validated variants in the WHO catalogue, and the wording got confused.

Minor suggestions

1. Figure 1: I don't understand Figure 1B or the reference to it within the caption. What insight is Fig1B adding above and beyond Fig1A and Fig1C? Where is Fig1B referred to in the main text? Why are the axes in Fig1B PC1 and PC2 and not D1 and D2 as in Fig1A? What is the maximum k value of an individual isolate? Why does the caption mention Admixture in between (A) and (B)?

   Thank you, we have revised this figure as per Reviewer 1. Figure 1B refers to the clusters (K) observed in the admixture analysis, demonstrating that the ancestral clusters overlap with geography. We have removed maximum K from the legend title and refined what this means (line 393). We have re-visualised the figure relabelling 'PC' to 'D' as this is a multidimensional scaling analysis.

2. Figure 3: the number of categories is not amenable to visualisation. I would reduce the number of categories to those with visible proportions plus "other" which can be defined in the caption.

Thank you for this feedback. As per the suggestions from Reviewer 1, we have replaced this figure with a drug-resistant haplotype analysis.

3. Figure 4: IBD networks suggest intra-mutation associations progressively replace intra-country associations. However, these associations are likely confounded by imbalanced

sample counts in different time points. Sample counts should be added to the per-mutation and per-country legends. In addition, reference to Sup Fig 15 in the caption of Fig 4 would aid interpretation of Fig 4B (it is not obvious from Fig 4 alone that the strong association between other K13 mutations in 2012-2015 is possibly explained by strong intra-Vietnam relatedness).

Thank you for this suggestion. Sample counts and a reference to the relevant figure have been included in the figure legend to improve clarity (lines 940-946).

4. Sup Fig1: label-to-cluster links are unclear, e.g., which cluster does 'Venezuela' refer to? An opaque version of the cluster colour could be used to outline the cluster and print the label.

Thank you, the lines within the figure now point directly to the relevant cluster, and an opaque colour has now been used.

5. Supplementary Figure 8: I like this figure. Does it depict the top 5% (line 250) of all high-IBD regions or the top 5% per region? It would be nice to annotate the positions of the named loci discussed in the paragraph beginning line 256.

Thank you for the positive feedback. We have annotated the loci in the figure.

6. The addition of a very brief intuitive description of each analysis each time a new one is introduced (already done for Tajima's D) would help readers interpret results; e.g., adding a few words to the parenthesis on line 154 "(MDS — a clustering analysis…)", adding a short parenthesis after "Gene Ontology (GO) term overrepresentation analysis" on line 256; coping the phrase "whereby … in the analysis" on lines 769-770 to line 32. Likewise, reference to H12 (line 452) is meaningless without extra info: at the very least, add a reference.

Thank you, we have refined the introduction of analyses (e.g. line 462) where additional clarification was needed.

7. I don't understand the threshold on line 345 (e.g., why write 1 – 2 and not -1? What does the conditioning bar "|" represent?). Without additional information its meaningless. I suggest the reviewers either unpack it or move it to the methods.

Thank you, this is a standard form of iHS threshold calculation. We have moved this to the Methods section and refer to it as an iHS score for clarity.

8. I really appreciate the cautious language. Nevertheless, there are still some places that could be tempered: line 240 ("likely reflect"), line 346 ("were considered to be under recent positive selection"); line 462 ("prevalence of genetic markers of resistance"); line 610 ("likely shaped"); line 612 ("likely reflecting").

We appreciate the need to be cautious and have updated all lines accordingly to indicate more careful interpretation is needed.

9. Line 391, 395: does sites refer to SNPs? If so, I would stick with SNPs.

We have referred to sites as 'SNPs' throughout.

10. I was surprised by the results on lines 444 and 445: I expect evidence of balancing to be greatest in high-transmission. Can the authors expand on this? Supplementary Fig 11: it might be nice to delimit -2 and 2 with e.g., a central shaded band. West Africa is almost entirely below the -2 limit. Do these limits make sense for genome-wide scans? It looks like there might be some statistical artefact that requires a per-region adjustment.

This is a very helpful suggestion. We have adjusted the analysis to report on the bottom 5% and top 95% percentiles for Tajima's D. This per-region adjustment has been useful for suggesting that *pfkelch13* contains a high number of rare variations, consistent with positive selection (lines 826-828). We have updated Supplementary Figure S11 to reflect this.

11. Lines 637 to 641: Do these statements agree with incidence data where available?

We have highlighted where our results are supported by available incidence data (e.g., line 1210 summarising ACT failure for DRC). However, it is not always available to support each time point.

12. I don't understand lines 753-754: are heteroallelic calls with MAF < 20% reduced to homoallelic calls? Please re-phrase.

Thank you, these lines have been rephrased (lines 1441-1442).

13. Were multiclonal isolates removed from all but Fws analyses? Might this bias any of the conclusions?

We have clarified which analyses removed 'polyclonal' isolates. The removal of polyclonal samples is important for these analyses as their inclusion can affect the underlying assumptions in the selection, IBD and other analyses (lines 1293-1294).

14. Line 796: "Hidden Markov model" (delete chain)

The word 'Chain' has been removed.

15. Reviewer #2 (Remarks on code availability):I have only glanced at the github repository. I have not tried to run the code. The documentation is excellent.

Thank you for the positive feedback.

Reviewer 3

The authors have presented data on 17,565 Plasmodium falciparum isolates collected from 39 different malaria endemic countries of the globe and performed several population genetic analyses under different models to infer P. falciparum's evolution, patterns of gene flow and genetic patterns of drug resistance in these populations. While they have reconstructed history of P. falciparum populations, they also have unravel signatures of natural selection in drug-resistant loci. With these findings, they interpreted that the knowledge will impact

malaria control efforts and also guide malaria control strategies. These analyses are extensive and the dataset is substantial. However, several concerns remain to be addressed, especially regarding novelty and completeness of this study.

1. Across the P. falciparum populations, based on published literate at local level, many of the factual history of population structure, gene flow, isolation-by-distance, etc. that the authors have presented are already known. These are principally driven consequences of P. falciparum's transmission dynamics, which is fundamentally different from those of, Plasmodium vivax. For example, the finding on the location of the genetic loci/ nucleotide sites under selection are located in highly variable sites are hallmark of the evolutionary principles. Furthermore, it is well known that the surface antigenic genes in malaria parasites in general, and P. falciparum in particular are known to be highly variable and under strong diversifying selection. Similarly, loci involved in host-parasite interactions have been repeatedly shown to exhibit strong selective pressure in both host and parasitic genomes (e.g. the DARC in humans, and PvDBP P. vivax). Therefore, the observation on selection hot-spots at the chromosomal level does not represent a novel contribution in itself, while accurately described.

We thank the Reviewer for noting that many features of *P. falciparum* population structure and selection have been previously described. To address this and to increase the resolution of our analyses, we re-ran all selection analyses after removing hypervariable regions and known highly polymorphic surface antigen genes, thereby reducing confounding effects from these loci. Our results reveal several novel insights. Identity-by-descent analyses found higher fractions of IBD in samples from low-transmission areas, thereby highlighting localised relatedness patterns. Of particular interest, in the Horn of Africa, *KIC9* and *KIC7* loci emerged as regions of elevated IBD, which may form a genetic background conducive to the emergence of artemisinin resistance (line 481). Across other regions, drug-selective loci including *pfcrt, pfdhps,* and *pfmdr1* showed strong local signals of IBD, reflecting ongoing selection pressures from antimalarial use.

Using additional selection statistics (iHS and XP-EHH), we observed loci potentially shaped by local adaptation, including gene products linked to drug resistance, virulence, and epigenetic regulation such as *BDP4* and *pfcrt*. Notably, we detected consistent signals around *pfubp1* (Chr01 170–200 kb) in South Asia, West Africa, and East Africa, a gene increasingly implicated in artemisinin resistance. In Central Africa, additional regions under selection included *DHODH (PF3D7_0603300),* PF3D7_1025000 (Eps15-like protein), and MCA2 (PF3D7_1438400), all involved in drug-resistance pathways (line 602-717). These findings highlight genetic backgrounds that may contribute to reduced artemisinin susceptibility beyond the *pfkelch13* mutations. This is an important finding as countries such as the DRC have reported widespread ACT failure, and a lack of association with *K13* mutations (Yobi *et al.,* 2020).

Taking on board feedback from Reviewer 2, we also refined our Tajima's D analysis to show regions in the bottom 5% and top 95% percentiles per geographic region. Here,

we observed evidence consistent with positive selection (excess of rare alleles) in Southeast Asia and Oceania (lines826-828).

We also include deeper insights into the newly sequenced data from Brazil and Vietnam, as per suggestions from Reviewer 1. We provide additional insights from the newly sequenced samples from Brazil and Vietnam. In Vietnam (N=18), all isolates carried the *pfkelch13* C580Y mutation, with elevated IBD in the genomic region containing KIC13, a protein located in the same cellular compartment as KELCH13 (lines 1038-1066). This finding suggests a potential mechanism that may compensate for fitness costs or enhance artemisinin resistance, warranting further investigation.

2. One of the most critical gaps in the manuscript is the absence of genomic data from P. falciparum isolates originating from India. India remains one of the most malaria-endemic countries globally, and prior studies have highlighted the unique genetic and evolutionary characteristics of Indian P. falciparum populations. For example; i) Indian P. falciparum show several ancestral properties, such as highest genetic diversity than the African P. falciparum, suggesting an ancestral origin (Tyagi and Das, 2025, Mitochondrion). Furthermore, (ii) a distinct ancestral genetic lineage, found in India, has been proposed as the missing-link in host switch of P. falciparum from non-human primates (rhesus macaque) to humans (Tyagi et al., 2014, Molecular Ecology, reviewed in Das, 2015; Trends in Parasitology). Moreover, (iii) zoonotic P. falciparum infections have been detected in Indian rhesus and bonnet macaque for the first time (Dixit et al. PLOS Neglected Tropical Diseases, 2018), pointing to a possible primate reservoir. These define unique features of P. falciparum in India, and to my opinion leaving behind this important corridor of human (and P. falciparum) ancestral migration will not justify reconstruction of evolutionary history of P. falciparum at the global level.

We thank the Reviewer for highlighting the importance of Indian *P. falciparum* populations and for citing the key studies demonstrating their unique evolutionary history. We agree that India represents a critical region for understanding global parasite diversity, including ancestral lineages, potential primate-to-human transmission links (Tyagi et al., 2014; Das, 2015), and the high levels of genetic diversity recently described (Tyagi & Das, 2025). In the current study, our analysis was limited to publicly available whole-genome data that met strict quality thresholds. We have explicitly acknowledged this limitation in the revised manuscript (lines 1338-1361), including the excellent references suggested. In the original manuscript, we incorporated the ~300 high-coverage Indian samples available in MalariaGEN Pf7 into our analyses, which are part of the current analysis. We agree that greater sampling from India would be an important future direction for reconstructing parasite origins and migration pathways, and we now emphasise this as a priority area for future research.

Reviewers' comments:

Reviewer #1 (Remarks to the Author):

We thank reviewer 1 for their useful suggestions to improve the manuscript and provided a point-by-point response below.

The authors have responded thoroughly to my original comments and have clarified many aspects of the manuscript. However, the novel aspects of this manuscript remain a minor component of the overall document, and their value is unclear because they are under-explored.

For example, the abstract now references the detection of previously unreported selection signals at DHODH, Eps150-like protein, and MCA2, loci previously associated with drug resistance in various contexts. However, these selection analyses are described in supplementary figures rather than main figures, while the main figures retain their focus on findings that are not original or surprising, such as the observation of greater Fst between continents than within continents (Figure 2), or the association of clonality with the emergence of K13 mutations in SE Asia (Figure 4).

We thank the reviewer for this suggestion and agree that it is important to highlight the novelty of our results, particularly by bringing them to the forefront of the manuscript (see description below). These updates ensure that the selection results presented in **Figures 2 and 3** and supplementary tables reflect robust, multi-metric evidence, highlighting both known drug-resistance loci and novel candidate regions. Previous Figures 2 and 4 are now presented as supplementary figures to ensure novel results are highlighted (**Supplementary Figures 14 and 19)**.

The novel results are only explored superficially, and therefore it remains unclear if readers should ascribe value to them. For example, the selection signal at DHODH appears to take the form of a significant iHS p value for Central Africa. Does the iHS selection at DHODH signal associate with a concordant enrichment in IBD in that population, or a particularly low Tajima's D value? Are there nonsynonymous polymorphisms with unusually high minor allele frequency in DHODH in Central Africa, or what could be driving the iHS result? Why do the authors speculate there would be selection on DHODH, when the small molecules that have been observed to drive resistance mutations at DHODH have not been approved for clinical use?

The focus of the paper should be changed to more deeply exploring novel observations to warrant publication.

Thank you for this suggestion. Taking your advice, we updated our selection analyses to focus on composite signals of positive selection, integrating Tajima's D, IBD, iHS, XP–EHH, and F_{ST} results across 10-kb windows (**Results: lines 808-903 and Methods: lines 1325-1337**). Regions were classified as weak, moderate, or high support based on concordance across multiple metrics, providing a more robust framework for detecting subtle and partial selection. We further overlaid F_{ST} and annotated SNPs to genes to identify differentiated alleles and extended haplotypes, strengthening the biological interpretation of the signals. We adapted our approach to ensure that the selection analyses were robust to smaller sample sizes, increasing the minor allele frequency (MAF) threshold in Central Africa to 0.05 to ensure results are robust. We have included additional discussion of genomic regions within drug pathways that are not yet in routine clinical use (**lines 1074-1093**). Novel results are now presented in **Figures 2 and 3**.

Reviewer #2 (Remarks to the Author):

We thank reviewer 2 for their helpful suggestions to improve the manuscript and provided a point-by-point response below.

The authors have addressed my points, but they should make the relative contribution of this study relative to other global analyses clearer upfront:
1. Add citations for the major "earlier global analyses" in the last paragraph of the Background and refer the reader to Supplementary Table 19.

Thank you we have added these on line 144.

2. In the Background state something along the lines of "Although this study goes into more analytic detail compared with other global analyses, the global analysis of MalariaGEN 2025 represents a larger and more up-to-date global data set"

Thank you, we have updated lines 122-124.

3. In the first section of the results: in addition to showing how the 17,565 samples break down geographically, describe how they break down into previously analysed datasets (XX in Pf7, XX in Pf8 but not Pf7, plus 76 analysed here first).
Thank you we have added these in on line 147-149.

In addition, some minor suggestions:

4. Abstract "Polygenic backgrounds associated with artemisinin resistance" on line 46 implies the manuscript has in vitro / clinical data on resistance. Do you mean "Polygenic backgrounds associated with genetic markers of artemisinin resistance" ?
Thank you we have removed this sentence in the abstract and updated it to reflect our improved results.

5. Line 95: add artemisinin: "signs of reduced artemisinin combination therapy (ACT)"
This has been updated on line 105.

6. Fig 1: Why do some points coloured with the grey of Oceania in panel A disappear in panel B?

Thank you for this helpful suggestion. We have updated Fig 1 to resolve the missing samples.

7. Fig 1 caption: Points are coloured, not "Dimensions"
Thank you, the figure legend for Fig 1 has been amended (line 201).

8. Line 155 and Supp. Fig 1: I don't see any samples with a colour assigned to Ecuador, only Colombia.

Thank you, we have updated Supp Fig 1 to include Ecuador.

9. Supp. Fig 1: caption mentions non-existent panel B
Thank you this has been removed from the caption.

10. Line 172: I don't see any SE Asian isolates that exhibit Wast (East) African Ancestry in Fig 1.
Thank you we have removed this sentence (see line 188).

11. Section entitled "Integration of New Regional Datasets into Global Population Genomics": please site the studies detailing sample collection for the 76 new sequences.
Thank you, we have added the sample collection into the methods (lines 1207-1215).
